# Donor-Acceptor Substituted Benzo-, Naphtho- and Phenanthro-Fused Norbornadienes

**DOI:** 10.3390/molecules25020322

**Published:** 2020-01-13

**Authors:** Mads Mansø, Lorette Fernandez, Zhihang Wang, Kasper Moth-Poulsen, Mogens Brøndsted Nielsen

**Affiliations:** 1 Department of Chemistry, University of Copenhagen, Universitetsparken 5, DK-2100 Copenhagen Ø, Denmark; madsmansoe@gmail.com; 2 Department of Chemistry and Chemical Engineering, Chalmers University of Technology, Kemivägen 10, 41296 Gothenburg, Sweden; fernandez.lorette11@gmail.com (L.F.); zhihang@chalmers.se (Z.W.); kasper.moth-poulsen@chalmers.se (K.M.-P.)

**Keywords:** aromaticity, chromophore, cycloaddition, norbornadiene, photoswitch, polycyclic aromatic hydrocarbon, ring strain, solar energy storage, Sonogashira coupling, quadricyclane

## Abstract

The photochromic norbornadiene/quadricyclane (NBD/QC) couple has found interest as a molecular solar thermal energy (MOST) system for storage of solar energy. To increase the energy difference between the two isomers, we present here the synthesis of a selection of benzo-fused NBD derivatives that contain an aromatic unit, benzene, naphthalene or phenanthrene, fused to one of the NBD double bonds, while the carbon atoms of the other double bond are functionalized with donor and acceptor groups. The synthesis protocols involve functionalization of benzo-fused NBDs with bromo/chloro substituents followed by a subjection of these intermediates to a cyanation reaction (introducing a cyano acceptor group) followed by a Sonogashira coupling (introducing an arylethynyl donor group, -C≡CC_6_H_4_NMe_2_ or -C≡CC_6_H_4_OMe). While the derivatives have good absorption properties in the visible region (redshifted relative to parent system) in the context of MOST applications, they lack the ability to undergo NBD-to-QC photoisomerization, even in the presence of a photosensitizer. It seems that loss of aromaticity of the fused aromatics is too significant to allow photoisomerization to occur. The concept of destroying aromaticity of a neighboring moiety as a way to enhance the energy density of the NBD/QC couple thus needs further structural modifications, in the quest for optimum MOST systems.

## 1. Introduction

**Scheme 1:**
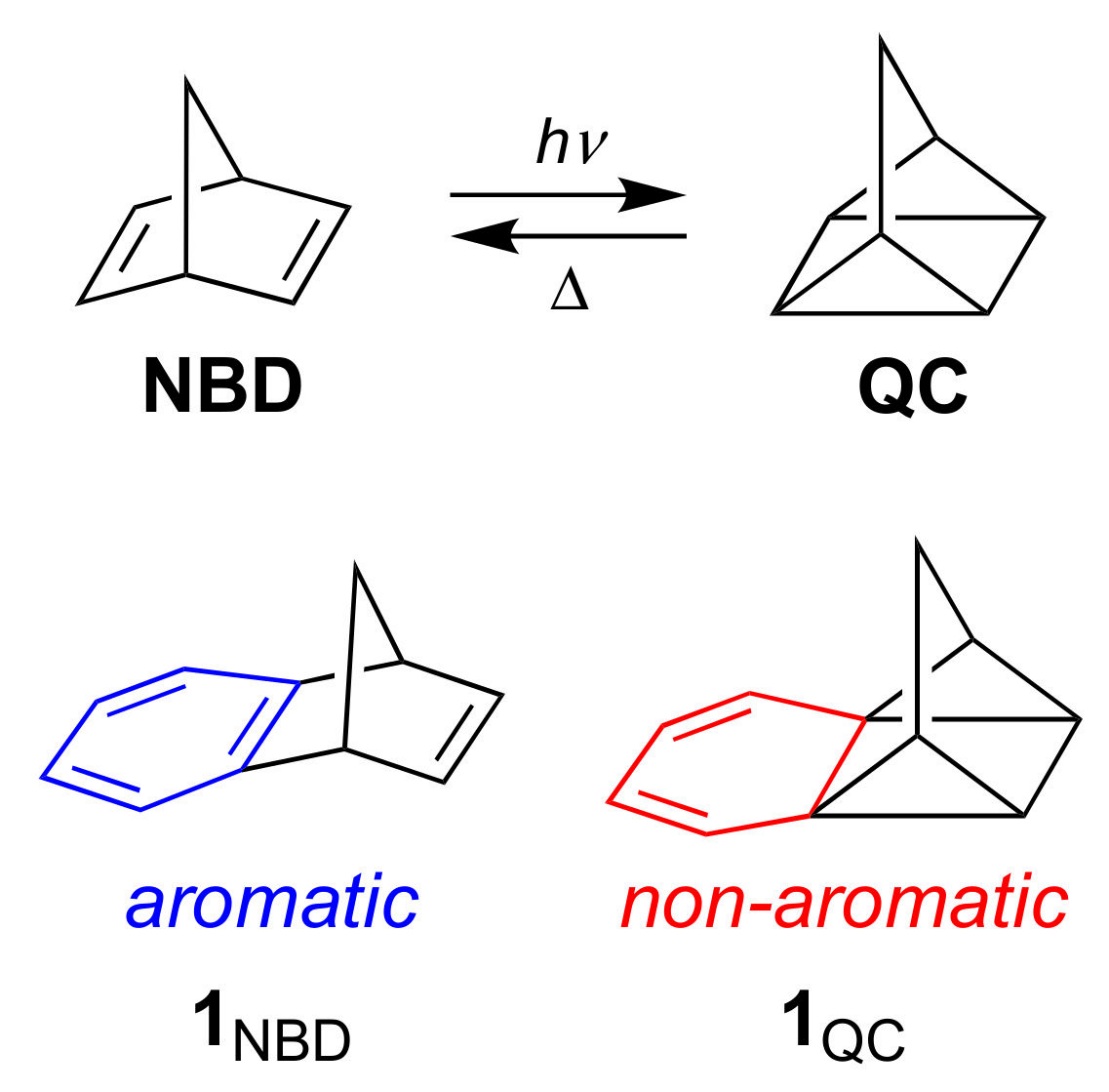
Norbornadiene/quadricyclane (NBD/QC) interconversions and benzo-fused derivatives.

Functionalized norbornadiene/quadricyclane (NBD/QC) couples have been identified as promising candidates for molecular solar thermal energy storage (MOST) systems that can undergo a closed energy cycle of light absorption, energy storage and energy release [1,2,3]. NBD undergoes a [2 + 2] cycloaddition upon irradiation, forming a strained high-energy QC isomer (Scheme 1). By introduction of donor-acceptor substituents on one or both double bonds of NBD, its absorption maximum can conveniently be redshifted from the UV to the visible region [4,5,6], thereby better matching the solar spectrum. This functionalization, however, decreases the energy density (from the optimum 1 MJ kg^-1^ of the parent system) on account of the increased molecular weight of the molecule. Yet, by using the same aryl group as a bridging substituent between two NBD units, this decrease in energy density can to some extent be counter-balanced [7].

We became interested to investigate the possibility for other structural approaches of increasing the energy density. One approach could be to couple the isomerization reaction with loss of aromaticity of a fused benzene ring as shown for the benzo-fused couple **1**_NBD_/**1**_QC_ in Scheme 1. Yet, irradiation of derivatives of **1**_NBD_ with various substituents at the benzene ring did not result in QC formation but instead in rearrangement reactions [8,9,10]. Along this line, some of us recently investigated benzo-fused dihydroazulene/vinylheptafulvene (DHA/VHF) derivatives [11,12]. For this photo-thermoswitch couple, benzannulation was also, in most cases, accompanied by the loss of the desired photoactivity of the DHA photochrome. A theoretical study showed that loss of aromaticity in the excited state reaction profile inhibits the DHA-to-VHF photoswitching [13]. In consequence, it seems that we need to seek a compromise by designing systems with less significant loss of aromatic character during photoswitching. One solution could potentially be to fuse a polycyclic aromatic hydrocarbon to the photochrome instead of just a single benzene ring.

**Figure 1:**
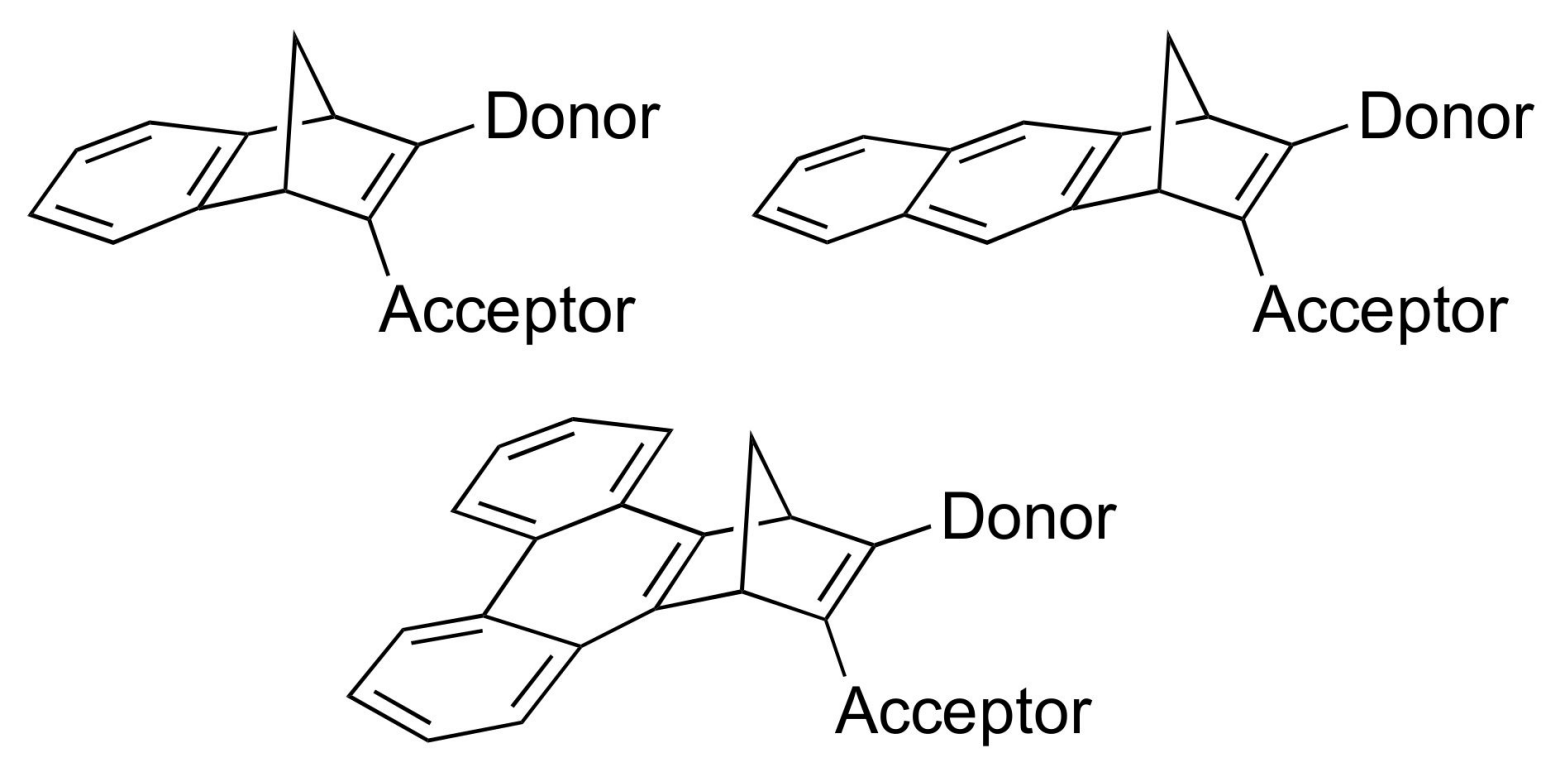
Norbornadiene target molecules with fused aromatic units at one double bond and donor-acceptor substituent groups at the other double bond.

Here we explore further the influence of benzannulation by fusing a benzene, naphthalene and phenanthrene unit to the one double bond of NBD, while at the same time maintaining an absorption in the visible region by donor-acceptor substitution at the other double bond, providing target molecules of general structures shown in Figure 1. As acceptor unit, we chose a cyano group, while *N*,*N*-dimethylanilino and anisyl groups connected via different bridges were chosen as donor groups. We present synthetic protocols for achieving such new derivatives and their properties.

## 2. Results

### 2.1. Synthesis

**Scheme 2:**
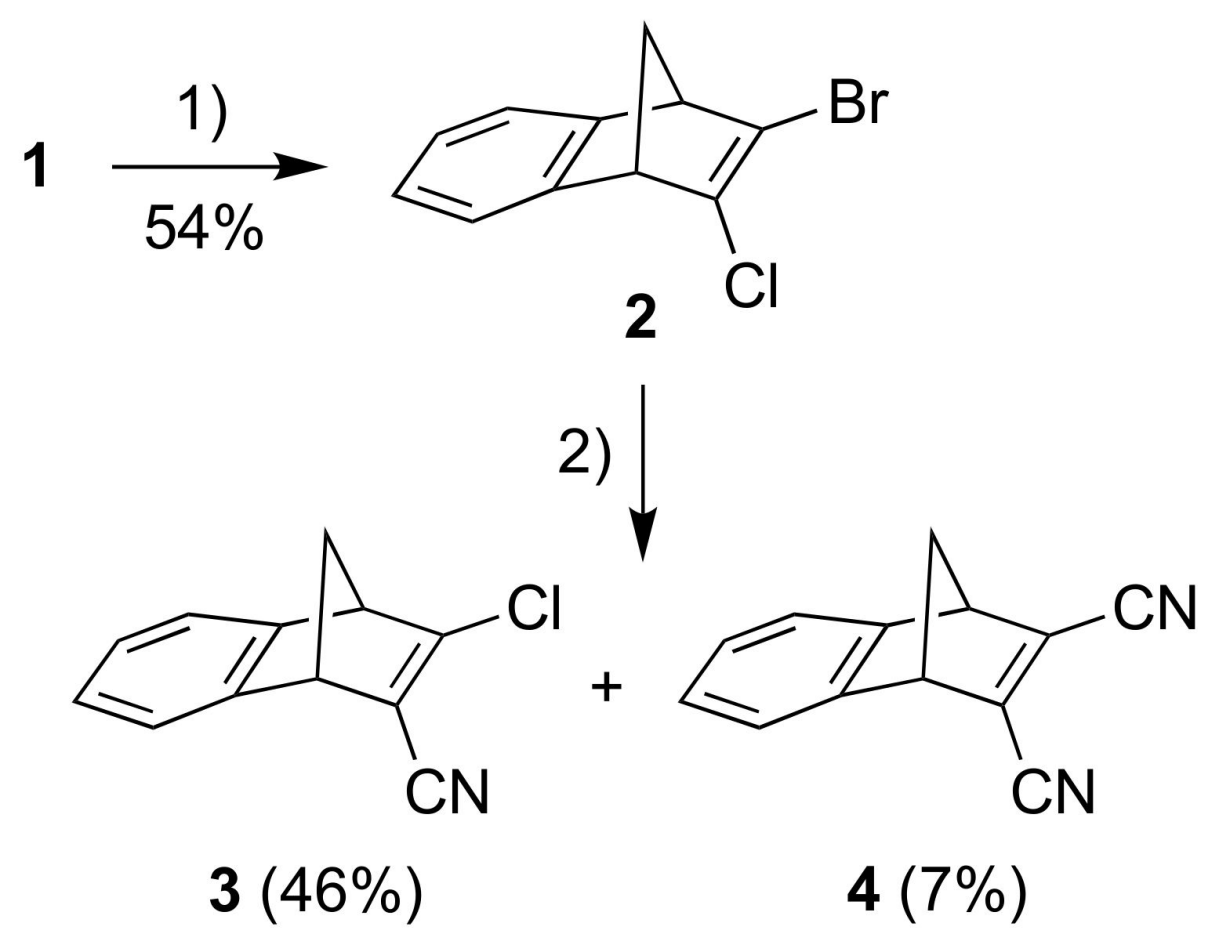
Reagents and conditions: (**1**) i. *t*-BuOK, THF; ii. BuLi, −84 °C to −41 °C; iii. TsCl, −84 °C; iv. BuLi, −84 °C to −41 °C; v. TsBr, −84 °C to rt; (**2**) CuCN, DMF, 105 °C.

A one-pot procedure for introducing a bromo and chloro substituent at the one double bond of NBD has previously been described [14] and the resulting 2-bromo-3-chloronorbornadiene was used as a convenient precursor for incorporating donor and acceptor functionalities [6]. Subjecting in a similar manner the known [15] starting material **1** to the conditions shown in Scheme 2, we obtained the new chloro/bromo-substituted building block **2**. A cyanation reaction of this compound using CuCN in DMF (in analogy to conditions used for cyanation of the parent 2-bromo-3-chloronorbornadiene [6]) subsequently gave the product **3** where one cyano acceptor group has been introduced (Scheme 2). A small amount of the product **4** resulting from twofold cyanation was also isolated in this reaction.

**Scheme 3:**
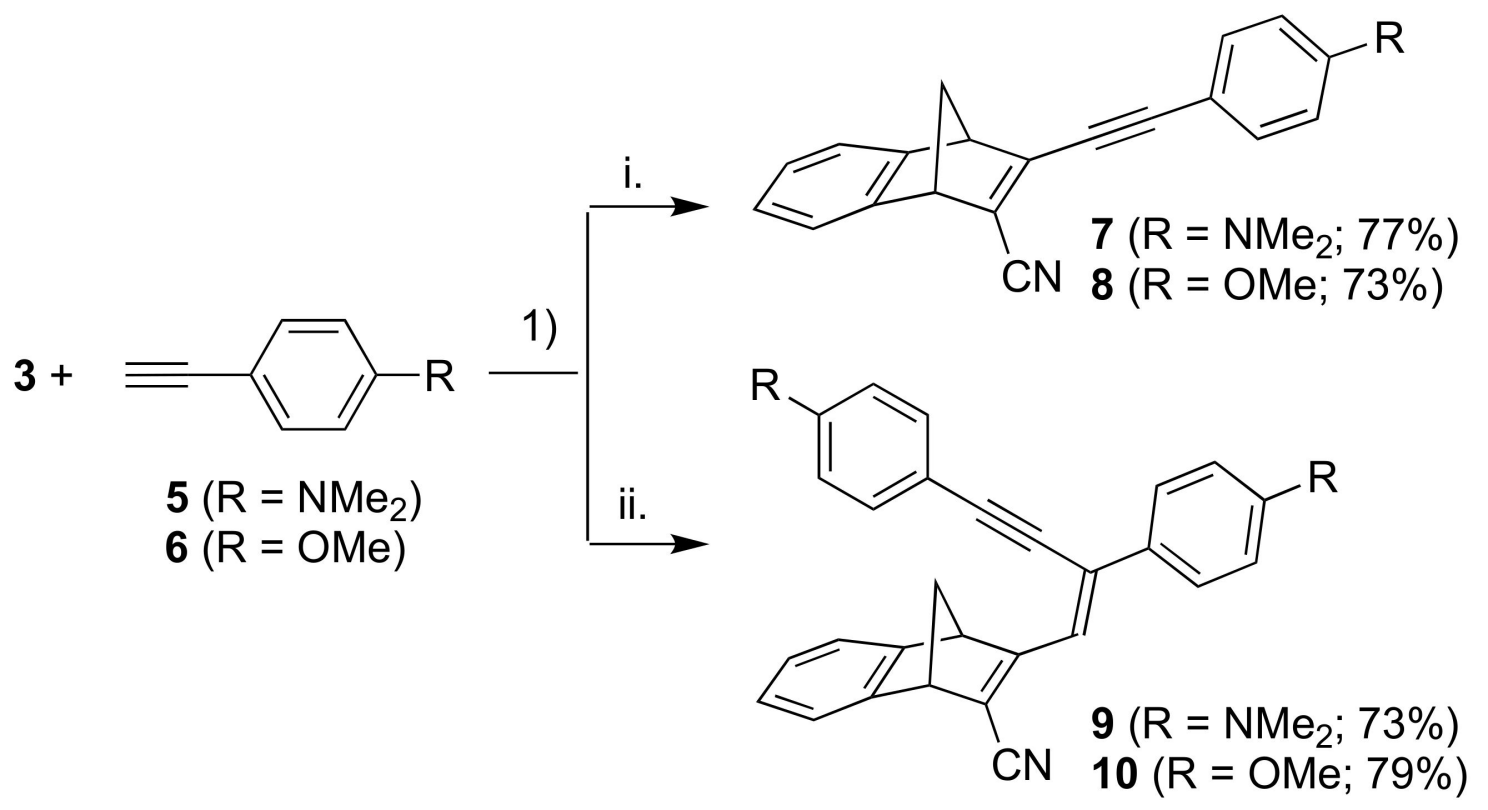
Reagents and conditions: (**1**) Pd(PPh_3_)_2_Cl_2_, CuI, THF/Et_3_N; i. alkyne: 1 equiv.; ii. alkyne: excess.

Next, we introduced anilino and anisyl donor groups by Sonogashira coupling reactions between **3** and the terminal alkynes **5** and **6**, furnishing the products **7** and **8**, respectively (Scheme 3). By employing more than two equivalents of the terminal alkynes, we obtained instead the products **9** and **10**. The two products **9** and **10** bear a resemblance to previously reported compounds obtained from Sonogashira coupling with 3-iodoflavones and 3-iodothioflavones, and it is believed that this is due to a second palladium catalyzed coupling step [16].

**Scheme 4:**
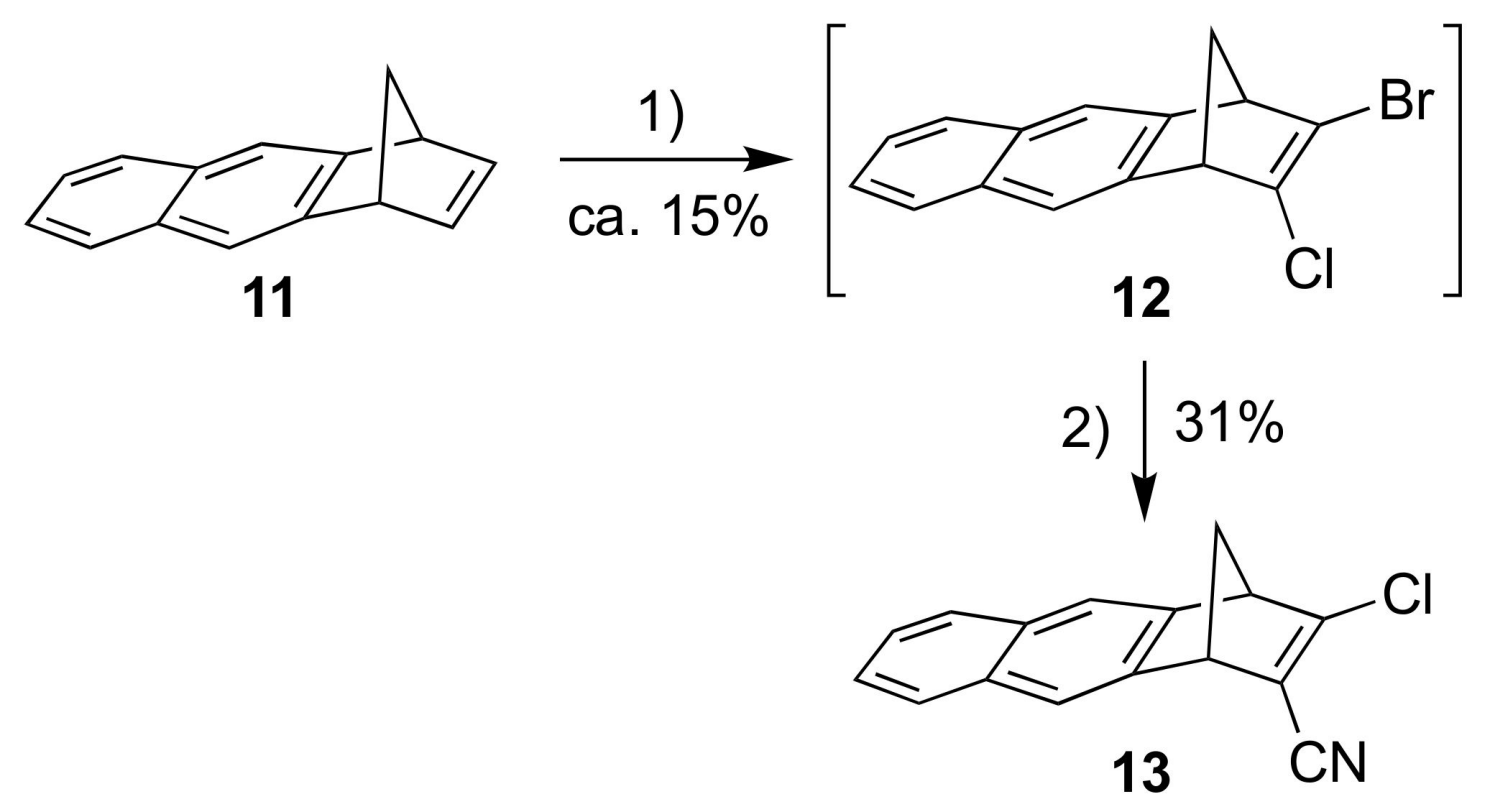
Reagents and conditions: (**1**) i. *t*-BuOK, THF; ii. BuLi, −84 °C to −41 °C; iii. TsCl, −84 °C; iv. BuLi, −84 °C to −41 °C; v. TsBr, −84 °C to rt; (**2**) CuCN, DMF, 100 °C.

**Scheme 5:**
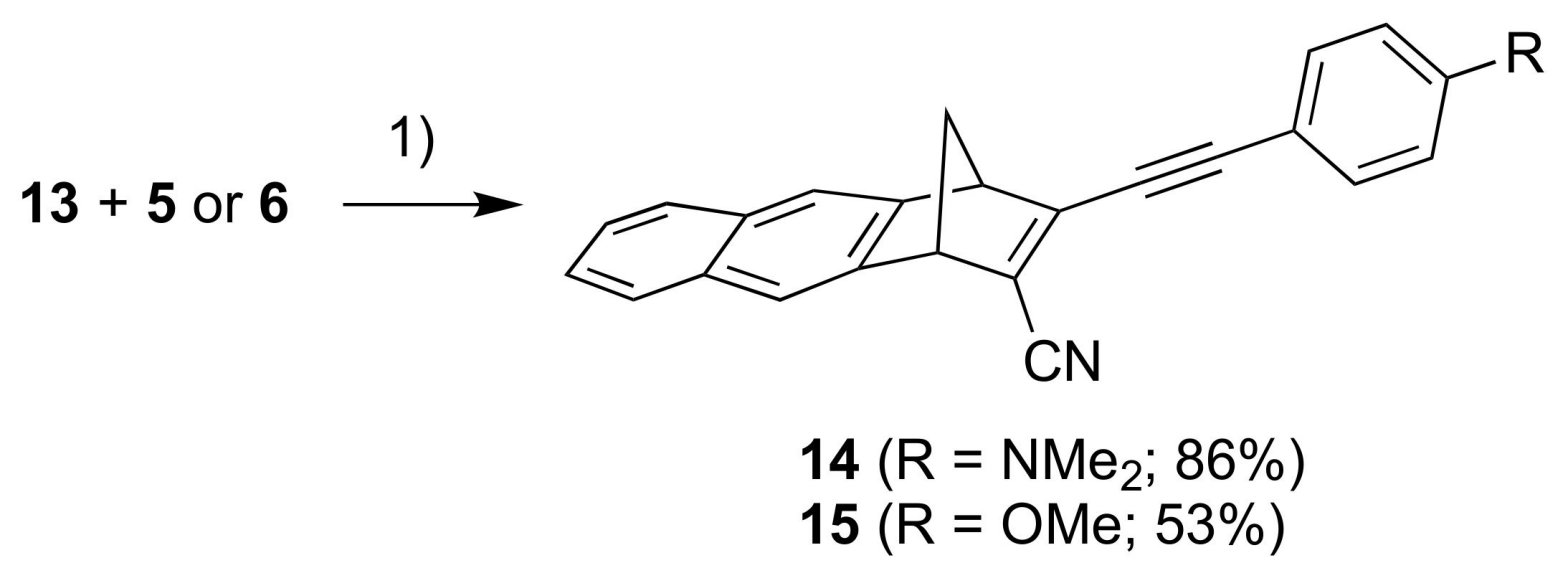
Reagents and conditions:(**1**) Pd(PPh_3_)_2_Cl_2_, CuI, THF/Et_3_N.

Our next objective was to expand the aromatic unit to a naphthalene moiety. First, compound **11**, prepared according to literature procedure [17], was subjected to a similar reaction sequence as above, providing the bromo/chloro derivative **12** (Scheme 4) (as an inseparable mixture with starting material **11**). A subsequent cyanation reaction gave the product **13**. Sonogashira couplings between **13** and alkynes **5** and **6** gave the donor-acceptor substituted products **14** and **15**, respectively (Scheme 5).

**Scheme 6:**
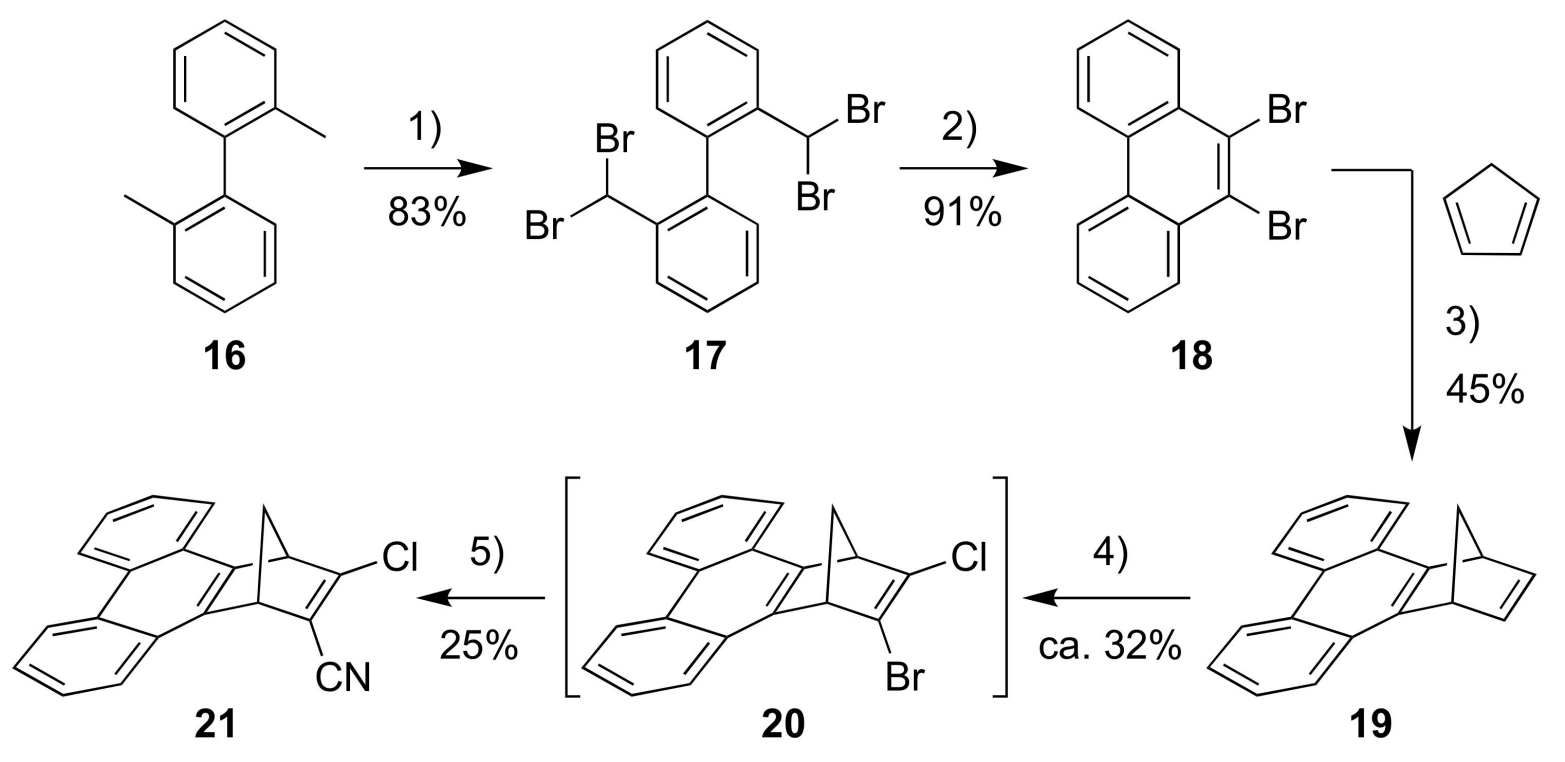
Reagents and conditions: (**1**) 1-bromopyrrolidine-2,5-dione, benzoyl peroxide, PhH, reflux; (**2**) *t*-BuOK, DMF, 0 °C; (**3**) BuLi, PhMe; (**4**) i. *t*-BuOK, THF; ii. BuLi, −84 °C to −41 °C; iii. TsCl, −84 °C; iv. BuLi, −84 °C to −41 °C; v. TsBr, −84 °C to rt; 5) CuCN, DMF.

**Scheme 7:**
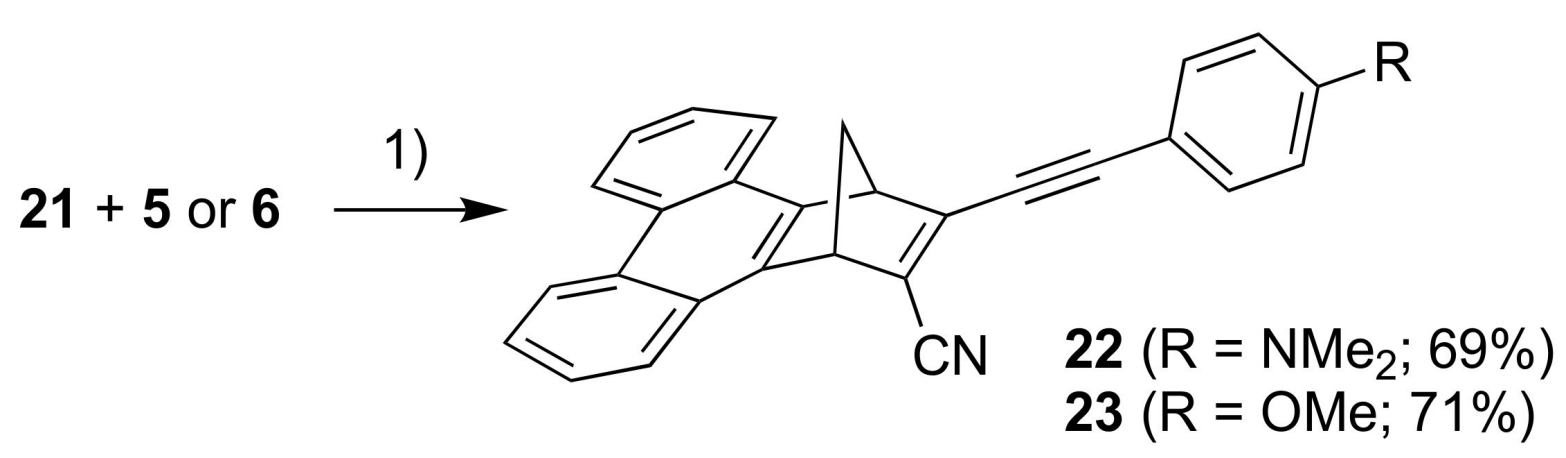
Reagents and conditions: (**1**) Pd(PPh_3_)_2_Cl_2_, CuI, THF/Et_3_N.

For introducing a phenanthrene unit, we first had to develop a protocol for fusing this polycyclic aromatic hydrocarbon to NBD as shown in Scheme 6. First, 2,2′-dimethyl-1,1′-biphenyl (**16**) was converted into the tetrabromide **17** by radical bromination and, upon subsequent treatment with strong base, the dibromo-substituted phenanthrene **18** resulted. This compound was turned into the corresponding benzyne derivative that was reacted in a cycloaddition reaction with cyclopentadiene to give the NBD derivative **19**. This compound was then converted into the bromo/chloro derivative **20** (isolated as an inseparable mixture with **19**) that was subsequently converted into the cyano compound **21**. Sonogashira couplings finally gave the donor-acceptor derivatives **22** and **23** as shown in Scheme 7.

### 2.2. Optical and Switching Properties

**Figure 2:**
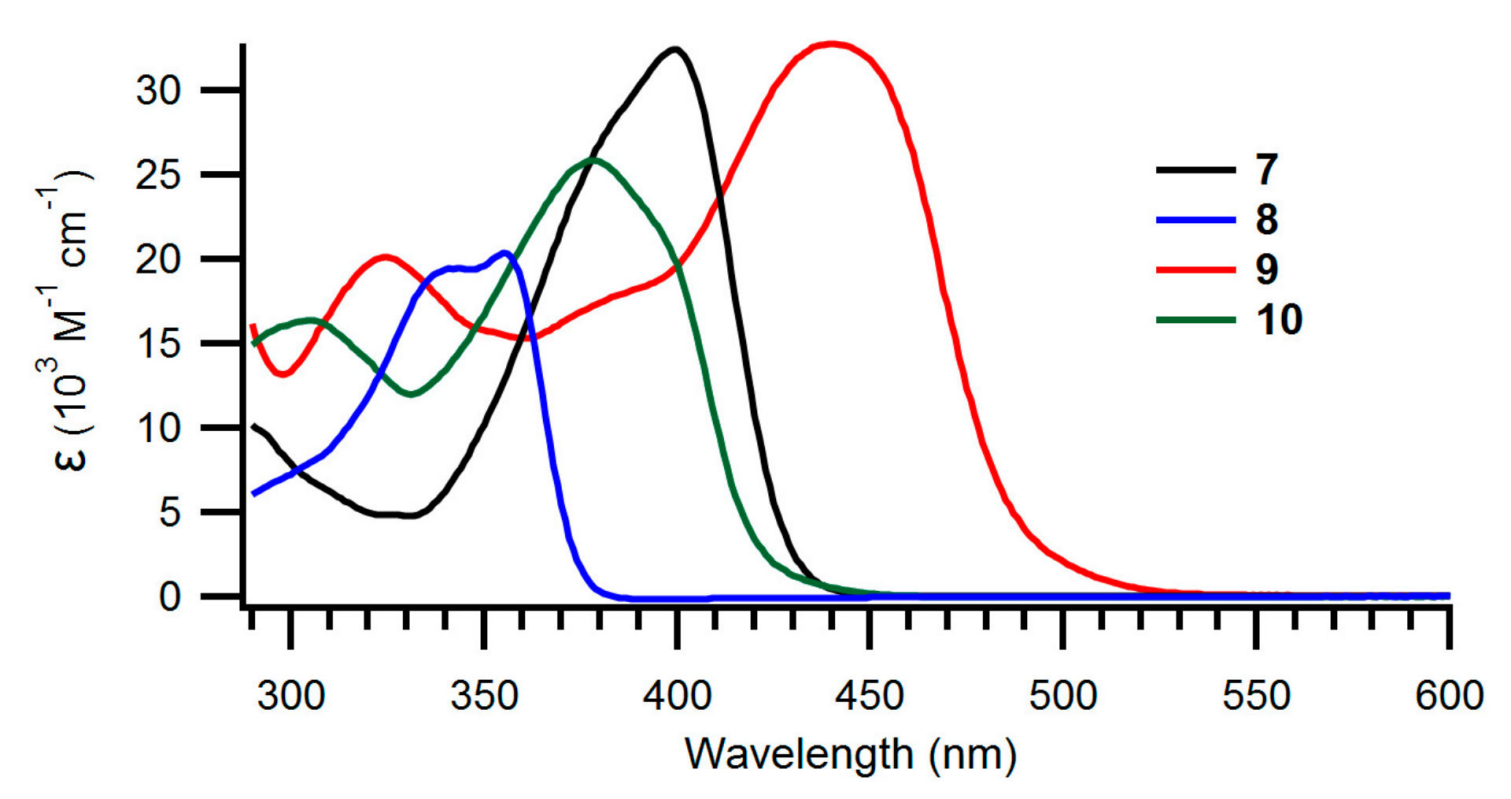
UV-Vis absorption spectra of benzo-fused NBDs **7**–**10** recorded in toluene.

**Figure 3:**
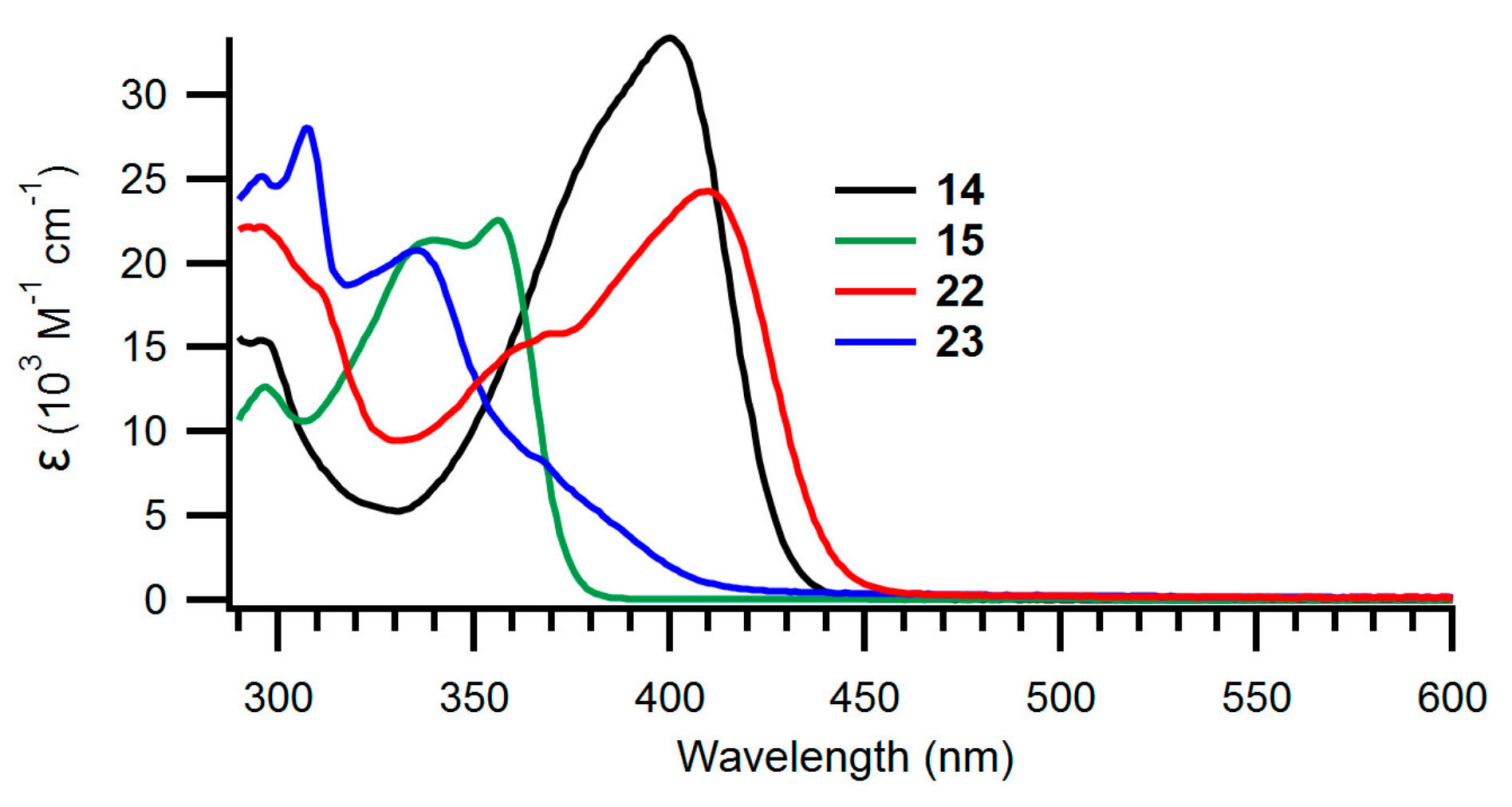
UV-Vis absorption spectra of naphtho- and phenanthro-fused NBDs **14**, **15**, **22** and **23** recorded in toluene.

The UV-Vis absorption spectra of the new NBD derivatives are shown in Figure 2; Figure 3 and longest-wavelength absorption maxima are listed in Table 1. Comparison of compounds **8** and **15** as well as compounds **7** and **14** shows that it has little influence on the absorption spectra whether the NBD is benzo- or naphtho-fused. Instead, compounds **9** and **10** with expanded donor units exhibit significantly redshifted absorptions, owing to their significantly larger π-conjugated system. It is seen that the spectra are changed significantly when the fused aromatic group is changed for a phenanthrene unit in the case of **22** and **23**.

**Figure 4:**
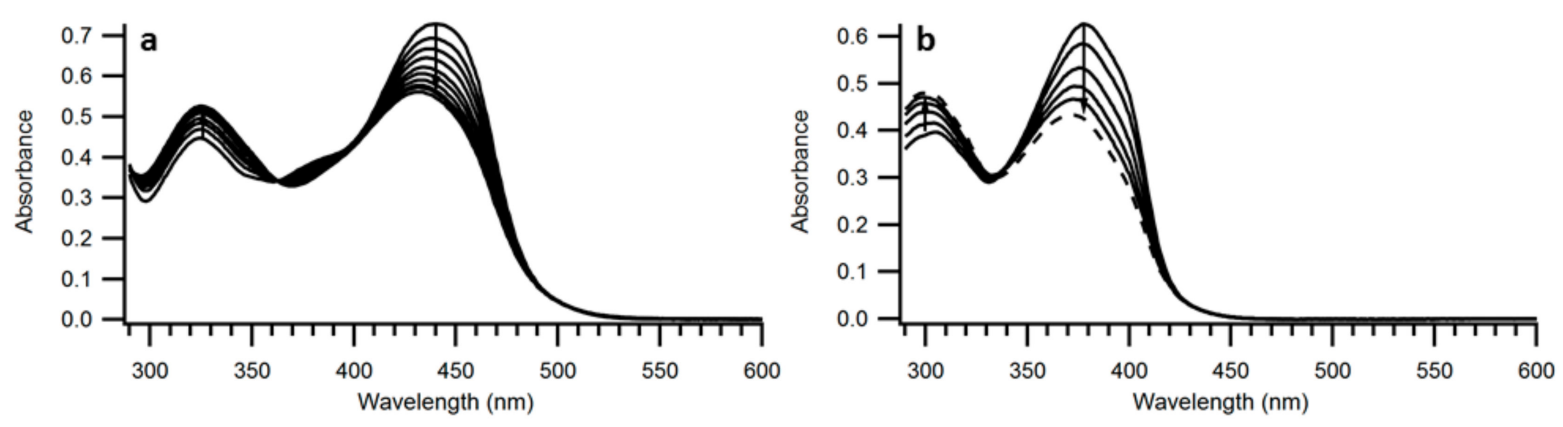
UV-Vis absorption spectra of (**a**) **9** in toluene during irradiation with 440-nm light, (**b**) **10** in toluene during irradiation with 378 nm light.

**Figure 5:**
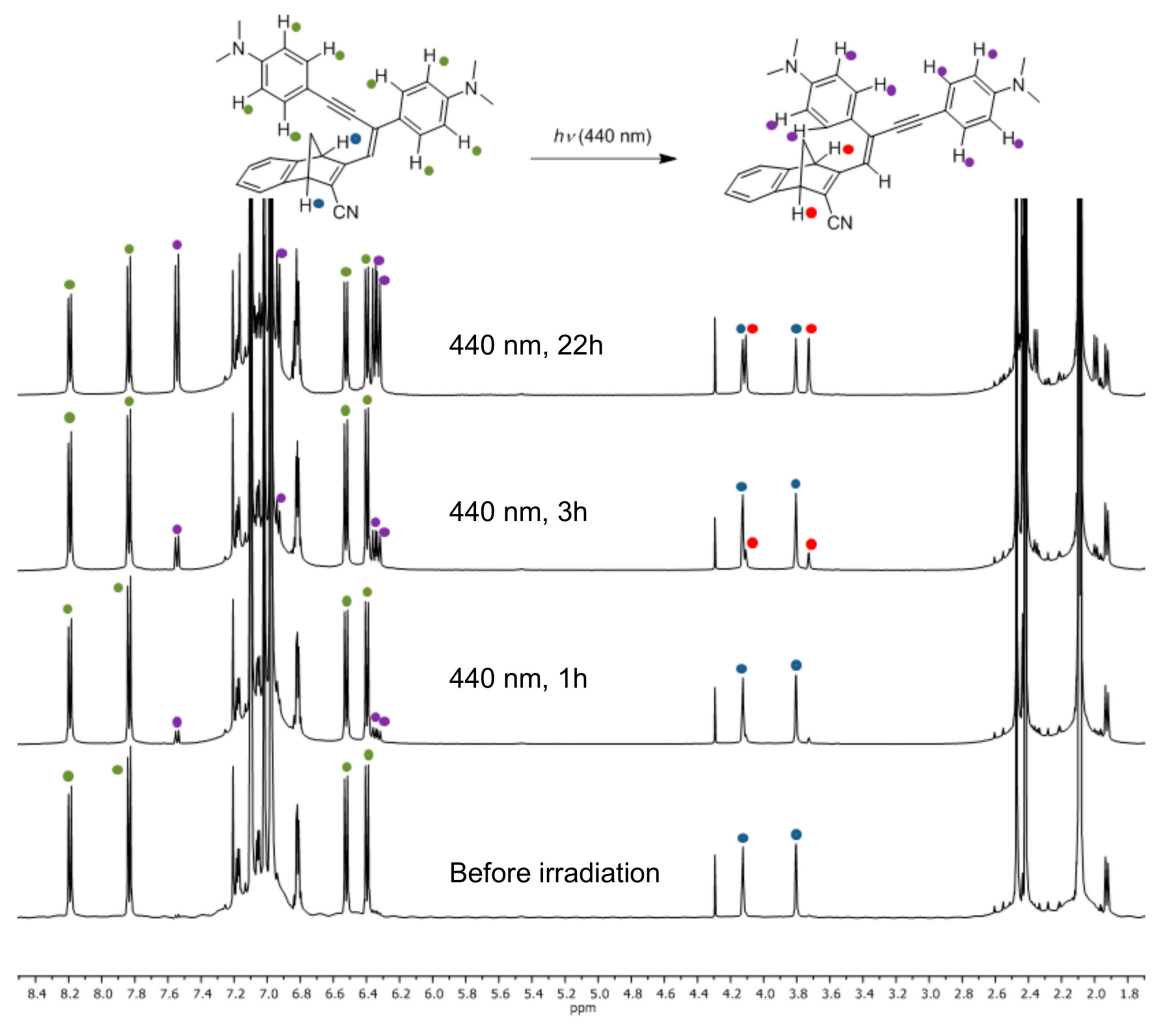
^1^H-NMR Spectra (toluene-*d*_8_, 500 MHz) of **9** recorded after various times of irradiation (440 nm).

The structures of compounds **9** and **10** were confirmed by HSQC, HMBC and NOE NMR spectroscopic analyses (see Supplementary Materials). The *Z*-configuration was assigned based on an NOE correlation between the vinylic proton and the closest *ortho*-proton on the phenyl group directly attached to the vinyl unit. By subjecting **9** and **10** to irradiation at their absorption maxima using a 450-W Xe lamp, the spectra changed as revealed in Figure 4; the intensity of the longest-wavelength absorption (slightly blueshifted) was found to decrease, while the intensity of the higher-energy absorption around 300 nm was found to increase. Conversions occurred with isosbestic points and by ^1^H-NMR spectroscopic analysis of compound **9** it was found that rather than NBD-to-QC photoisomerization, *Z*-to-*E* isomerization occurred. After 22 h of irradiation at 440 nm, a 1:1 ratio of *E* and *Z* isomers was obtained for compound **9** (Figure 5).

To investigate the switching properties of the benzo-fused systems **7** and **8**, they were irradiated at the longest-wavelength absorption maxima with a 450 W Xe-lamp. After irradiation for up to 60 min no sign of isomerization was observed in both cases. Switching the light source to a broad-spectrum thin-layer chromatography (TLC) lamp (365 nm), only decomposition of the compounds was observed, verified by no backreaction occurring posterior to light irradiation. A more concentrated sample (6 mM) of **7** in toluene was argon-flushed and irradiated with a 405 nm light emitting diode (LED). Following the reaction on TLC showed a complex mixture of decomposition products. For the two naphtho-fused NBDs **14** and **15**, an absorbance decrease was observed after irradiation at the longest-wavelength absorption maxima with a 450-W Xe-lamp but no backreaction was seen posterior to irradiation. An NMR sample of the methoxy-substituted NBD **15** in *d_8_*-toluene was irradiated at 356 nm but after 19 h this also resulted in a complex mixture according to TLC inspection and no QC characteristic signals were seen in the complex NMR spectrum of the mixture (See ESI). For the phenanthro-fused NBDs **22** and **23** once again no signs of isomerization were observed. For all the systems **7**, **8**, **14**, **15**, **22** and **23** low temperature UV-Vis experiments using a cryostat were performed to investigate whether the lack of observed NBD-to-QC photoconversion was caused by a rapid QC-to-NBD backreaction. Unfortunately, no signs of isomerization were observed even at temperatures down to −60 °C.

### 2.3. Sensitized Switching Studies

Previously, it has been found that unsubstituted norbornadiene can be converted to quadricyclane in presence of sensitizer [18]. We note that the compounds, **7**, **8**, **14** and **15** did not lead to the corresponding quadricyclanes during direct excitation at their maximum absorption wavelength. This could in principle be due to too low-lying S1 states of these compounds. One possible alternative could be to try to convert the compounds via their triplet excited states. Michler’s ketone is a known triplet sensitizer that has been used for the conversion of other NBD compounds and therefore, the conversion of **22** and **23** with this photosensitizer was attempted. Compounds **22** and **23** were individually mixed in toluene with excess of sensitizer. Since the maximum absorption of Michler’s ketone extends from 290 nm to 381 nm, a 300 nm LED light source was used to irradiate the prepared samples. Unfortunately, no conversion was observed in both cases according to ^1^H NMR spectroscopic analysis.

## 3. Discussion

We have in this work shown that the previously one-pot procedure for functionalization of norbornadiene at one double bond can conveniently be expanded to benzo-, naphtho- and phenanthro-fused norbornadienes. This expansion has allowed the synthesis of a selection of derivatives with donor-acceptor substituent groups. The identity of the fused aromatic has little influence on the longest-wavelength absorption maximum of the compounds; this absorption maximum is rather determined by the donor and acceptor groups at the other double bond. None of the new derivatives were able to undergo norbornadiene-to-quadricyclane photoisomerization, not even by the use of a photosensitizer; instead, photodegradations of the compounds occurred. Due to the experimental conditions, we cannot rule out that a short-lived QC form of the molecules exists but due to the time resolution of our experiments, we cannot assess this. For allowing the photoswitching event, it seems that the loss of aromaticity should be further decreased from that of the phenanthrene unit.

## 4. Materials and Methods

### 4.1. General Methods

THF used for coupling reactions was distilled over a Na/benzophenone couple or retrieved from an Innovative Technology PURESOLV^TM^ solvent purification system of the model PS-MD-05. UV-Vis spectra were acquired using a Varian Cary 50 Bio spectrophotometer. UV-Vis spectroscopy experiments were performed using a quartz cuvette with a 1 cm path length coupled to a Peltier element for temperature control. Irradiation studies were performed using an ozone-free 450-W Xe lamp equipped with a monochromator, a broad spectrum TLC lamp (365 nm) or a Thorlabs 405 nm LED lamp of the model M405LP1. Thin-layer chromatography (TLC) was carried out using aluminum sheets precoated with silica gel. NMR spectra were acquired using a Bruker 500 MHz instrument with a non-inverse cryo-probe or a Varian 400 MHz instrument using the residual solvent peak as the reference (CDCl_3_: ^1^H 7.26 ppm (CHCl_3_), ^13^C 77.16 ppm. *d8*-Toluene: ^1^H 2.090 ppm (*d7*-toluene)). Mass spectra (HRMS) were acquired on an ESP-MALDI-FT-ICR spectrometer equipped with a 7 T magnet (calibration of the instrument was done with NaTFA cluster ions). Elemental analyses were performed at Department of Chemistry, University of Copenhagen. Purification by flash column chromatography was carried out on silica gel (SiO_2_, 60 Å, 40–63 µm). When referring to petroleum spirit a technical grade with boiling point 40–65 °C was used.

General assignments of ^1^H-NMR resonances: NBD bridgehead C(sp^3^)-H signals are observed in the region 2.3–3.0 ppm when the NBD positions are differently substituted as a pair of signals (each integrating as 1H) with apparent dt multiplicities (in principle, ddd with two similar coupling constants). NBD-CH_2_ protons are observed in the region 3.8–4.9 ppm as two multiplets (each 1H). N(CH_3_)_2_ and OCH_3_ protons are observed as singlets at 3.0 ppm (6H) and 3.8–3.9 ppm (3H), respectively; for compounds **9** and **10**, two singlets are observed (corresponding to two different N(CH_3_)_2_ and OCH_3_ units, respectively). Aromatic protons are seen at or above 6.6 ppm.

### 4.2. Synthesis Protocols

**2-Bromo-3-chloro-1,4-dihydro-1,4-methanonaphthalene (2).** To a solution of *t*-BuOK (4.88 g, 43.5 mmol) in anhydrous THF (100 mL) at −84 °C were added **1** (7.09 g, 49.9 mmol) followed by *n*-BuLi (2.5 M in hexanes, 16.8 mL, 42.0 mmol) over the course of 30 min. The mixture was stirred for 1 h at −41 °C, before it was cooled to −84 °C and *p*-toluenesulfonyl chloride (8.03 g, 42.1 mmol) was added and the mixture was stirred at −84 °C for 30 min. Once again *n*-BuLi (2.5 M in hexanes, 16.8 mL, 42.0 mmol) was added over the course of 30 min, before the mixture was stirred at −41 °C for 1 h. The mixture was cooled to −84 °C and *p*-toluenesulfonyl bromide (9.95 g, 42.3 mmol) was added. The mixture was stirred for 20 min at −84 °C, before it was allowed to reach rt. The reaction mixture was quenched with H_2_O (400 mL), the phases were separated and the aqueous phase was extracted with Et_2_O (3 × 200 mL). The combined organic phases were dried over Na_2_SO_4_, filtered and concentrated in vacuo. The crude mixture was redissolved in hexane, poured through a plug of silica (43–60 µm) and concentrated in vacuo. The mixture was concentrated by a stream of nitrogen affording **2** as a white solid of sufficient purity for further reaction (6.78 g, 54%). ^1^H NMR (400 MHz, CDCl_3_): *δ* = 7.34 (dd, *J* = 5.2, 3.0 Hz, 2H), 7.05 (dd, *J* = 5.2, 3.0 Hz, 2H), 3.92 (m, 1H), 3.86 (m, 1H), 2.74 (dd, *J* = 7.4, 1.8 Hz, 1H), 2.40 (dd, *J* = 7.4, 1.8 Hz, 1H) ppm. ^13^C NMR (100 MHz, CDCl_3_): *δ* = 148.31, 147.86, 144.49, 143.13, 129.32, 125.67, 125.64, 122.19, 66.90, 58.14, 57.14 ppm. HR-MS (APCI^+^ FT-ICR): *m/z* = 254.95769 [M+H^+^], calcd. for [C_11_H_9_^79^BrCl^+^]: *m/z* = 254.95707.

**3-Chloro-1,4-dihydro-1,4-methanonaphthalene-2-carbonitrile (3).** To an argon flushed solution of **2** (6.00 g, 23.5 mmol) in DMF (80 mL) was added CuCN (1.88 g, 21.0 mmol) and the mixture was heated to 105 °C for 15 h. The mixture was allowed to reach rt, before it was poured into saturated aqueous Na_2_CO_3_ (200 mL) and extracted with Et_2_O (3 × 300 mL). The combined organic phases were washed with water (2 × 400 mL) and brine (2 × 400 mL), dried over Na_2_SO_4_, filtered and concentrated in vacuo. The crude mixture was purified by flash column chromatography (30% CH_2_Cl_2_/heptane to 100% CH_2_Cl_2_) affording **3** as an off-white solid (2.20 g, 46%) along with **4** (326 mg, 7%). **Compound 3:** *R_f_* (50% CH_2_Cl_2_/petroleum spirit) = 0.47. IR: 3070, 3051, 3022, 2990, 2950, 2874, 2215, 1589 cm^-1^. ^1^H NMR (400 MHz, CDCl_3_): *δ* = 7.42–7.33 (m, 2H), 7.15–7.03 (m, 2H), 4.18 (m, 1H), 3.96 (m, 1H), 2.72 (dt, *J* = 7.9, 1.7 Hz, 1H), 2.45 (dt, *J* = 7.9, 1.7 Hz, 1H) ppm. ^13^C NMR (100 MHz, CDCl3): *δ* = 164.18, 147.36, 145.79, 126.54, 126.13, 123.15, 122.75, 121.09, 114.12, 68.22, 58.13, 53.63 ppm. HR-MS (ESI^+^ FT-ICR): *m/z* = 202.04186 [M+H+], calcd. for [C_12_H_9_ClN^+^]: *m/z* = 202.04180. **Compound 4:** ^1^H NMR (400 MHz, CDCl_3_): *δ* = 7.42 (dd, *J* = 5.3, 3.1 Hz, 2H), 7.13 (dd, *J* = 5.3, 3.1 Hz, 2H), 4.32 (t, *J* = 1.6 Hz, 2H), 2.70 (dd, *J* = 8.4, 1.6 Hz, 1H), 2.53 (dd, *J* = 8.4, 1.6 Hz, 1H) ppm. ^13^C NMR (126 MHz, CDCl_3_): *δ* = 144.87, 140.47, 126.85, 123.74, 112.84, 70.06, 54.86 ppm. HR-MS (ESI^+^ FT-ICR): *m/z* = 193.07619 [M+H^+^], calcd. for [C_13_H_9_N_2_^+^]: *m/z* = 193.07602. EA (C_13_H_8_N_2_): calcd. C 81.23, H 4.20, N 14.57; found C 80.86, H 4.51, N 15.00.

**3-((4-(Dimethylamino)phenyl)ethynyl)-1,4-dihydro-1,4-methanonaphthalene-2-carbonitrile (7).** To an argon flushed solution of **3** (200 mg, 0.992 mmol) and 4-ethynyl-*N,N*-dimethylaniline (**5**) (156 mg, 1.07 mmol) in THF (20 mL) and Et_3_N (12 mL) were added CuI (18 mg, 0.095 mmol) and Pd(PPh_3_)_2_Cl_2_ (71 mg, 0.10 mmol) and the mixture was stirred overnight. The reaction mixture was poured through a plug of silica (43–60 μm, CH_2_Cl_2_) and concentrated in vacuo. The residue was subjected to flash column chromatography (60% CH_2_Cl_2_/hexane) affording **7** as a yellow solid (238 mg, 77%). *R_f_* (60% CH_2_Cl_2_/hexane) = 0.38. ^1^H NMR (400 MHz, CDCl_3_): *δ* = 7.41–7.33 (m, 2H), 7.38 (d, *J* = 9.1 Hz, 2H), 7.11–7.00 (m, 2H), 6.62 (d, *J* = 9.1 Hz, 2H), 4.16 (m, 1H), 4.11 (m, 1H), 3.01 (s, 6H), 2.59 (dt, *J* = 7.9, 1.7 Hz, 1H), 2.41 (dt, *J* = 7.9, 1.7 Hz, 1H) ppm. ^13^C NMR (100 MHz, CDCl_3_): *δ* = 153.61, 151.06, 147.86, 147.02, 133.67, 126.03, 125.83, 124.45, 123.00, 122.49, 116.73, 111.69, 111.04, 108.22, 82.26, 67.96, 57.02, 53.49, 40.20 ppm. HR-MS (ESI^+^ FT-ICR): *m/z* = 311.1552 [M+H^+^], calcd. for [C_22_H_19_N_2_^+^]: *m/z* = 311.1543.

**Compound 8.** To a solution of ((4-methoxyphenyl)ethynyl)trimethylsilane (230 mg, 1.13 mmol) in THF and MeOH (20 mL, 1:1, *v/v*) was added K_2_CO_3_ (594 mg, 4.30 mmol) and the mixture was stirred at rt for 1 h. The mixture containing **6** was poured through a plug of silica (40–63 μm, CH_2_Cl_2_), almost concentrated in vacuo zand then THF (50 mL) was added and the mixture was concentrated to approx. 10 mL. Then **3** (207 mg, 1.03 mmol) and Et_3_N (10 mL) were added and the mixture was flushed with argon. Pd(PPh_3_)_2_Cl_2_ (85 mg, 0.12 mmol) and CuI (17 mg, 89 μmol) were added and the mixture was stirred for 19 h at rt. The reaction mixture was poured through a plug of silica (40–63 μm, CH_2_Cl_2_) and concentrated in vacuo. The residue was subjected to flash column chromatography (50% CH_2_Cl_2_/heptane to 70% CH_2_Cl_2_/heptane) affording **8** as a slightly brown semicrystalline oil (222 mg, 73%). *R_f_* (70% CH_2_Cl_2_/heptane) = 0.45. ^1^H NMR (500 MHz, CDCl_3_): *δ* = 7.45 (d, *J* = 8.9 Hz, 2H), 7.41–7.37 (m, 1H), 7.37–7.34 (m, 1H), 7.09–7.04 (m, 2H), 6.87 (d, *J* = 8.9 Hz, 2H), 4.17 (m, 1H), 4.12 (m, 1H), 3.83 (s, 3H), 2.61 (dt, *J* = 8.0, 1.8 Hz, 1H), 2.42 (dt, *J* = 8.0, 1.8 Hz, 1H) ppm. ^13^C NMR (126 MHz, CDCl_3_): *δ* = 160.91, 153.27, 147.60, 146.87, 133.85, 126.80, 126.14, 125.97, 123.07, 122.67, 116.26, 114.33, 114.01, 108.70, 82.10, 68.26, 56.93, 55.51, 53.65 ppm. HR-MS (ESI^+^ FT-ICR): *m/z* = 298.12310 [M+H^+^], calcd. for [C_21_H_16_NO^+^]: *m/z* = 298.12264. EA (C_21_H_15_NO): calcd. C 84.82, H 5.08, N 4.71; found C 84.29, H 5.20, N 4.85.

**Compound 9.** To an argon flushed solution of **3** (221 mg, 1.10 mmol) and 4-ethynyl-*N,N*-dimethylaniline (**5**) (246 mg, 1.69 mmol) in THF (20 mL) and Et_3_N (12 mL) were added CuI (20 mg, 0.11 mmol) and Pd(PPh_3_)_2_Cl_2_ (77 mg, 0.11 mmol) and the mixture was stirred for 2 h. More 4-ethynyl-*N,N*-dimethylaniline (181 mg, 1.25 mmol) was added and the mixture was stirred overnight. The reaction mixture was poured through a plug of silica (43–60 μm, CH_2_Cl_2_) and concentrated in vacuo. The residue was subjected to flash column chromatography (80% CH_2_Cl_2_/hexane to 100% CH_2_Cl_2_) affording **9** as a red solid (363 mg, 73%). *R_f_* (80% CH_2_Cl_2_/heptane) = 0.28. IR = 3047, 2986, 2941, 2896, 2862, 2804, 2192, 2208sh, 2177sh, 1606, 1553, 1557sh, 1517 cm^−1^. ^1^H NMR (400 MHz, CDCl_3_): *δ* = 8.04 (d, *J* = 9.1 Hz, 2H), 7.55 (d, *J* = 9.0 Hz, 2H), 7.42–7.33 (m, 2H), 7.15 (s, 1H), 7.09–6.98 (m, 2H), 6.70 (d, *J* = 9.1 Hz, 2H), 6.69 (d, *J* = 9.0 Hz, 2H), 4.50 (m, 1H), 4.18 (m, 1H), 3.04 (s, 6H), 2.99 (s, 6H), 2.54 (dt, *J* = 7.8, 1.7 Hz, 1H), 2.35 (dd, *J* = 7.8, 1.7 Hz, 1H) ppm. ^13^C NMR (100 MHz, CDCl_3_): *δ* = 168.37, 151.12, 150.35, 149.32, 147.32, 136.99, 132.67, 131.80, 125.60, 125.49, 124.03, 122.45, 122.07, 118.19, 115.18, 112.04, 111.59, 111.34, 110.32, 99.08, 84.78, 65.77, 55.04, 53.25, 40.34, 40.24 ppm. HR-MS (ESI^+^ FT-ICR): *m/z* = 456.2438 [M+H^+^], calcd. for [C_32_H_30_N_3_^+^]: *m/z* = 456.2434.

**Compound 10.** To a solution of ((4-methoxyphenyl)ethynyl)trimethylsilane (649 mg, 3.18 mmol) in THF/MeOH (20 mL, 1:1, *v/v*) was added K_2_CO_3_ (1.56 g, 11.3 mmol) and the mixture was stirred at rt for 2 h. The mixture was poured through a plug of silica (43–60 μm, CH_2_Cl_2_) and concentrated in vacuo to provide **6**. THF (20 mL), Et_3_N (12 mL) and **3** (180 mg, 0.893 μmol) were added and the mixture was flushed with argon. CuI (17 mg, 0.089 mmol) and Pd(PPh_3_)_2_Cl_2_ (69 mg, 0.098 mmol) were added and the mixture was stirred at rt for 23 h. The reaction mixture was poured through a plug of silica (43–60 μm, CH_2_Cl_2_) and concentrated in vacuo. The residue was subjected to flash column chromatography (70% CH_2_Cl_2_/hexane to 90% CH_2_Cl_2_/hexane) affording **10** as a yellow solid (303 mg, 79%). *R_f_* (80% CH_2_Cl_2_/heptane) = 0.41. ^1^H NMR (400 MHz, CDCl_3_): *δ* = 8.03 (d, *J* = 8.9 Hz, 2H), 7.59 (d, *J* = 8.9 Hz, 2H), 7.43–7.35 (m, 2H), 7.19 (s, 1H), 7.11–7.00 (m, 2H), 6.94 (d, *J* = 8.9 Hz, 2H), 6.90 (d, *J* = 8.9 Hz, 2H), 4.48 (m, 1H), 4.21 (m, 1H), 3.86 (s, 3H), 3.83 (s, 3H), 2.56 (dt, *J* = 7.9, 1.7 Hz, 1H), 2.38 (dt, *J* = 7.9, 1.7 Hz, 1H) ppm. ^13^C NMR (100 MHz, CDCl_3_): *δ* = 167.72, 160.87, 160.14, 148.98, 147.04, 136.88, 133.20, 131.74, 128.52, 125.79, 125.68, 122.48, 122.31, 117.79, 117.62, 115.13, 114.29, 114.06, 113.84, 98.10, 84.59, 66.14, 55.53, 55.46, 55.20, 53.21 ppm. HR-MS (ESI^+^ FT-ICR): *m/z* = 430.1811 [M+H^+^], calcd. for [C_30_H_24_NO_2_^+^]: *m/z* = 430.1802. EA (C_30_H_23_NO_2_): calcd. C 83.89, H 5.40, N 3.26; found C 83.34, H 5.40, N 3.35.

**2-Bromo-3-chloro-1,4-dihydro-1,4-methanoanthracene (12).** A solution of potassium *tert*-butoxide (4.53 g, 40.4 mmol) in anhydrous THF (180 mL) was cooled to −78 °C, before **11** (9.32 g, 48.5 mmol) was added followed by *n*-BuLi (16.16 mL, 2.5 M, 40.41 mmol). The temperature was elevated to −41 °C and the mixture was stirred for 1 h. The reaction mixture was cooled to −78 °C before *p*-toluenesulfonyl chloride (7.71 g, 40.4 mmol) was added. The mixture was stirred for 30 min at −78 °C; then *n*-BuLi (16.16 mL, 2.5 M, 40.41 mmol) was added, the temperature was elevated to −41 °C and the mixture was stirred for 1 h. The mixture was once again cooled to −78 °C before *p*-toluenesulfonyl bromide (9.63 g, 41.0 mmol) was added and the mixture was stirred for 15 min. The reaction mixture was heated to rt using a water bath, poured into water (300 mL) and extracted with diethyl ether (3 × 200 mL). The combined organic phases were washed with water (2 × 400 mL) and brine (400 mL), dried over Na_2_SO_4_, filtered and concentrated in vacuo. Flash column chromatography (petroleum spirit) furnished **12** (estimated yield of ca. 15%) and **11** as a 41:59 mixture (based on NMR integration) (3.40 g). The mixture of **12** and **11** was used in the next step without further purification.

**3-Chloro-1,4-dihydro-1,4-methanoanthracene-2-carbonitrile (13).** To an argon-flushed solution of the mixture of **11** and **12** (3.14 g) in DMF (40 mL) was added CuCN (435 mg, 4.86 mmol) and the mixture was heated to 100 °C and stirred for 10h. The mixture was allowed to cool to rt before it was poured into saturated aqueous sodium carbonate (200 mL) and extracted with diethyl ether (3 × 150 mL). The combined organic phases were washed with water (3 × 200 mL) and brine (200 mL), dried over Na_2_SO_4_, filtered and concentrated *in vacuo*. Flash column chromatography (30% CH_2_Cl_2_/heptane to 50% CH_2_Cl_2_/heptane) afforded **13** as a white solid (415 mg, 31%). IR = 3076, 3063, 3048, 2998, 2953, 2217, 1591, 1509sh, 1506 cm^−1^. ^1^H NMR (400 MHz, CDCl_3_): *δ* = 7.80–7.73 (m, 4H), 7.53–7.43 (m, 2H), 4.27 (m, 1H), 4.07 (m, 1H), 2.76 (dt, *J* = 8.3, 1.7 Hz, 1H), 2.45 (dt, *J* = 8.3, 1.7 Hz, 1H) ppm. ^13^C NMR (100 MHz, CDCl_3_): *δ* = 162.47, 143.13, 141.78, 132.56, 132.30, 128.18, 128.11, 126.59, 126.47, 121.97, 121.32, 120.40, 113.96, 64.72, 57.33, 52.83 ppm. HR-MS (ESI^+^ FT-ICR): *m/z* = 252.05767 [M+H^+^], calcd. for [C_16_H_11_ClN^+^]: *m/z* = 252.05745. EA (C_16_H_10_ClN): calcd. C 76.35, H 4.00, N 5.56; found C 76.21, H 4.12, N 5.71.

**3-((4-(Dimethylamino)phenyl)ethynyl)-1,4-dihydro-1,4-methanoanthracene-2-carbonitrile (14).** To an argon flushed solution of **13** (151 mg, 0.600 mmol) and 4-ethynyl-*N,N*-dimethylaniline (**5**) (95 mg, 0.65 mmol) in THF/Et_3_N (20 mL, 1:1, *v/v*) were added CuI (12 mg, 63 μmol) and Pd(PPh_3_)_2_Cl_2_ (44 mg, 63 μmol) and the mixture was stirred at rt for 4 h. The reaction mixture was poured through a plug of silica (43–60 μm, CH_2_Cl_2_) and concentrated in vacuo. The crude mixture was purified by flash column chromatography (50% CH_2_Cl_2_/heptane) affording **14** as a yellow solid (186 mg, 86%). *R_f_* (50% CH_2_Cl_2_/heptane) = 0.13. ^1^H NMR (400 MHz, CDCl_3_): *δ* = 7.80–7.69 (m, 4H), 7.49–7.39 (m, 2H), 7.38 (d, *J* = 9.1 Hz, 2H), 6.61 (d, *J* = 9.1 Hz, 2H), 4.25 (m, 1H), 4.21 (m, 1H), 3.00 (s, 6H), 2.64 (dt, *J* = 8.3, 1.7 Hz, 1H), 2.40 (dd, *J* = 8.3, 1.7 Hz, 1H) ppm. ^13^C NMR (100 MHz, CDCl_3_): *δ* = 152.19, 151.09, 143.97, 143.25, 133.74, 132.53, 132.40, 128.08, 128.01, 126.16, 126.08, 123.45, 121.55, 120.86, 116.54, 111.68, 110.66, 108.13, 82.18, 64.52, 56.17, 52.65, 40.19 ppm. HR-MS (ESI^+^ FT-ICR): *m/z* = 361.17004 [M+H^+^], calcd. for [C_26_H_21_N_2_^+^]: *m/z* = 361.16993.

**3-((4-Methoxyphenyl)ethynyl)-1,4-dihydro-1,4-methanoanthracene-2-carbonitrile (15).** To a solution of ((4-methoxyphenyl)ethynyl)trimethylsilane (97 mg, 0.47 mmol) in THF (5 mL) and MeOH (5 mL) was added K_2_CO_3_ (284 mg, 2.05 mmol) and the mixture was stirred at rt for 1 h. The mixture was poured through a plug of silica (40–63 μm, CH_2_Cl_2_), almost concentrated in vacuo and then THF (50 mL) was added and the mixture containing **6** was concentrated to approx. 10 mL. Then **13** (110 mg, 0.437 mmol) and Et_3_N (10 mL) were added and the mixture was flushed with argon. Pd(PPh_3_)_2_Cl_2_ (35 mg, 50 μmol) and CuI (10 mg, 53 μmol) were added and the mixture was stirred for 22h at rt. The reaction mixture was poured through a plug of silica (40–63 μm, CH_2_Cl_2_) and concentrated in vacuo. The crude material was subjected to flash column chromatography (50% CH_2_Cl_2_/heptane to 70% CH_2_Cl_2_/heptane) affording **15** as an off-white solid (80 mg, 53%). *R_f_* (70% CH_2_Cl_2_/heptane) = 0.47. ^1^H NMR (500 MHz, CDCl_3_): *δ* = 7.79–7.72 (m, 4H), 7.48–7.42 (m, 4H), 6.87 (d, *J* = 8.9 Hz, 2H), 4.27 (m, 1H), 4.23 (m, 1H), 3.83 (s, 3H), 2.66 (dt, *J* = 8.4, 1.6 Hz, 1H), 2.43 (dt, *J* = 8.4, 1.5 Hz, 1H) ppm. ^13^C NMR (126 MHz, CDCl_3_): *δ* = 160.94, 151.82, 143.61, 142.98, 133.90, 132.51, 132.40, 128.11, 128.02, 126.26, 126.20, 125.74, 121.65, 121.11, 116.06, 114.32, 113.91, 108.26, 82.00, 64.76, 56.04, 55.49, 52.79 ppm. HR-MS (ESI^+^ FT-ICR): *m/z* = 348.13845 [M+H^+^], calcd. for [C_25_H_18_NO^+^]: *m/z* = 348.13829.

**2,2′′-Bis(dibromomethyl)-1,1′′-biphenyl (17).** A solution of 2,22′′-dimethyl-1,11′′-biphenyl (**16**) (13.5 mL, 73.3 mmol), NBS (58.10 g, 326.4 mmol) and Luperox^®^ A75, Benzoyl peroxide (1.35 g, 1.01 g benzoyl peroxide, 4.17 mmol) in benzene (500 mL) was heated to reflux point while irradiated with a 500 W halogen lamp for 18 h. The mixture was allowed to cool to rt, poured through a plug of silica (43–60 μm) and concentrated in vacuo. The crude mixture was recrystallized from CH_2_Cl_2_/heptane affording **17** as an off-white solid (30.10 g, 83%). The reaction was also performed on 5.49 mmol scale giving a yield of 76%. ^1^H NMR (500 MHz, CDCl_3_): d = 8.13 (dd, *J* = 7.8, 1.3 Hz, 2H), 7.58 (td, *J* = 7.8, 1.4 Hz, 2H), 7.41 (td, *J* = 7.8, 1.3 Hz, 2H), 7.12 (dd, *J* = 7.8, 1.4 Hz, 2H), 6.29 (s, 2H) ppm. ^13^C NMR (126 MHz, CDCl3): *δ* = 140.46, 133.62, 130.56, 129.96, 129.74, 129.05, 38.55 ppm. 

**9,10-Dibromophenanthrene (18).** To a solution of **17** (29.59 g, 59.44 mmol) in anhydrous DMF (450 mL) at 0 °C was added potassium *tert*-butoxide (21.65 g, 192.9 mmol). The mixture was stirred at 0 °C for 1h, before the mixture was quenched slowly with 6 M HCl (500 mL). The precipitate was filtered, washed with water (1.5 L) and MeOH (200 mL) and dried in vacuo affording **18** as a yellow solid (18.10 g, 91%). The reaction was also done on 3.02 mmol scale giving a yield of 79%. ^1^H NMR (500 MHz, CDCl_3_): *δ* = 8.63 (dd, *J* = 8.2, 1.4 Hz, 2H), 8.47 (dd, *J* = 8.1, 1.5 Hz, 2H), 7.70 (ddd, *J* = 8.2, 7.0, 1.5 Hz, 2H), 7.66 (ddd, *J* = 8.1, 7.0, 1.4 Hz, 2H). ^13^C NMR (126 MHz, CDCl_3_): *δ* = 131.27, 130.36, 129.68, 128.18, 127.76, 126.24, 122.84 ppm. HR-MS (APCI^+^ FT-ICR): *m/z* = 334.90806 [M+H^+^], calcd. for [C_14_H_9_^79^Br_2_^+^]: *m/z* = 334.90655.

**1,4-Dihydro-1,4-methanotriphenylene (19).** To solution of **18** (17.84 g, 53.09 mmol) and cyclopentadiene (4.8 mL, 57 mmol) in anhydrous toluene (600 mL) at 0 °C was added *n*-BuLi (23.4 mL, 2.5 M in hexane, 58.5 mmol). The mixture was allowed to reach rt slowly overnight, before it was poured into water (1 L) and extracted with Et_2_O (3 × 500 mL). The combined organic phases were dried over Na_2_SO_4_, filtered and concentrated in vacuo. The residue was passed through a plug of silica (43–60 µm, 50% CH_2_Cl_2_/heptane) and concentrated in vacuo. Flash column chromatography (heptane to 5% CH_2_Cl_2_/heptane) afforded **19** as a white solid (5.80 g, 45%). ^1^H NMR (500 MHz, CDCl_3_): *δ* = 8.73 (d, *J* = 8.1 Hz, 2H), 8.06 (dd, *J* = 8.0, 1.4 Hz, 2H), 7.64 (ddd, *J* = 8.0, 6.9, 1.3 Hz, 2H), 7.59 (ddd, *J* = 8.1, 6.9, 1.4 Hz, 2H), 7.03 (d, *J* = 1.7 Hz, 2H), 4.64 (m, 2H), 2.56 (dt, *J* = 6.6, 1.7 Hz, 1H), 2.47 (dt, *J* = 6.6, 1.7 Hz, 1H) ppm. ^13^C NMR (126 MHz, CDCl_3_): *δ* = 147.89, 143.78, 129.00, 128.73, 126.51, 125.26, 123.55, 123.54, 72.46, 49.22 ppm. HR-MS (APCI^+^ FT-ICR): *m/z* = 243.11693 [M+H^+^], calcd. for [C_14_H_19_^+^]: *m/z* = 243.11683.

**2-Bromo-3-chloro-1,4-dihydro-1,4-methanotriphenylene (20).** To a solution of *t*-BuOK (1.93 g, 17.2 mmol) in THF (300 mL) at −78 °C was added **19** (5.13 g, 21.2 mmol). The cooling bath was removed until everything was in solution, before the mixture was cooled once again to −78 °C. *n*-BuLi (6.85 mL, 2.5 M in hexanes, 17.1 mmol) was added over the course of 45 min resulting in a dark red/brown color. The temperature was elevated to −41 °C and the mixture was stirred for 1 h. The mixture was cooled to −78 °C and *p*-toluenesulfonyl chloride (3.26 g, 17.1 mmol) was added. The reaction mixture was stirred for 40 min, before *n*-BuLi (6.85 mL, 2.5 M in hexanes, 17.1 mmol) was added over the course of 40 min. The temperature was elevated to −41 °C and the mixture was stirred for 1 h. The mixture was cooled to −78 °C, before *p*-toluenesulfonyl bromide (4.04 g, 17.2 mmol) was added and the mixture was stirred for 15 min at −78 °C. The mixture was heated to ambient temperature using a room tempered water bath, before water (150 mL) was added. The remaining THF was removed in vacuo and the resulting mixture was extracted with CH_2_Cl_2_ (3 × 300 mL). The combined organic phases were washed with brine (300 mL), dried over MgSO_4_, filtered and concentrated *in vacuo*. Flash column chromatography (heptane to 5% CH_2_Cl_2_/heptane) furnished **19** and **20** (estimated yield of 32%) as a 61:39 mixture (based on NMR integration) (4.03 g). The mixture of **19** and **20** was used in the next step without further purification.

**3-Chloro-1,4-dihydro-1,4-methanotriphenylene-2-carbonitrile (21).** To an argon-flushed solution of the mixture of **19** and **20** (3.93 g) in DMF (50 mL) was added CuCN (406 mg, 4.53 mmol) and the mixture was heated to 105 °C and stirred for 7 h. The mixture was allowed to cool to rt before it was poured into saturated aqueous sodium carbonate (300 mL) and extracted with diethyl ether (6 × 100 mL). The combined organic phases were washed with water (4 × 250 mL) and brine (2 × 250 mL), dried over Na_2_SO_4_, filtered and concentrated in vacuo. Flash column chromatography (30% CH_2_Cl_2_/heptane to 70% CH_2_Cl_2_/heptane, loaded in 1:4 CH_2_Cl_2_/CS_2_) afforded **21** as a white solid (402 mg, 25%). ^1^H NMR (500 MHz, CDCl_3_): *δ* = 8.79–8.72 (m, 2H), 8.08–8.00 (m, 2H), 7.73–7.64 (m, 4H), 4.89 (m, 1H), 4.72 (m, 1H), 3.00 (dt, *J* = 7.5, 1.8 Hz, 1H), 2.72 (dt, *J* = 7.5, 1.8 Hz, 1H) ppm. ^13^C NMR (126 MHz, CDCl_3_): *δ* = 165.75, 145.27, 142.82, 129.58, 129.54, 128.30, 128.12, 127.36, 127.27, 126.82, 126.59, 123.81, 123.72, 123.64, 123.45, 121.67, 114.24, 70.39, 57.32, 52.80 ppm. HR-MS (ESI^+^ FT-ICR): *m/z* = 302.07324 [M+H^+^], calcd. for [C_20_H_13_ClN^+^]: *m/z* = 302.07310. 

**3-((4-(Dimethylamino)phenyl)ethynyl)-1,4-dihydro-1,4-methanotriphenylene-2-carbonitrile (22).** To a solution of *N,N*-dimethyl-4-((trimethylsilyl)ethynyl)aniline (92 mg, 0.42 mmol) in THF/MeOH (20 mL, 1:1, v/v) was added K_2_CO_3_ (202 mg, 1.46 mmol) and the mixture was stirred for 1 h. The mixture was poured through a plug of silica (43–60 µm, CH_2_Cl_2_), almost concentrated in vacuo; then THF (50 mL) was added and the mixture containing **5** was concentrated to approx. 10 mL. The **21** (106 mg, 351 µmol) and Et_3_N (10 mL) were added and the mixture was flushed with argon. Pd(PPh_3_)_2_Cl_2_ (26 mg, 37 µmol) and CuI (6 mg, 0.03 mmol) were added before the mixture was stirred for 18 h. The mixture was poured through a plug of silica (43–60 µm, CH_2_Cl_2_) and concentrated in vacuo. The residue was purified by flash column chromatography (50–70% CH_2_Cl_2_/heptanes) affording **22** as a yellow solid (99 mg, 69%). ^1^H NMR (500 MHz, CDCl_3_): *δ* = 8.76–8.71 (m, 2H), 8.14–8.05 (m, 2H), 7.72–7.60 (m, 4H), 7.35 (d, *J* = 9.0 Hz, 2H), 6.61 (d, *J* = 9.0 Hz, 2H), 4.88 (m, 1H), 4.85 (m, 1H), 3.00 (s, 6H), 2.86 (dt, *J* = 7.5, 1.6 Hz, 1H), 2.65 (dt, *J* = 7.5, 1.6 Hz, 1H) ppm. ^13^C NMR (126 MHz, CDCl_3_): *δ* = 154.80, 151.08, 145.16, 143.85, 133.61, 129.44, 128.62, 128.30, 127.16, 127.05, 126.40, 126.22, 125.11, 123.73, 123.72, 123.70, 123.62, 116.85, 112.19, 108.34, 82.41, 69.91, 56.27, 52.64, 40.20 ppm. HR-MS (ESI^+^ FT-ICR): *m/z* = 411.18508 [M+H^+^], calcd. for [C_30_H_23_N_2_^+^]: *m/z* = 411.18558.

**3-((4-Methoxyphenyl)ethynyl)-1,4-dihydro-1,4-methanotriphenylene-2-carbonitrile (23).** To a solution of ((4-methoxyphenyl)ethynyl)trimethylsilane (118 mg, 557 µmol) in THF/MeOH (20 mL, 1:1, *v/v*) was added K_2_CO_3_ (213 mg, 1.54 mmol) and the mixture was stirred for 1 h. The mixture was poured through a plug of silica (43–60 µm, CH_2_Cl_2_), almost concentrated in vacuo and then THF (50 mL) was added and the mixture containing **6** was concentrated to approx. 10 mL. Then **21** (153 mg, 507 µmol) and Et_3_N (10 mL) were added and the mixture was flushed with argon. Pd(PPh_3_)_2_Cl_2_ (37 mg, 52 µmol) and CuI (89 µmol) were added before the mixture was stirred for 17 h. The mixture was poured through a plug of silica (43–60 µm, CH_2_Cl_2_) and concentrated in vacuo. The residue was purified by flash column chromatography (50–60% CH_2_Cl_2_/heptanes) affording **23** as a slightly yellow solid (143 mg, 71%). *R_f_* (60% CH_2_Cl_2_/heptanes) = 0.25. ^1^H NMR (500 MHz, CDCl_3_): *δ* = 8.77–8.71 (m, 2H), 8.14–8.05 (m, 2H), 7.73–7.61 (m, 4H), 7.42 (d, *J* = 8.9 Hz, 2H), 6.86 (d, *J* = 8.9 Hz, 2H), 4.90 (m, 1H), 4.87 (m, 1H), 3.82 (s, 3H), 2.88 (dt, *J* = 7.5, 1.6 Hz, 1H), 2.68 (dt, *J* = 7.5, 1.6 Hz, 1H) ppm. ^13^C NMR (126 MHz, CDCl_3_): *δ* = 160.91, 154.48, 144.95, 143.80, 133.78, 129.49, 129.47, 128.57, 128.29, 127.52, 127.24, 127.11, 126.52, 126.34, 123.74, 123.70, 123.66, 123.65, 116.39, 114.32, 114.07, 109.83, 82.21, 70.22, 56.15, 55.51, 52.81 ppm. HR-MS (ESI^+^ FT-ICR): *m/z* = 398.15389 [M+H^+^], calcd. for [C_29_H_20_NO^+^]: *m/z* = 398.15394.

## Figures and Tables

**Table 1:** Longest-wavelength absorption maxima (*λ_max_*/nm) and molar absorptivities (*ε*/10^3^ M^−1^cm^−1^) in brackets.

Compound	*λ_max_*/nm (*ε*/10^3^ M^−1^cm^−1^)
**7**	399 (32.4)
**8**	355 (20.4)
**9**	440 (32.8)
**10**	378 (25.9)
**14**	400 (33.4)
**15**	356 (22.6)
**22**	410 (24.3)
**23**	336 (20.8)

## Supplementary Materials

NMR Spectra of all new compounds. Details on photosensitizer experiments.
