# Supplementary material for: Donor-Acceptor Substituted Benzo-, Naphtho- and Phenanthro-Fused Norbornadienes
Source: Molecules. 2020 Jan 13;25(2):322. doi: 10.3390/molecules25020322 (PMC7024342; doi:10.3390/molecules25020322)
Supplement: Supplementary file 1 [file molecules-25-00322-s001.pdf]

Donor-Acceptor Substituted Benzo-, Naphtho- and Phenanthro-fused Norbornadienes

Mads Mansø [1,2], Lorette Fernandez[2], Zhihang Wang[2], Kasper Moth-Poulsen[2] and Mogens Brøndsted Nielsen[1,*]

[1] Department of Chemistry, University of Copenhagen, Universitetsparken 5, DK-2100 Copenhagen Ø, Denmark
[2] Department of Chemistry and Chemical Engineering, Chalmers University of Technology, Kemivägen 10, 412 96 Gothenburg, Sweden
* Correspondence: mbn@chem.ku.dk

SUPPORTING INFORMATION

Contents

NMR Spectra **page S2**

Photosensitizer Experiments **page S47**

NMR spectra

Compound 2

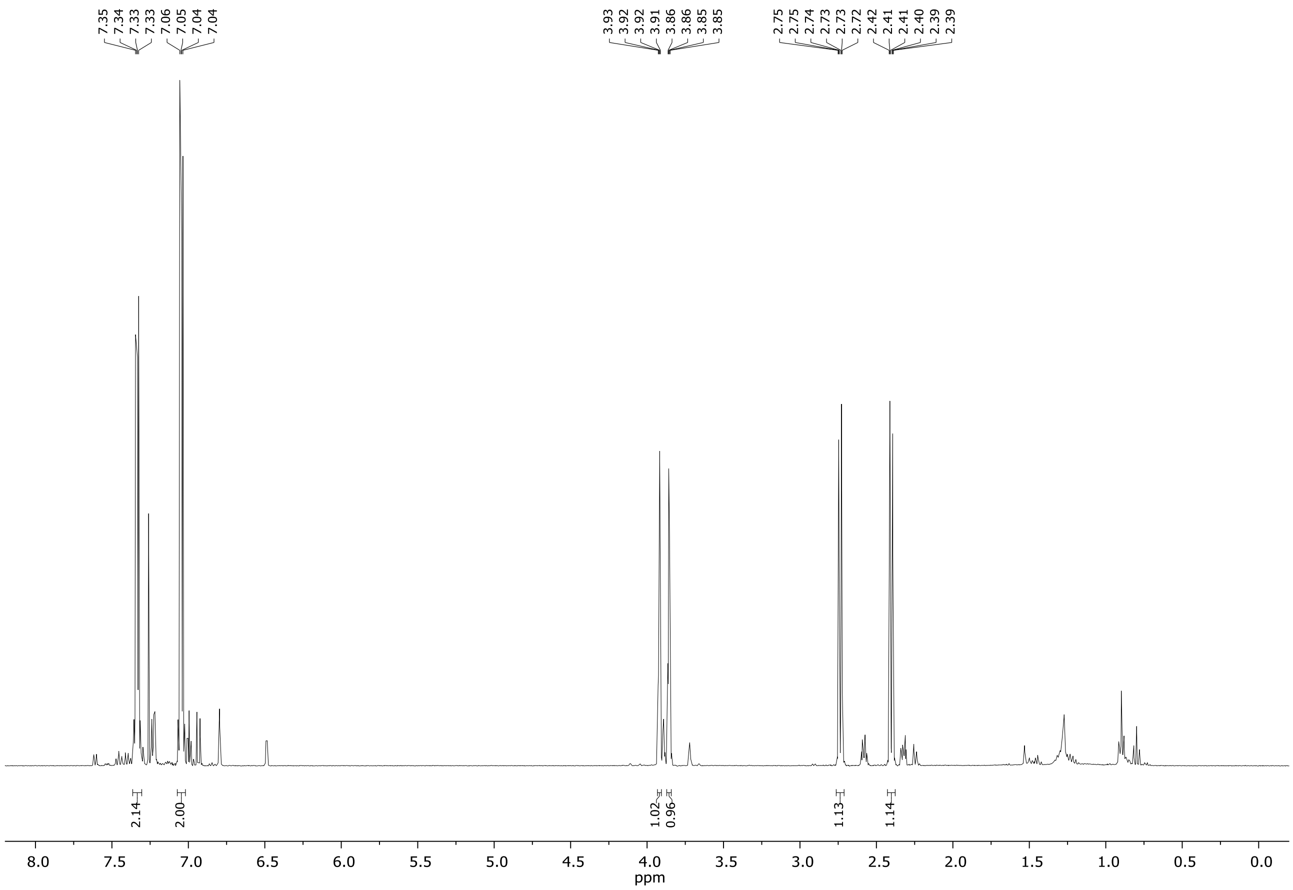

Supplementary Figure 1: ^{1}H NMR (400 MHz) spectrum of **2** in $CDCl_3$.

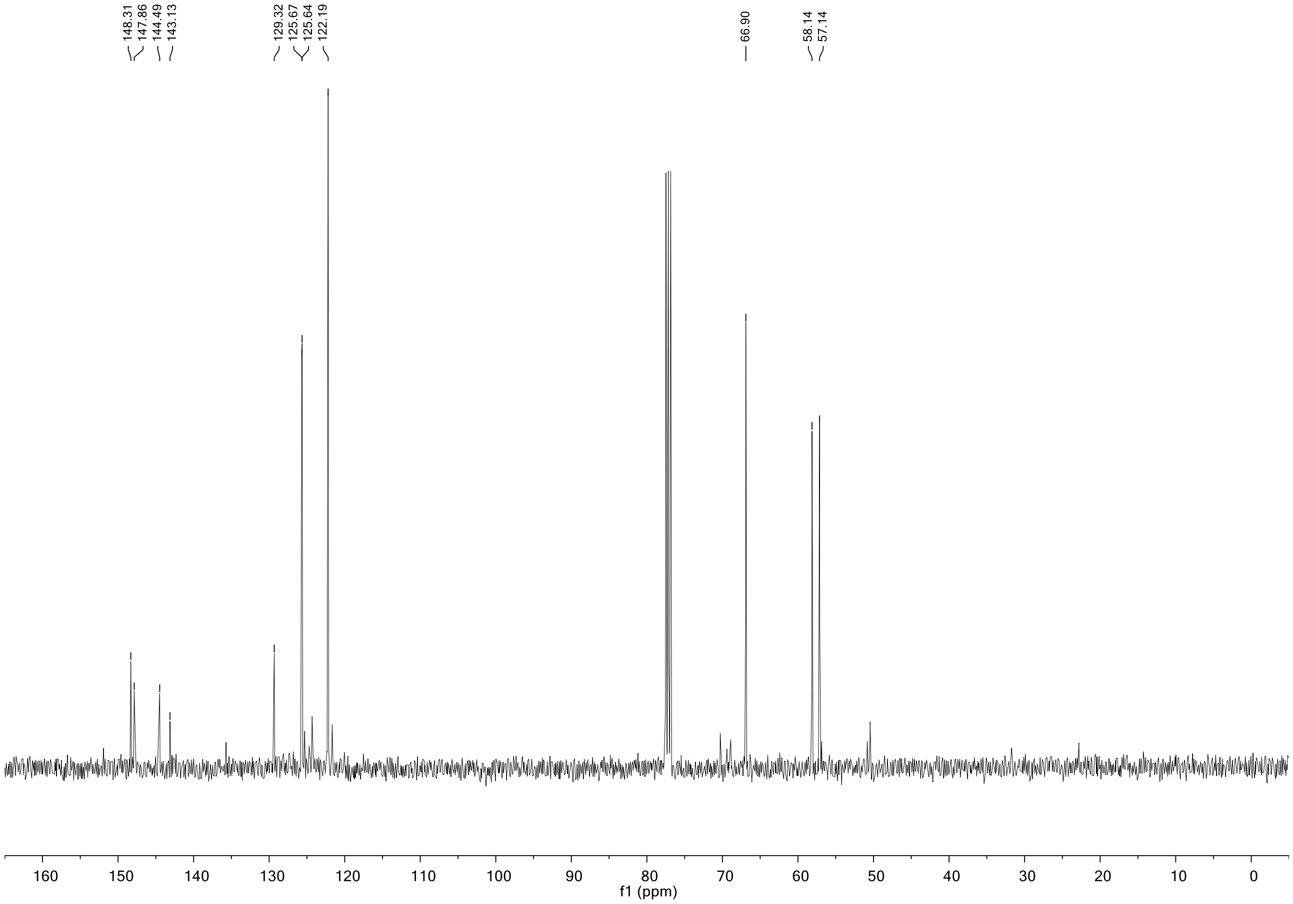

Supplementary Figure 2: ^{13}C NMR (100 MHz) of **2** in $CDCl_3$.

Compound 3

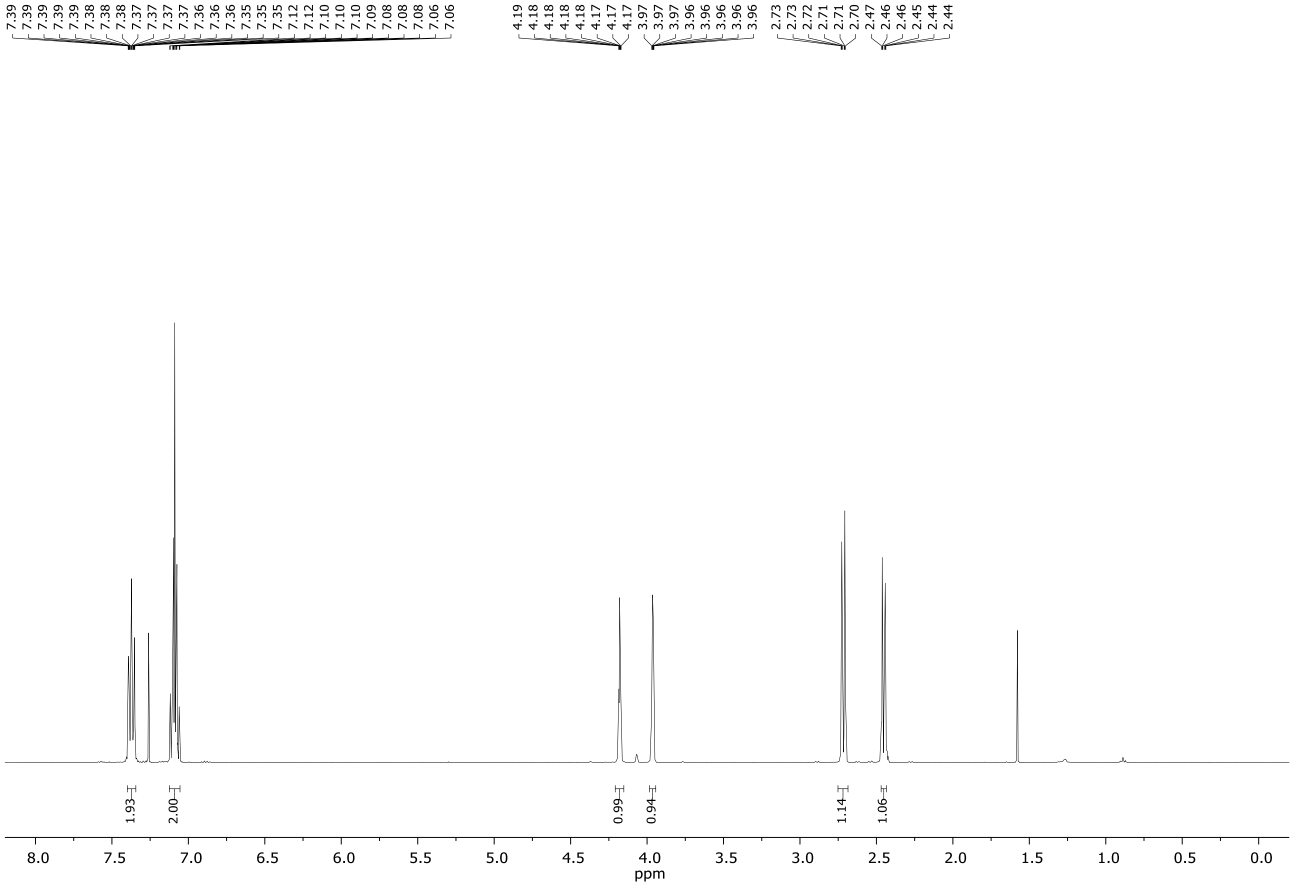

Supplementary Figure 3: ^{1}H NMR (400 MHz) of **3** in $CDCl_3$.

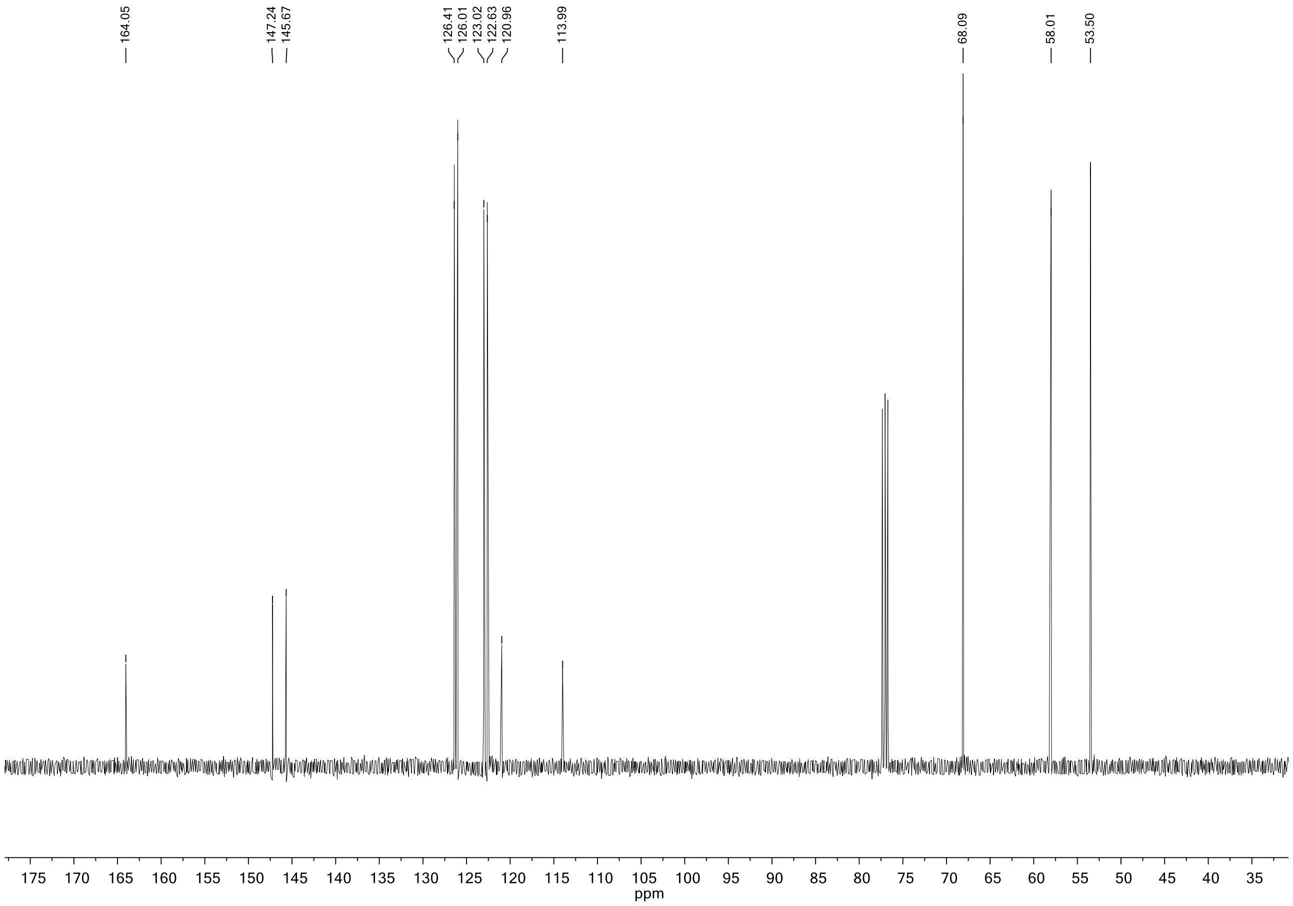

Supplementary Figure 4: ^{13}C NMR (100 MHz) of **3** in $CDCl_3$.

Compound 4

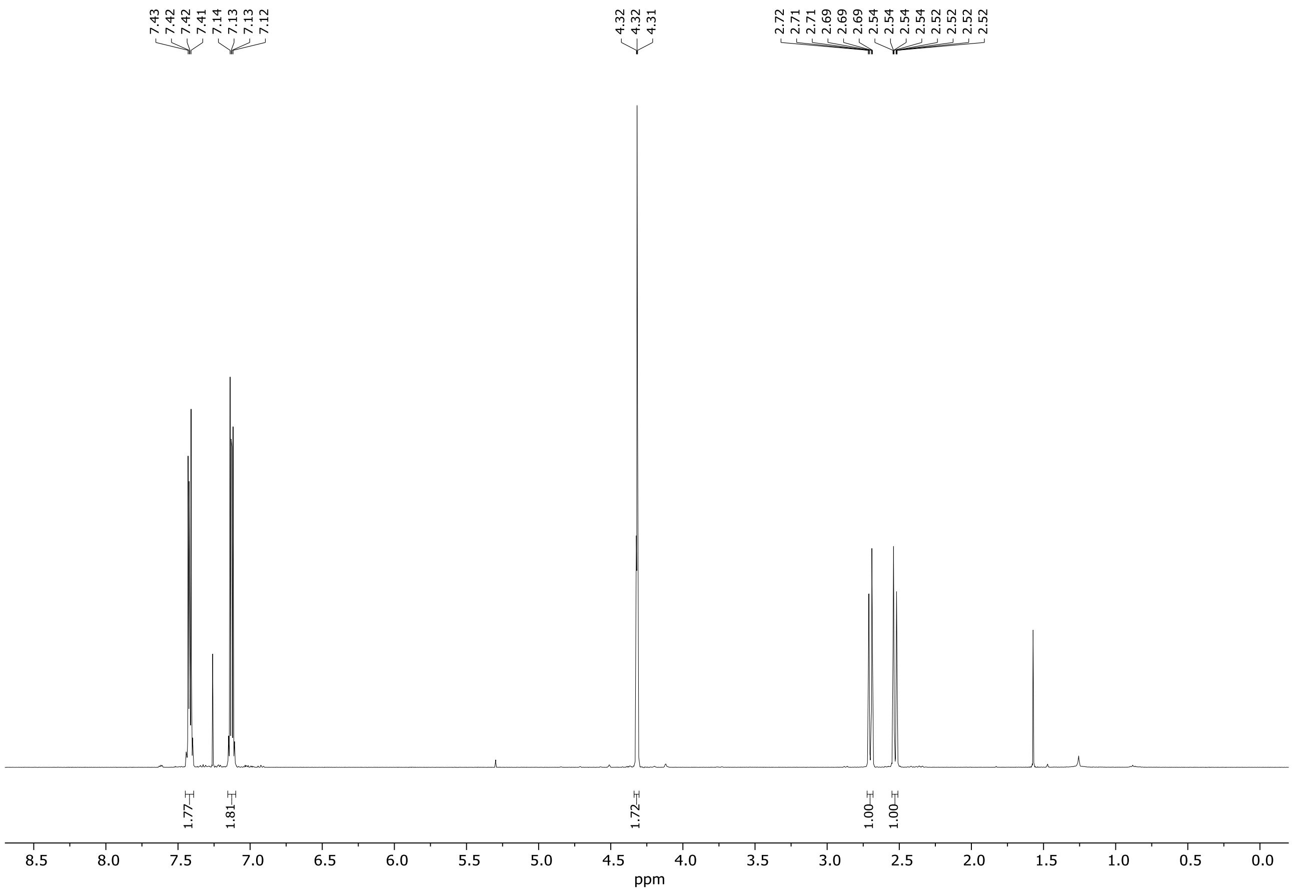

Supplementary Figure 5: ^{1}H NMR (400 MHz) of **4** in $CDCl_3$.

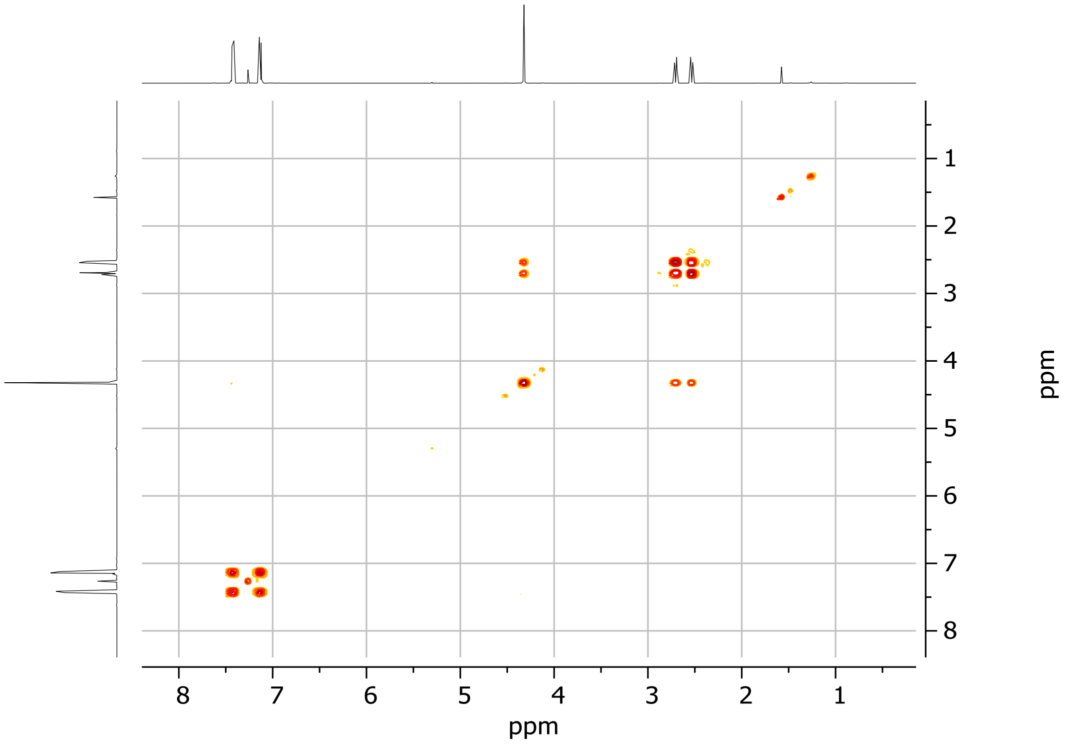

Supplementary Figure 6: COSY NMR (400 MHz) of **4** in $CDCl_3$.

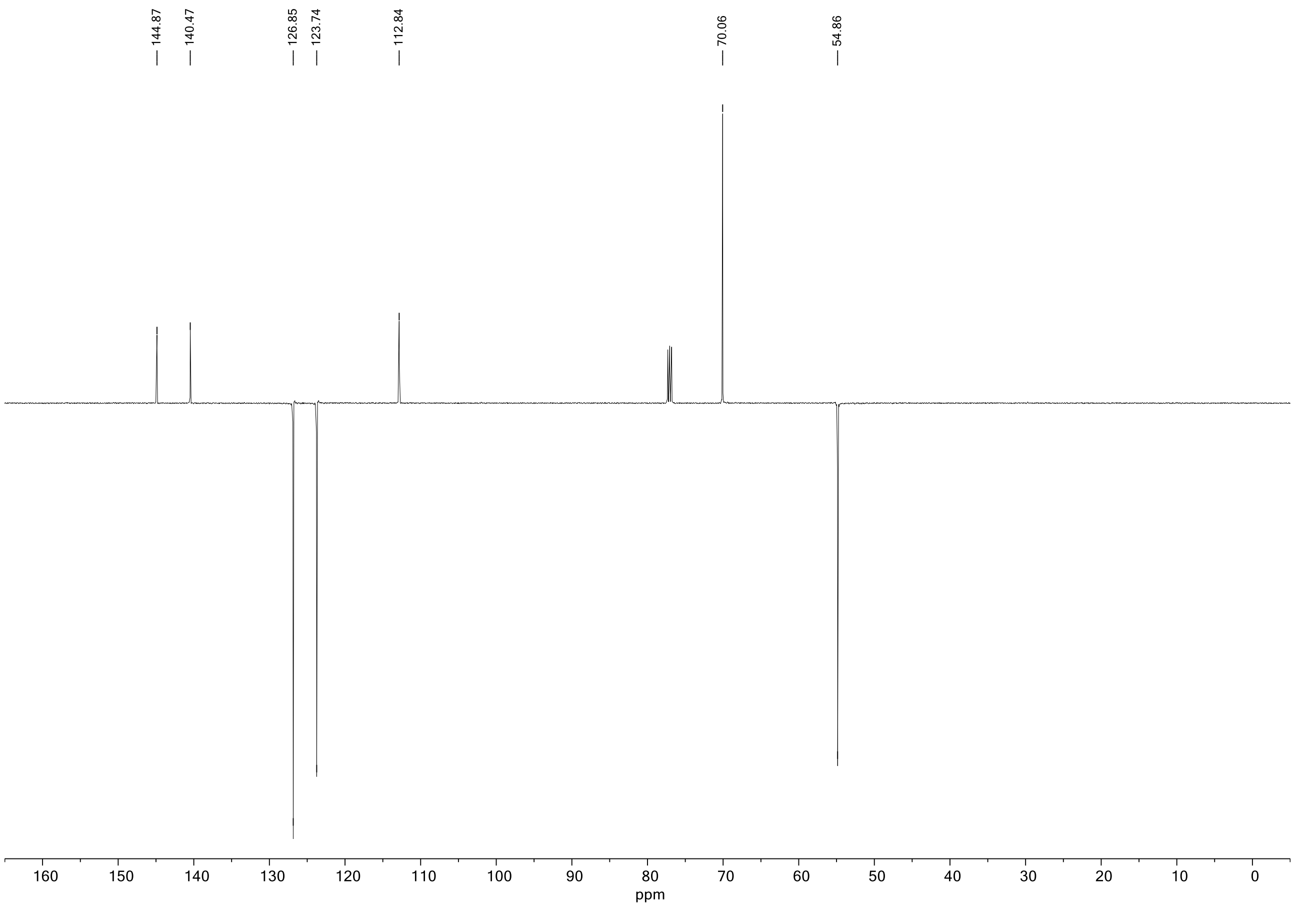

Supplementary Figure 7: ^{13}C NMR (126 MHz) of **4** in $CDCl_3$.

Compound 7

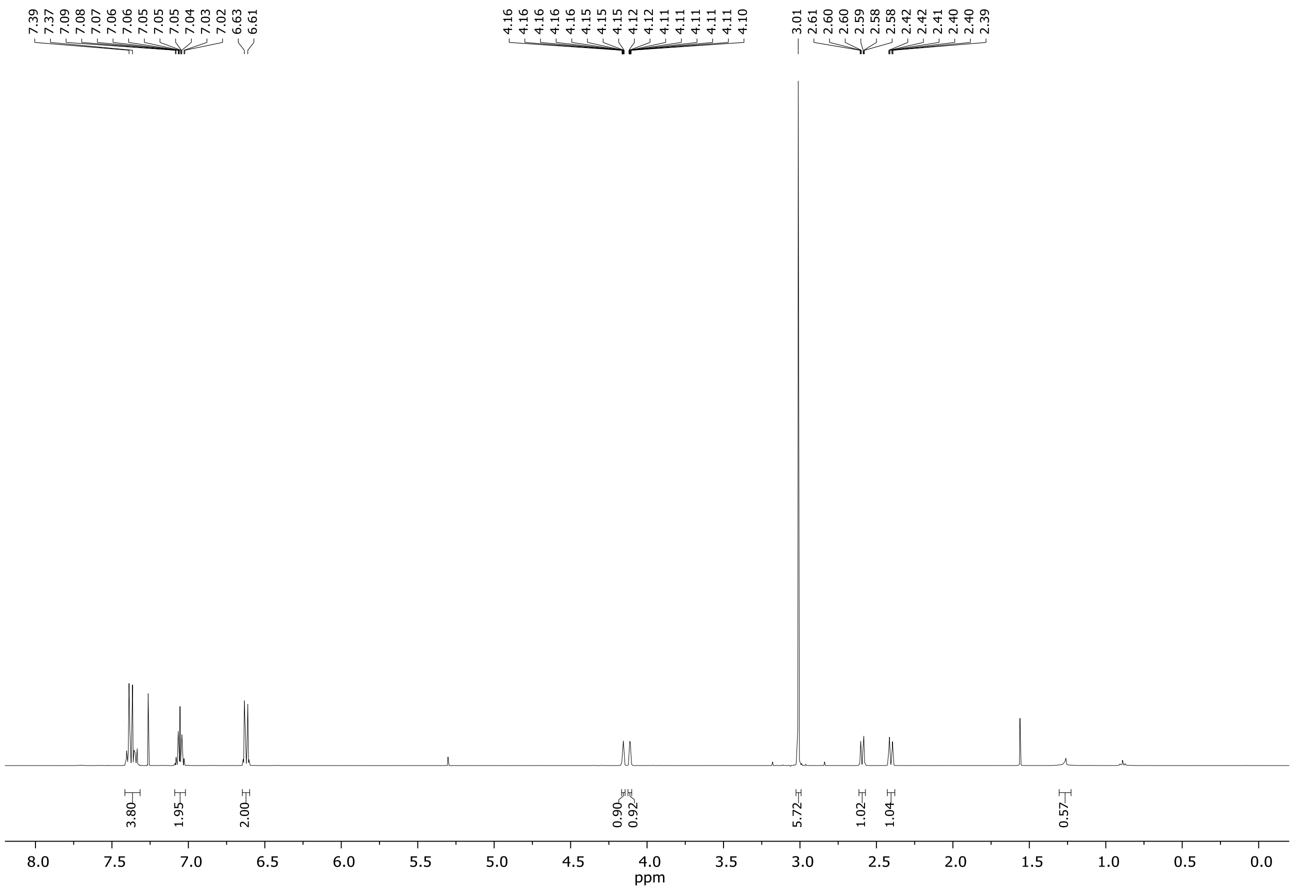

Supplementary Figure 8: ^{1}H NMR (500 MHz) of **7** in $CDCl_3$.

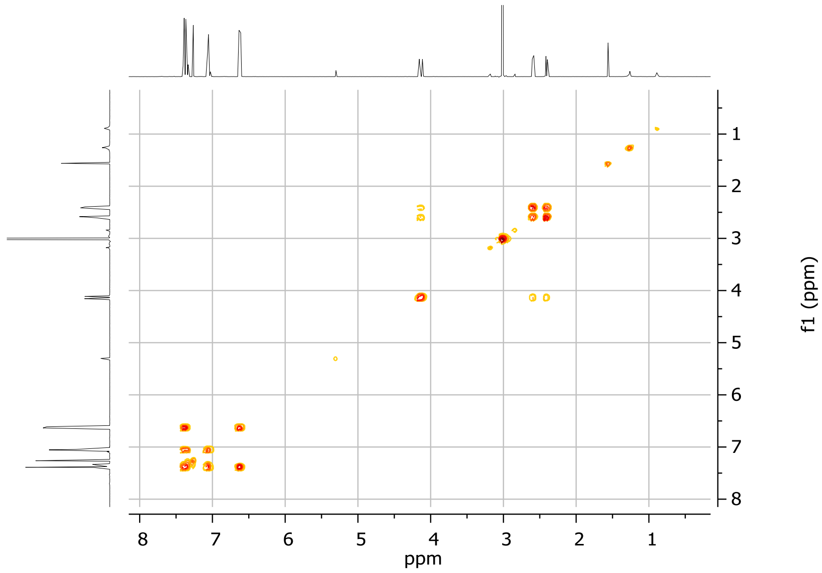

Supplementary Figure 9: COSY NMR (400 MHz) of **7** in $CDCl_3$.

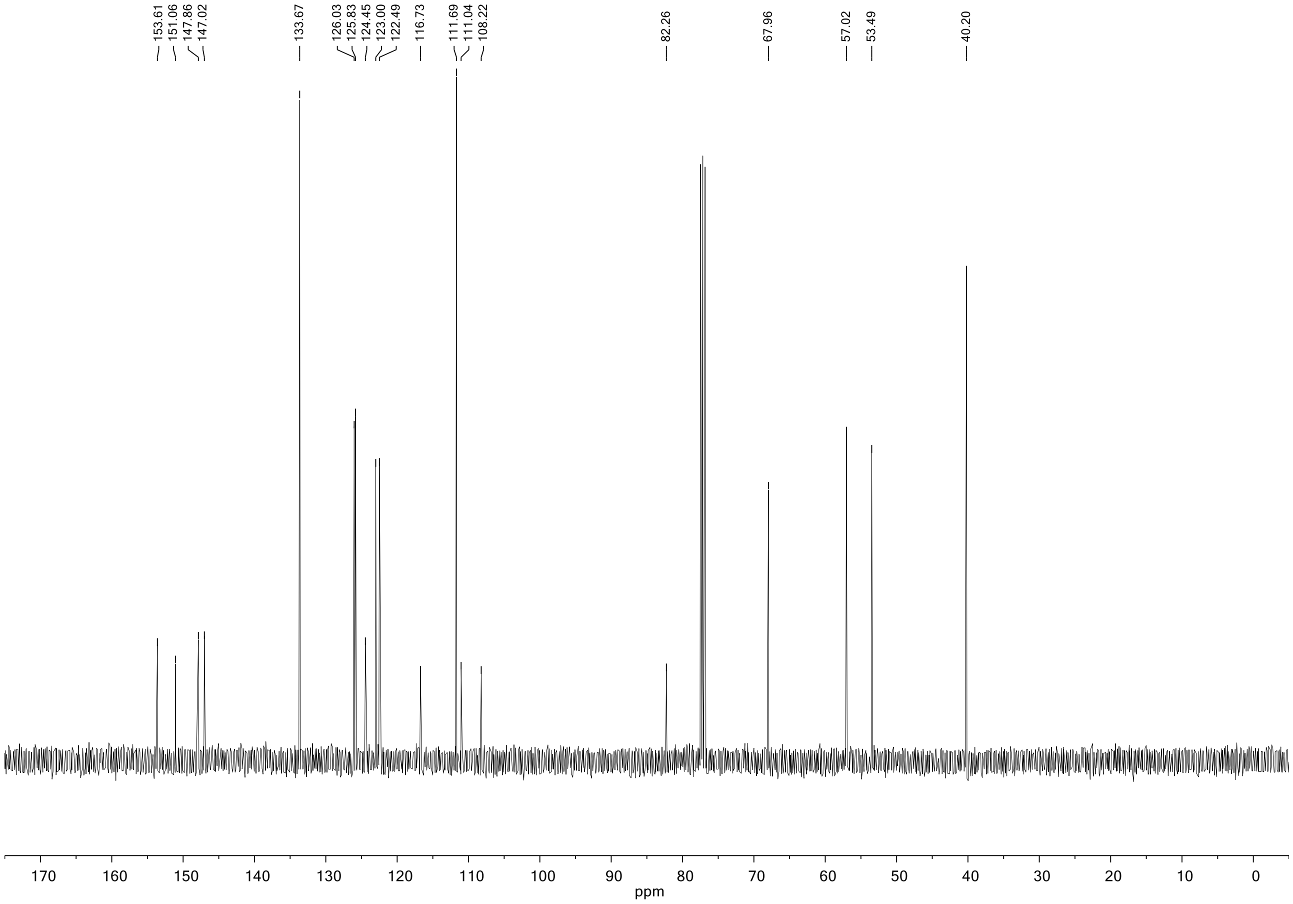

Supplementary Figure 10: ^{13}C NMR (100 MHz) of **7** in $CDCl_3$.

Compound 8

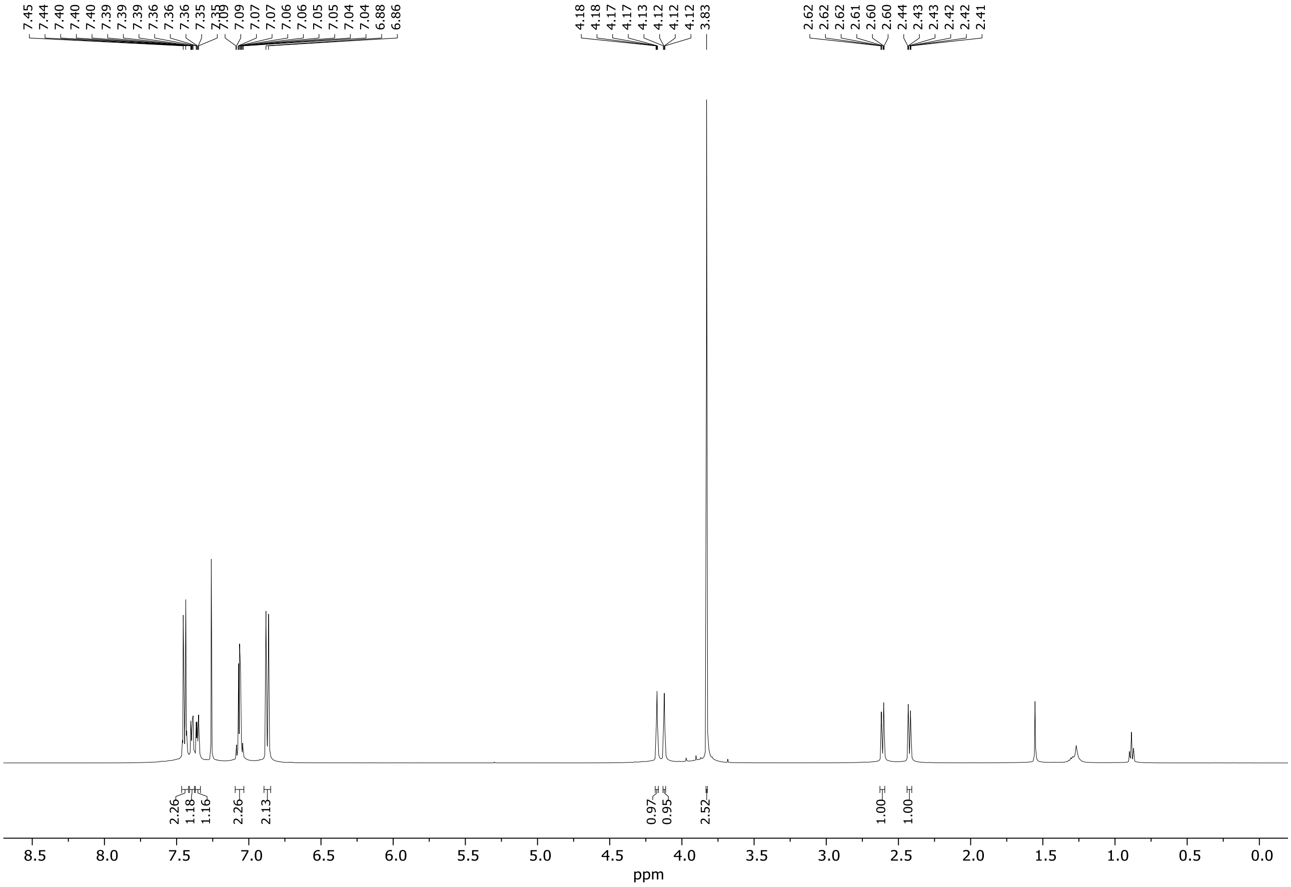

Supplementary Figure 11: ^{1}H NMR (500 MHz) of **8** in $CDCl_3$.

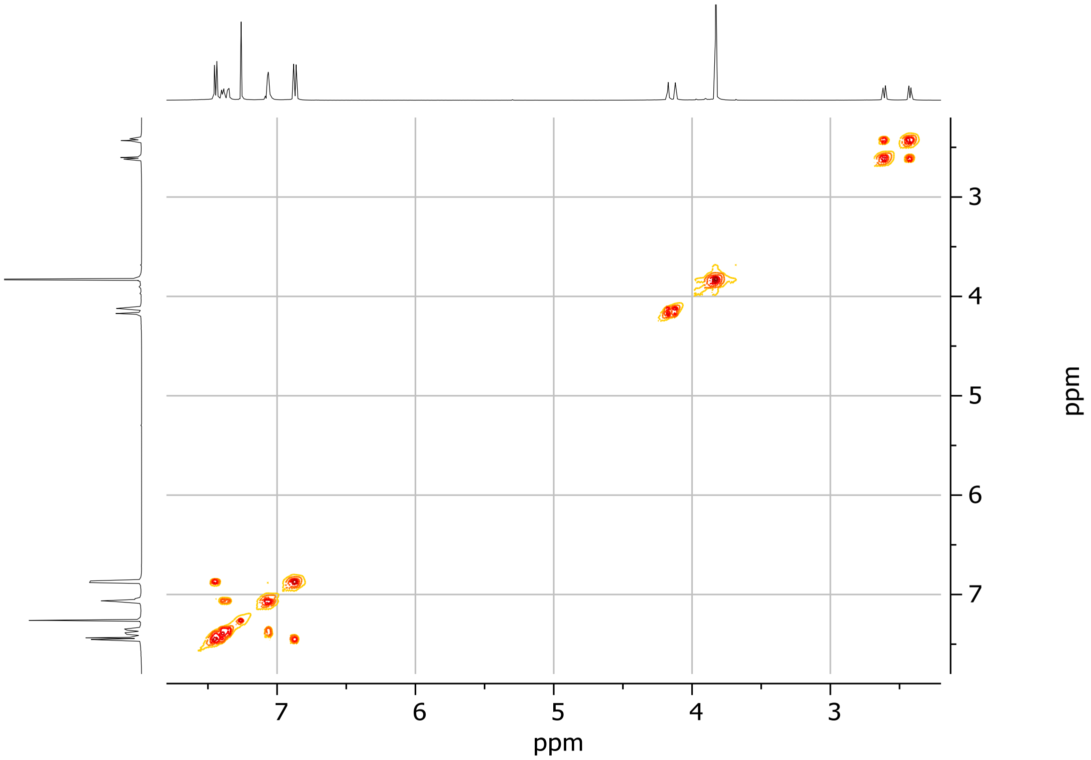

Supplementary Figure 12: COSY NMR (500 MHz) of **8** in $CDCl_3$.

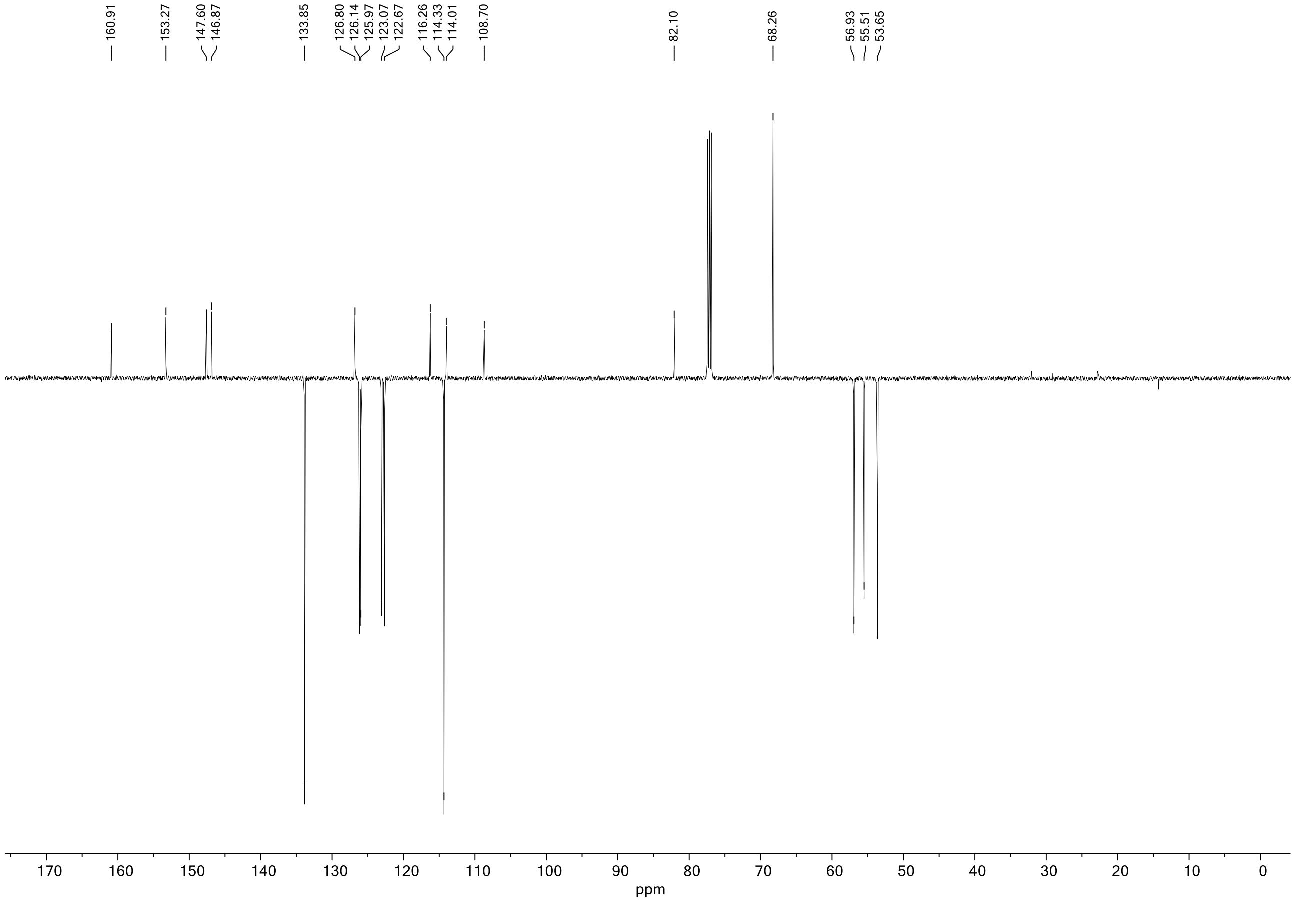

Supplementary Figure 13: ^{13}C NMR APT (126 MHz) of **8** in $CDCl_3$.

Compound 9

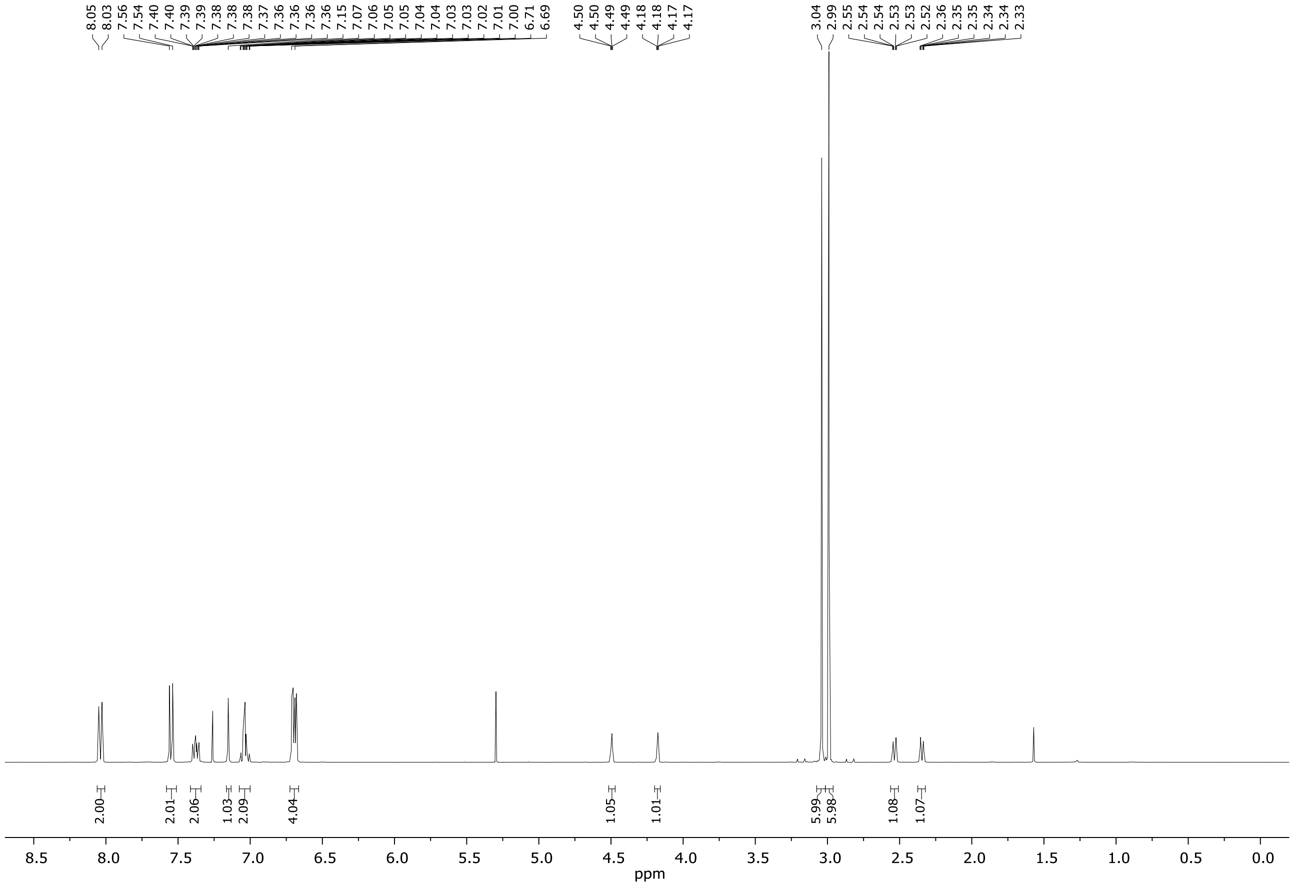

Supplementary Figure 14: ^{1}H NMR (400 MHz) of **9** in $CDCl_3$.

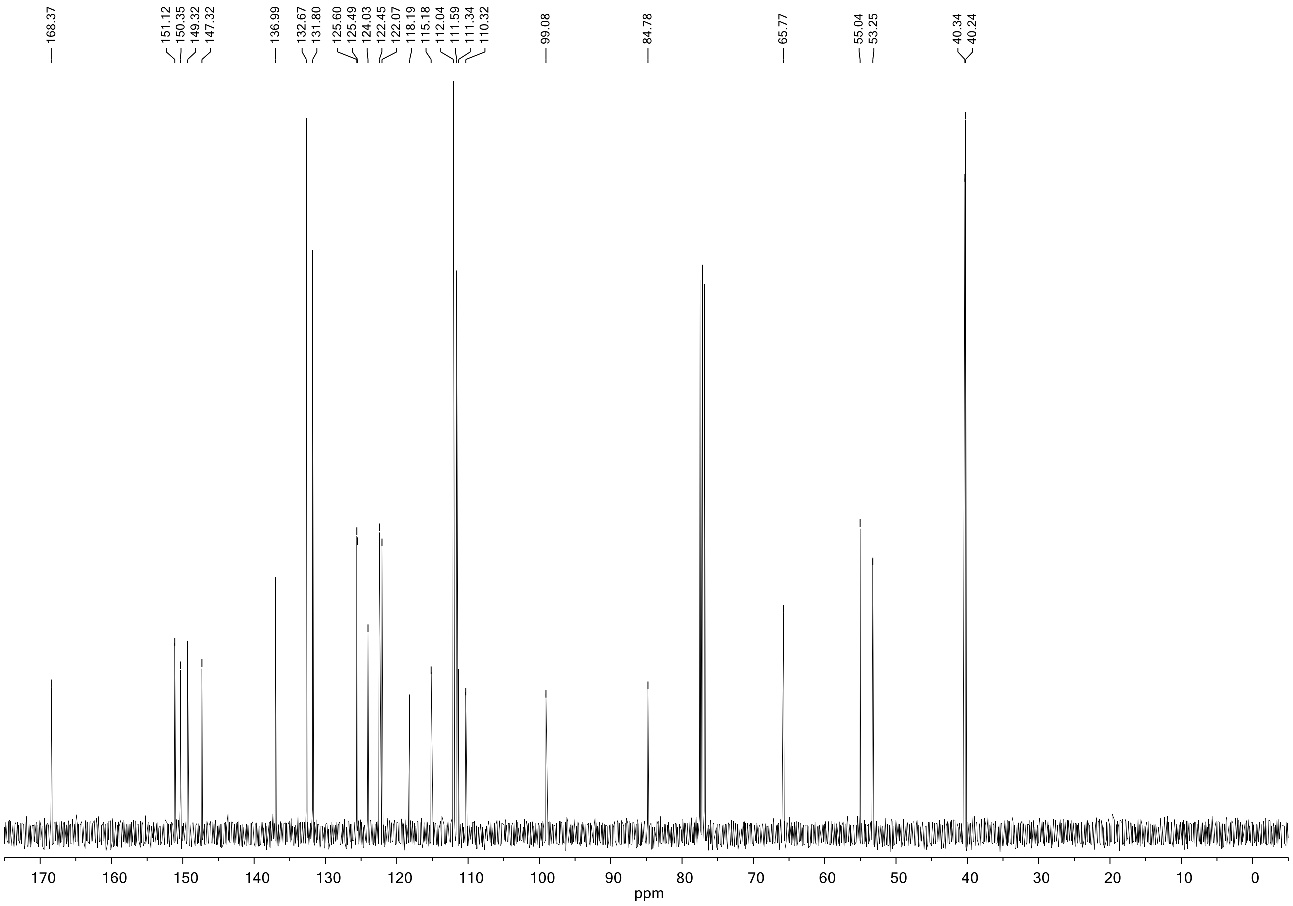

Supplementary Figure 15: ^{13}C NMR (100 MHz) of **9** in $CDCl_3$.

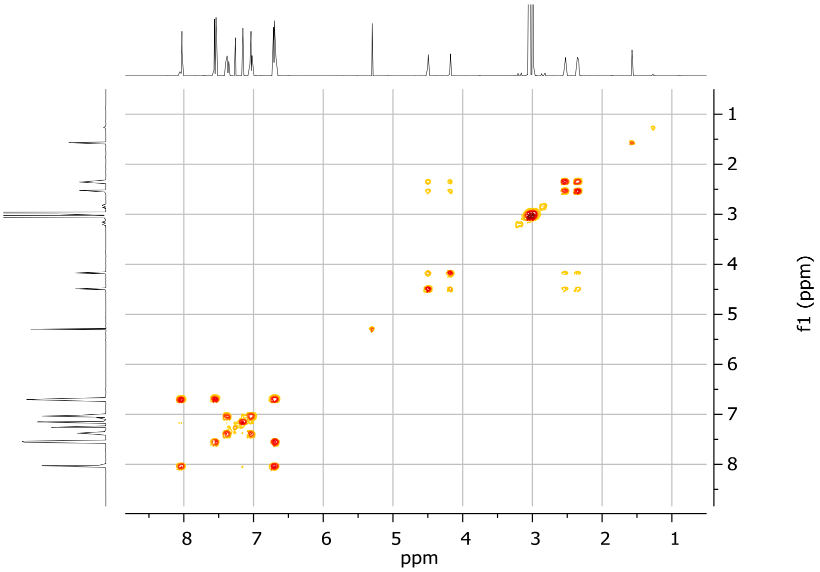

Supplementary Figure 16: COSY NMR (400 MHz) of **9** in $CDCl_3$.

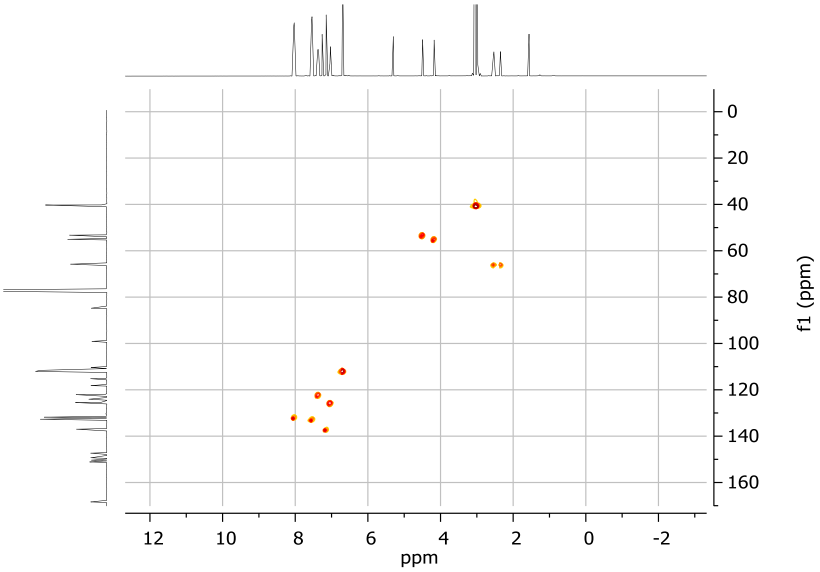

Supplementary Figure 17: HSQC NMR of **9** in $CDCl_3$.

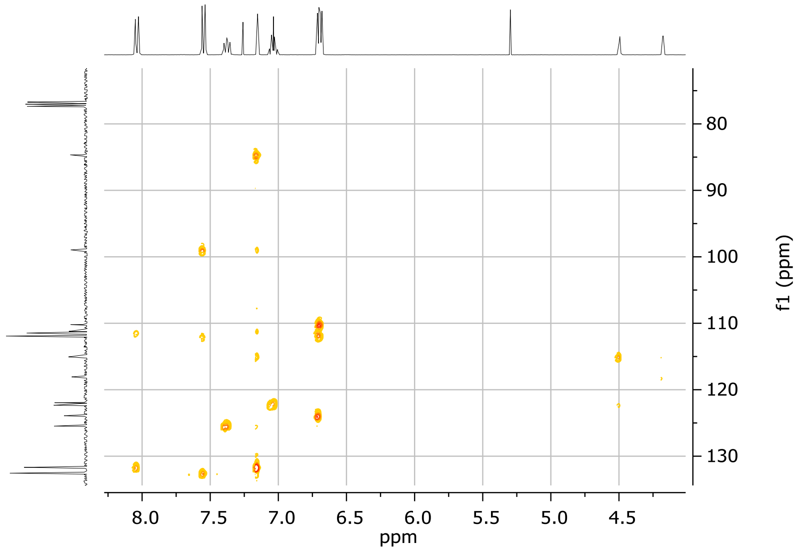

Supplementary Figure 18: HMBC NMR of **9** in $CDCl_3$.

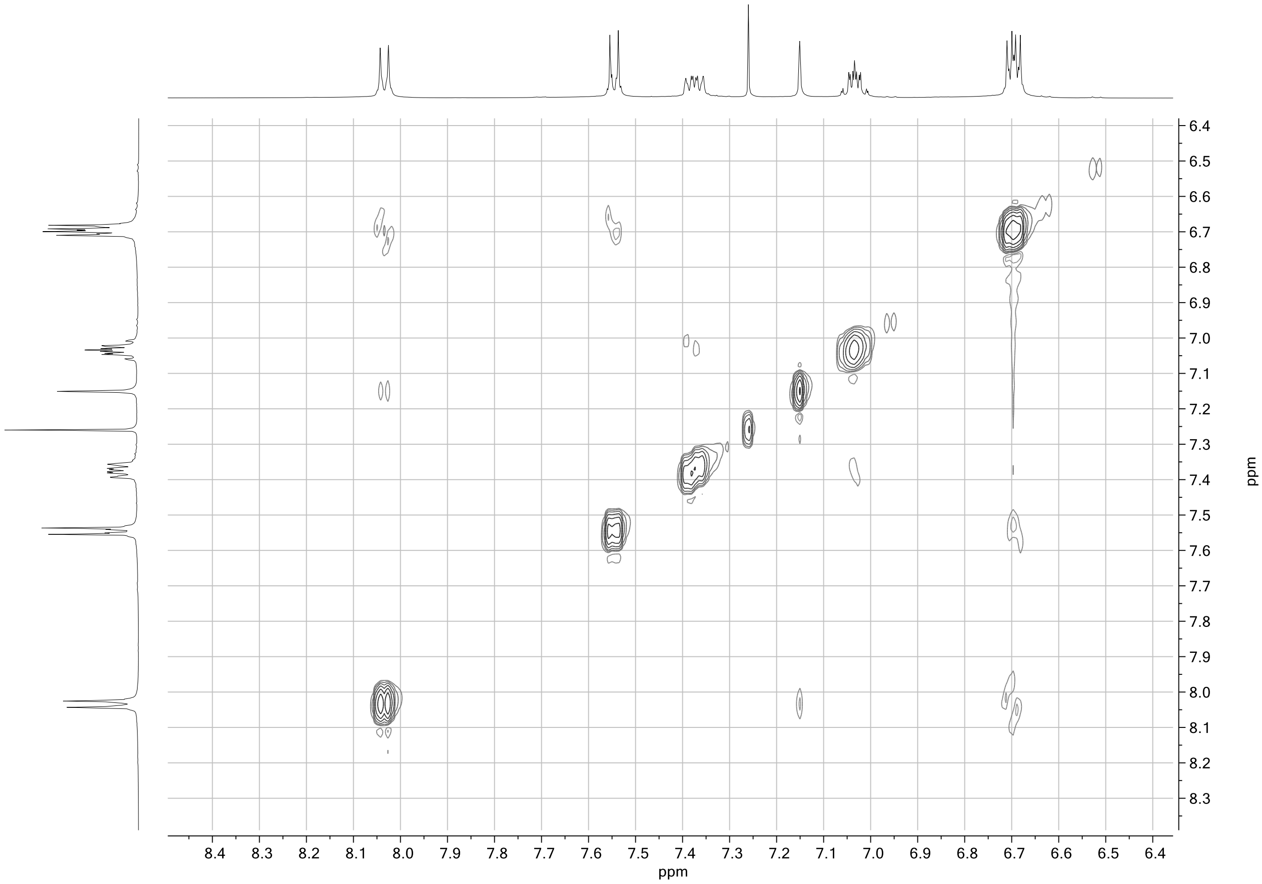

Supplementary Figure 19: NOESY NMR (500 MHz) of **9** in $CDCl_3$, zoomed in the aromatic region.

Compound 10

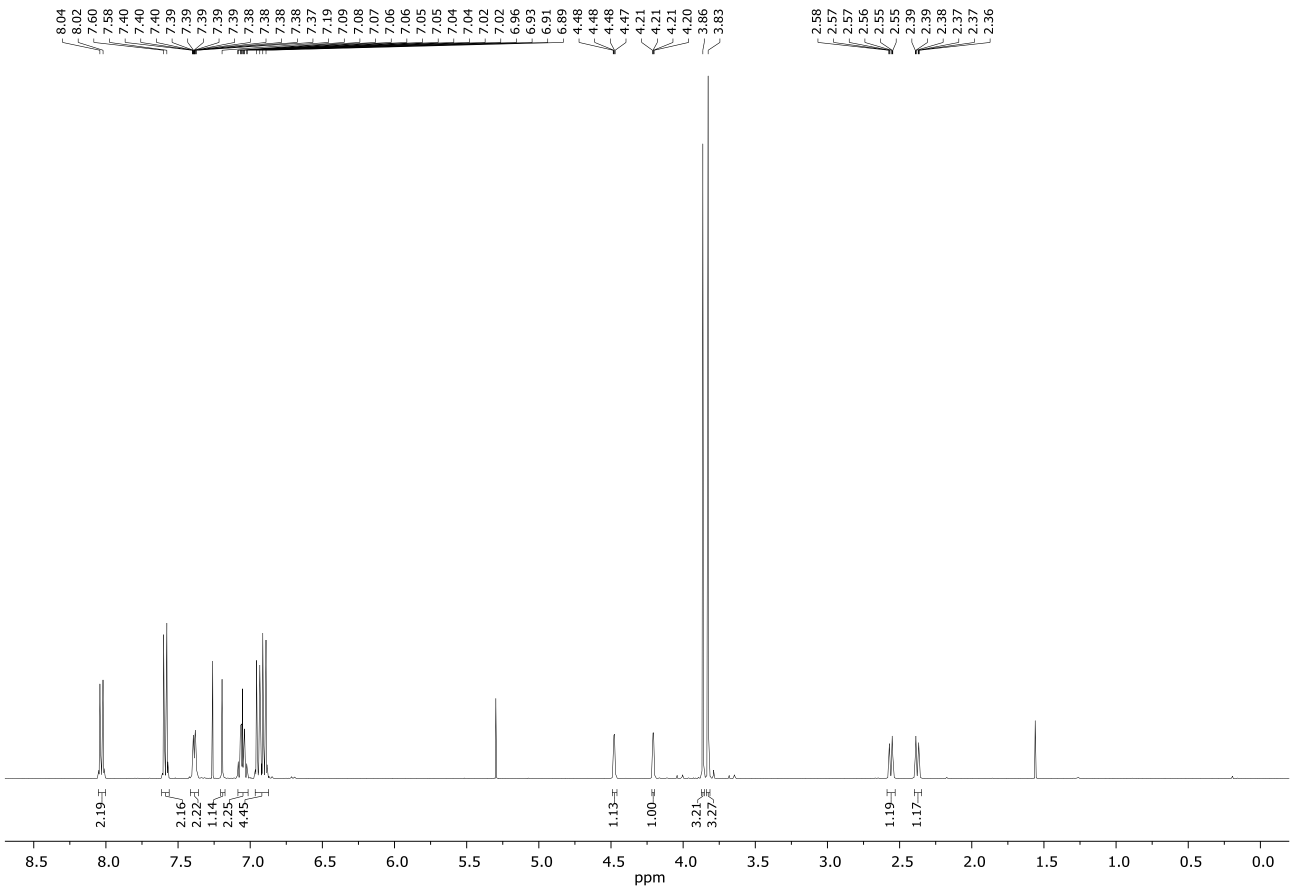

Supplementary Figure 20: ^{1}H NMR (400 MHz) of **10** in $CDCl_3$.

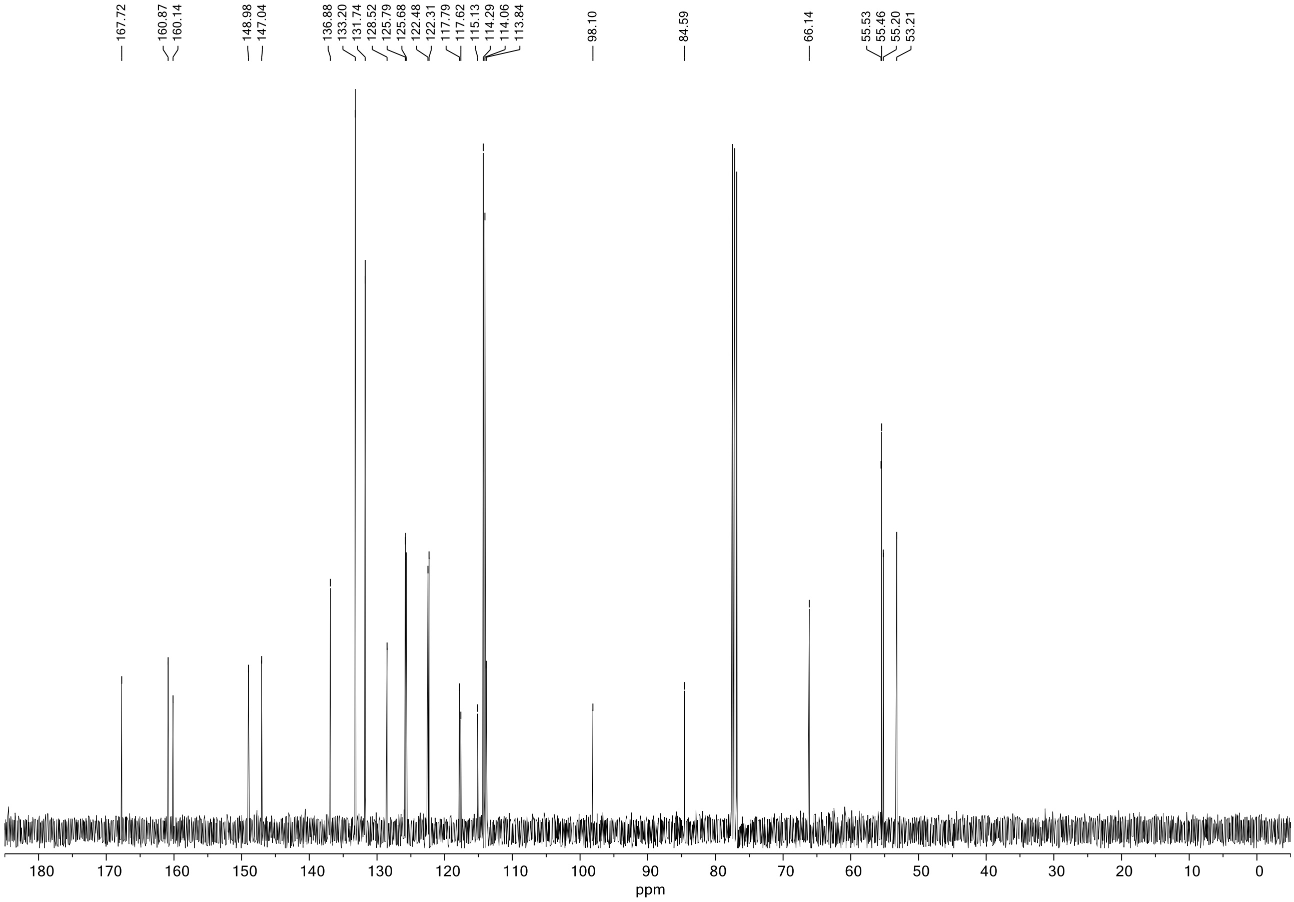

Supplementary Figure 21: ^{13}C NMR (100 MHz) of **10** in $CDCl_3$.

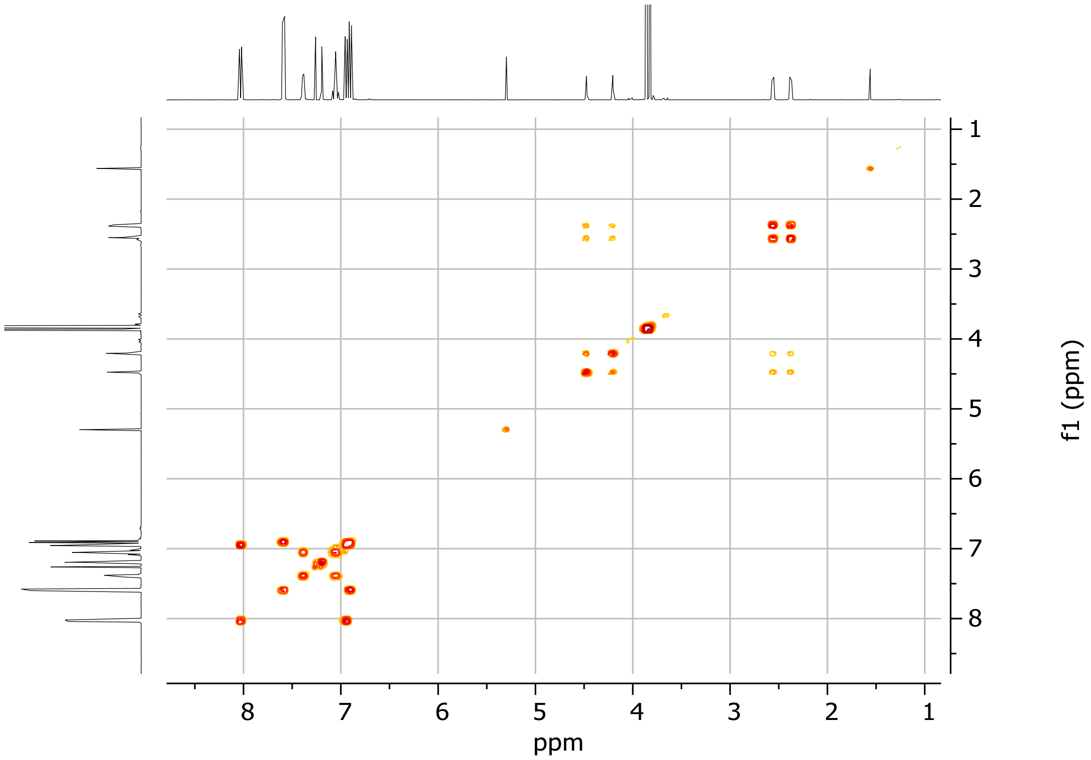

Supplementary Figure 22: COSY NMR (400 MHz) of **10** in $CDCl_3$.

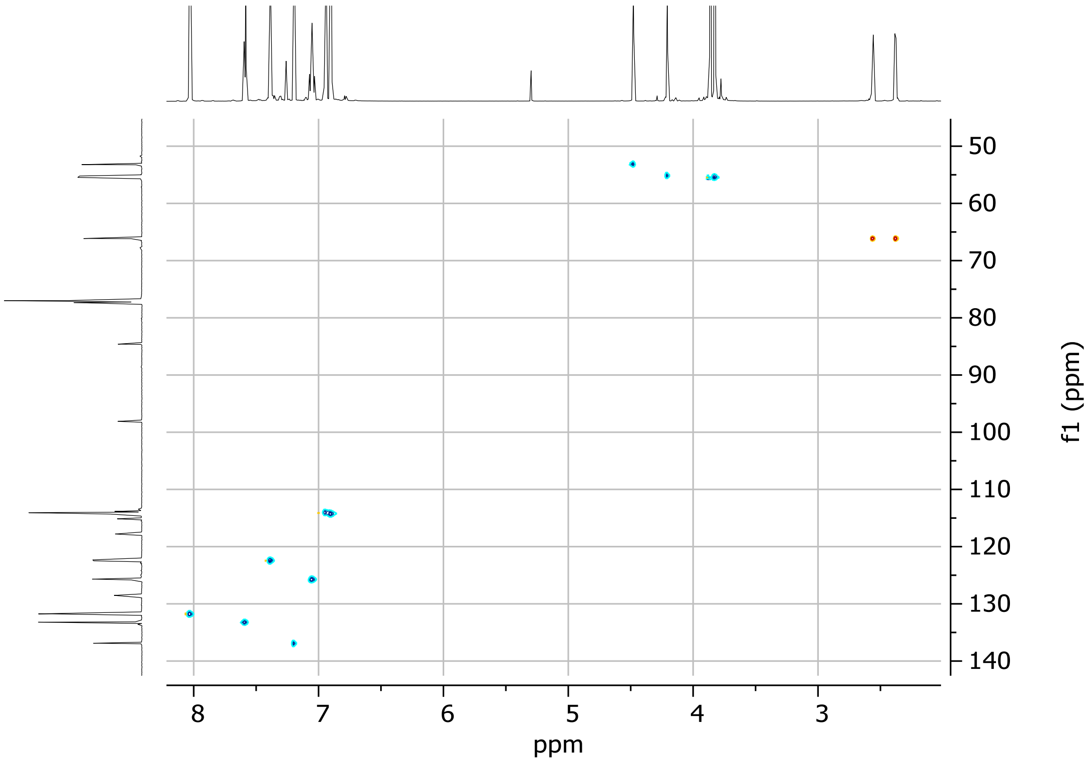

Supplementary Figure 23: HSQC NMR of **10** in $CDCl_3$.

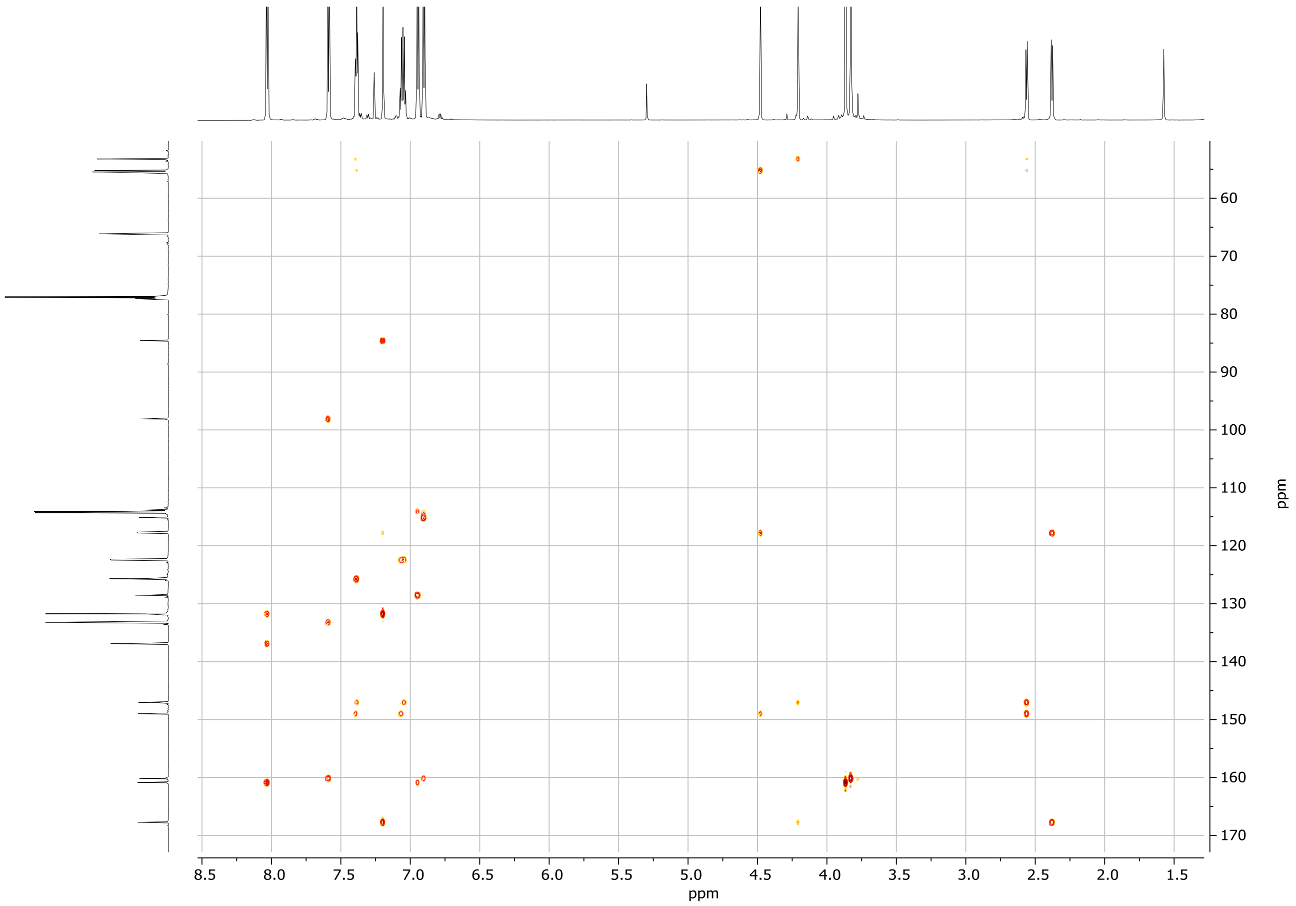

Supplementary Figure 24: HMBC NMR of **10** in $CDCl_3$.

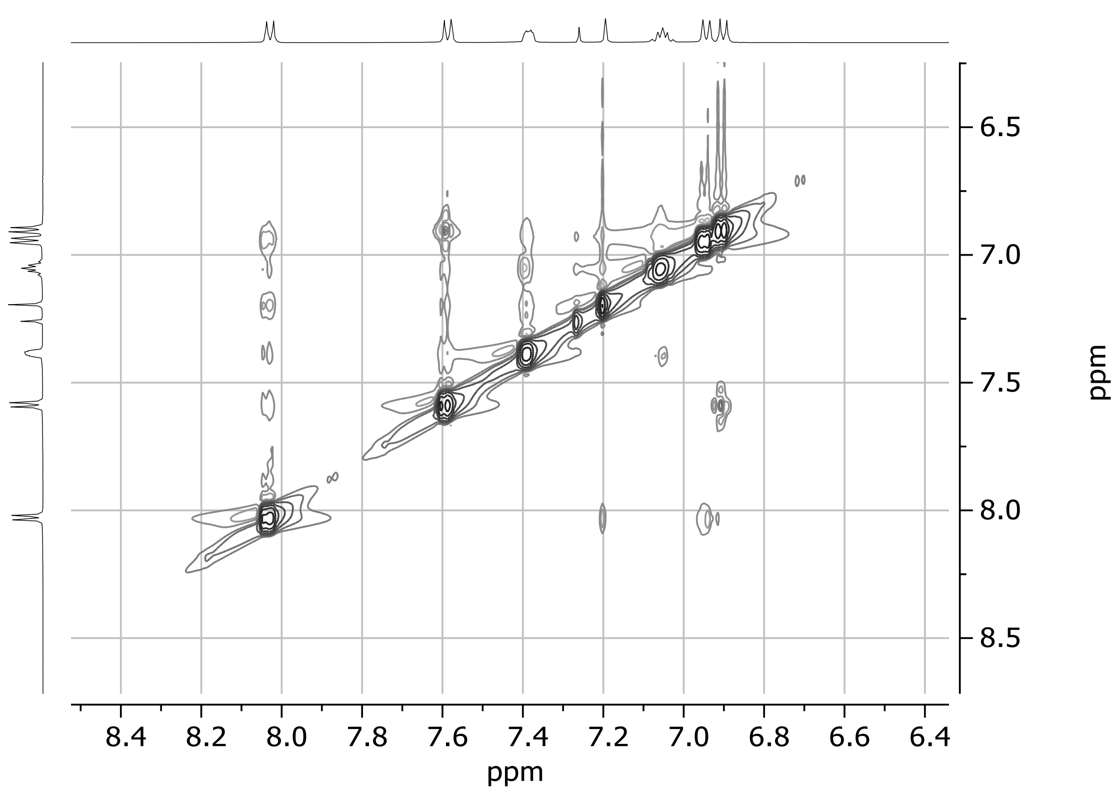

Supplementary Figure 25: NOESY NMR of **10** in $CDCl_3$.

Compound 13

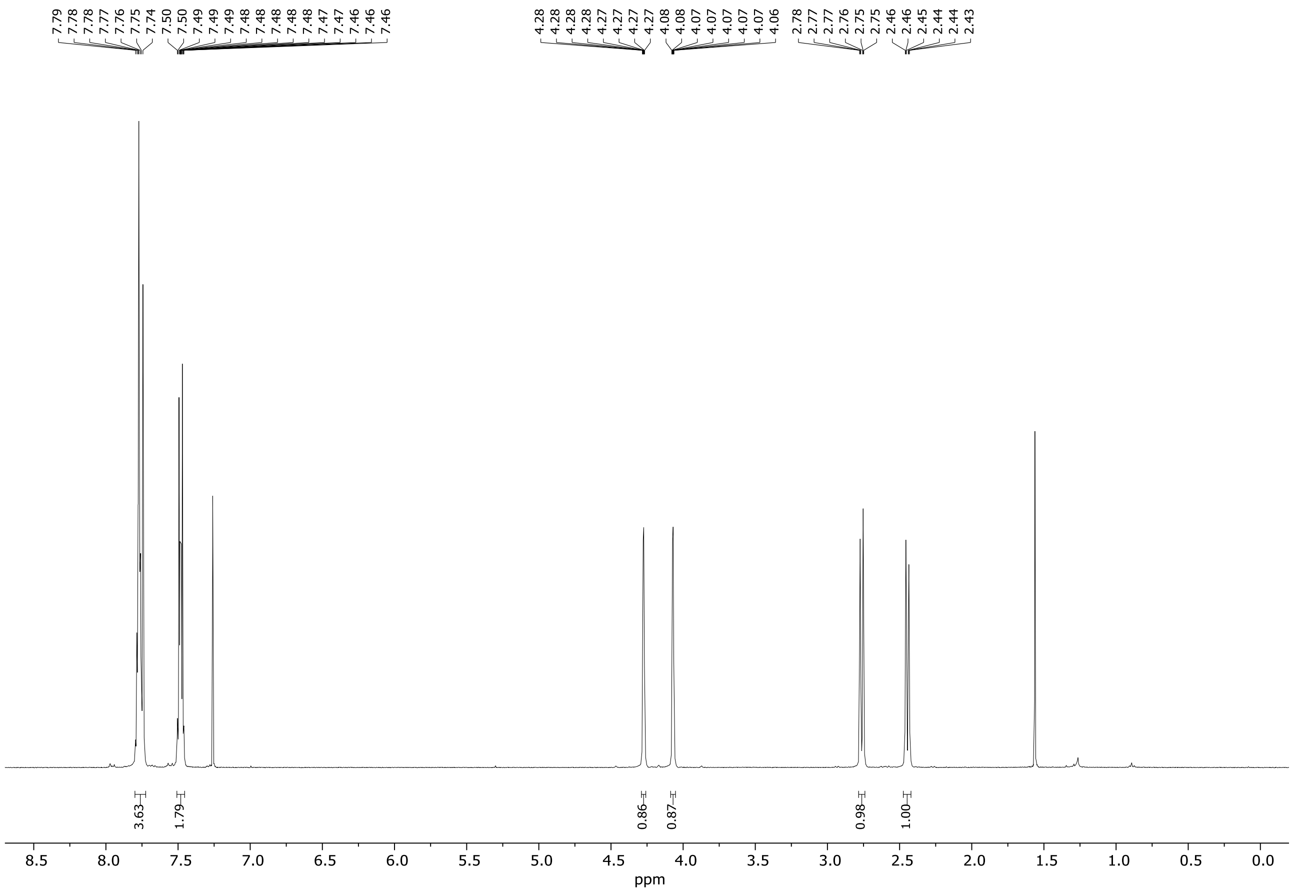

Supplementary Figure 26: ^{1}H NMR (400 MHz) of **13** in $CDCl_3$.

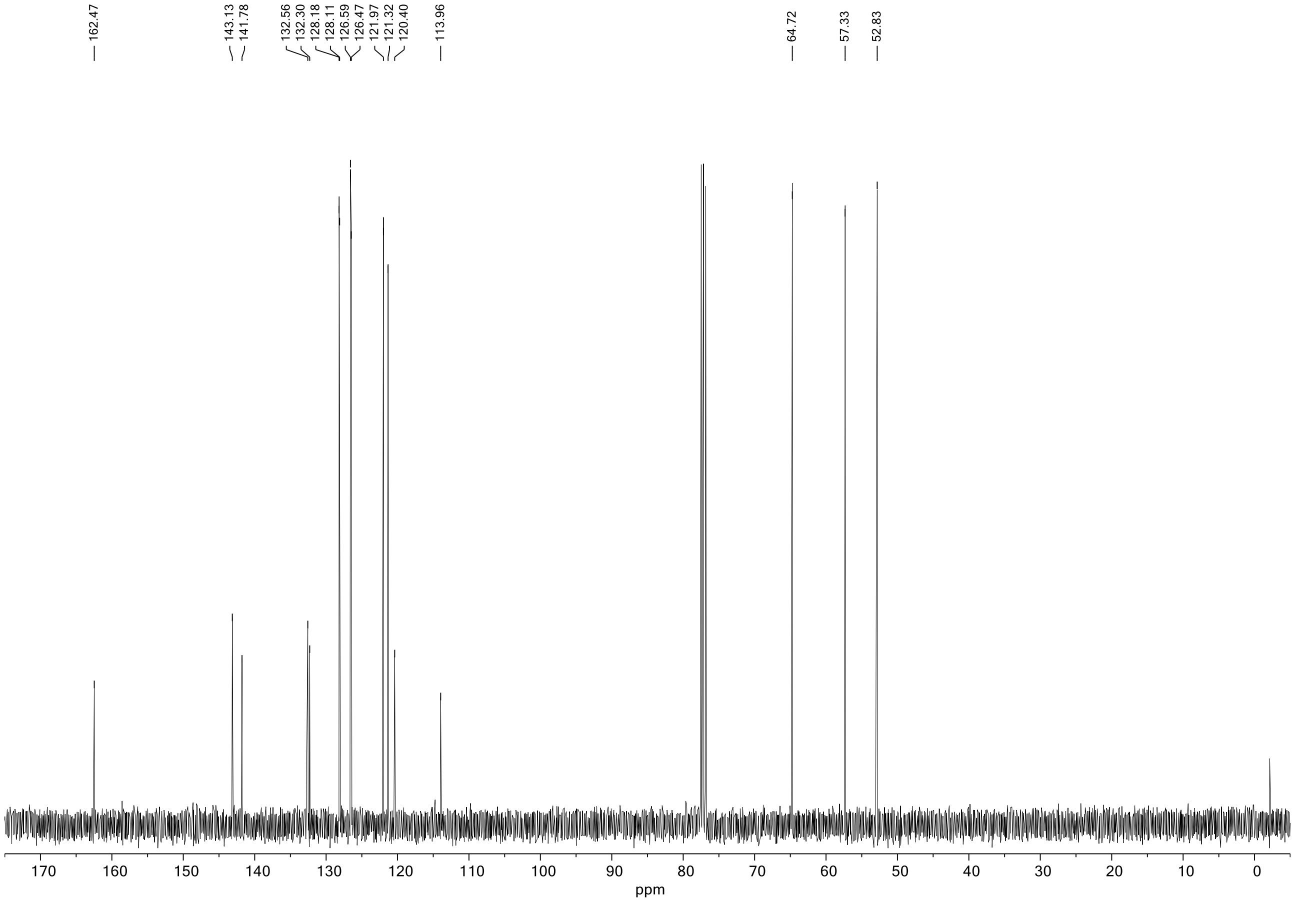

Supplementary Figure 27: ^{13}C NMR (100 MHz) of **13** in $CDCl_3$.

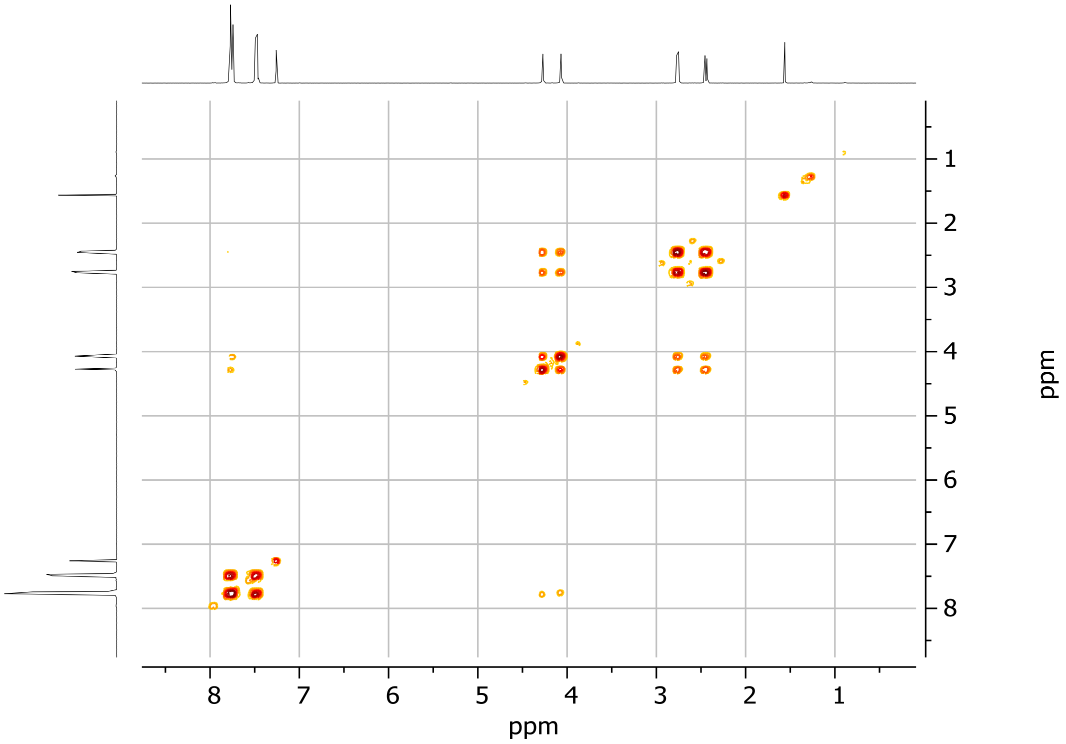

Supplementary Figure 28: COSY NMR (400 MHz) of **13** in $CDCl_3$.

Compound 14

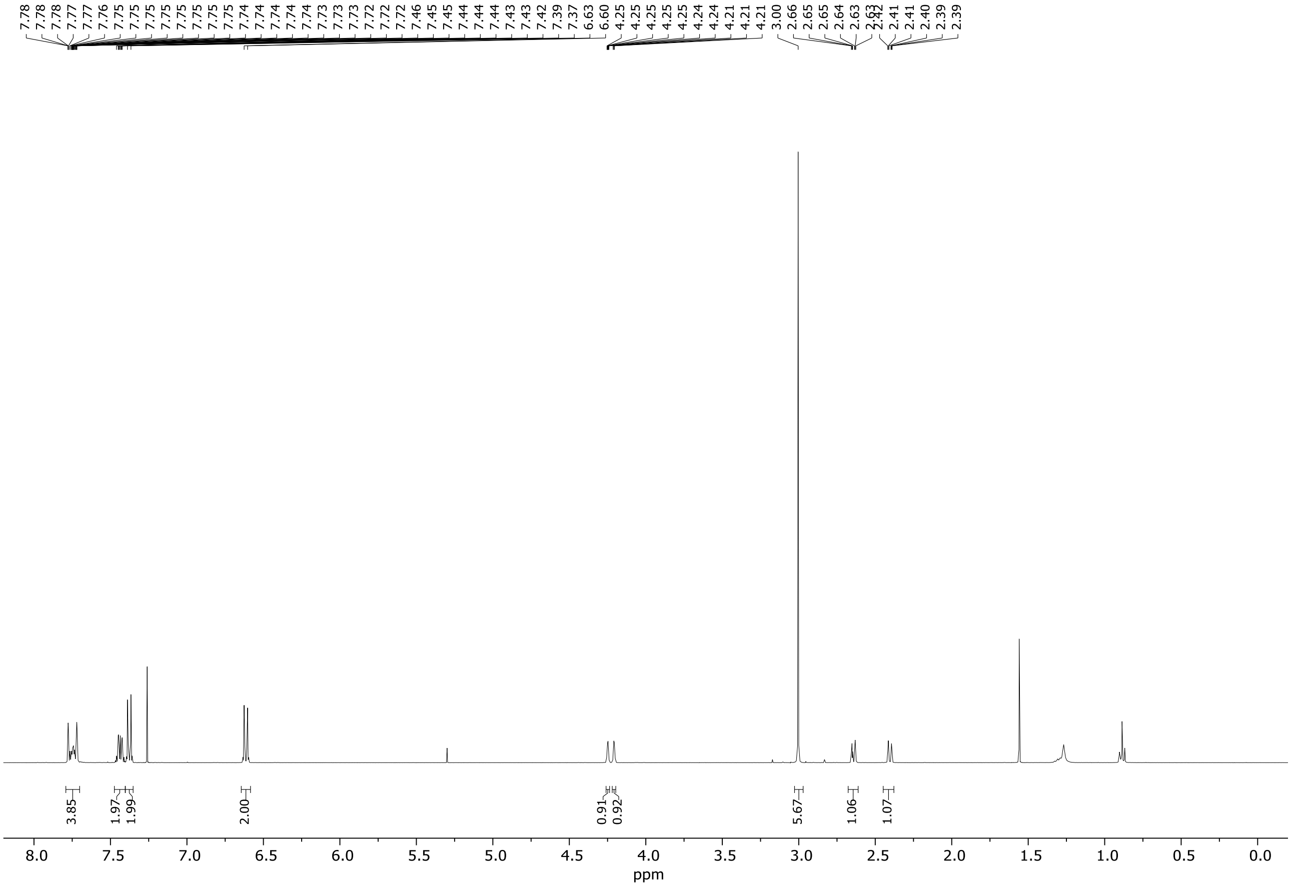

Supplementary Figure 29: ^{1}H NMR (400 MHz) of **14** in $CDCl_3$.

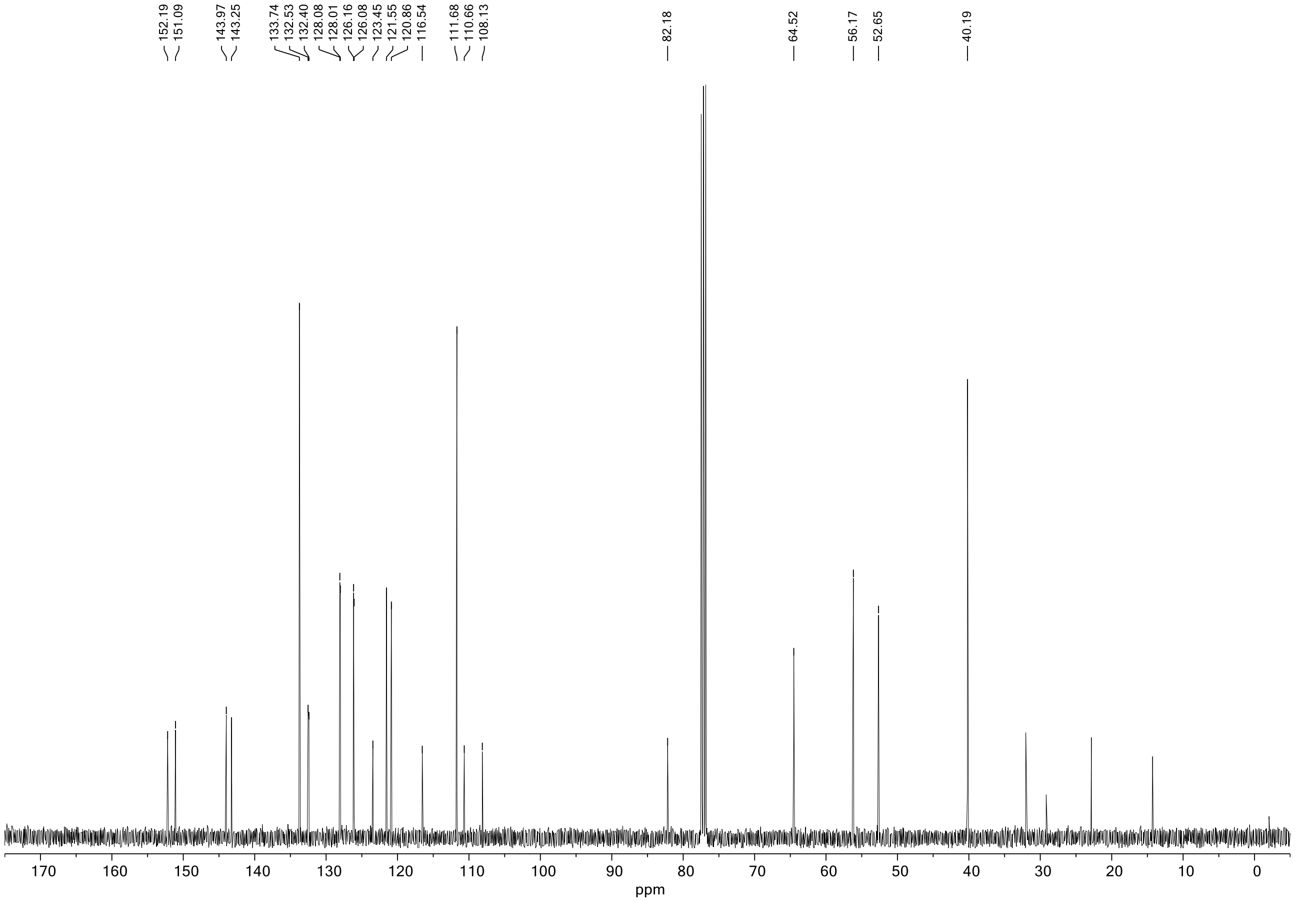

Supplementary Figure 30: ^{13}C NMR (400 MHz) of **14** in $CDCl_3$.

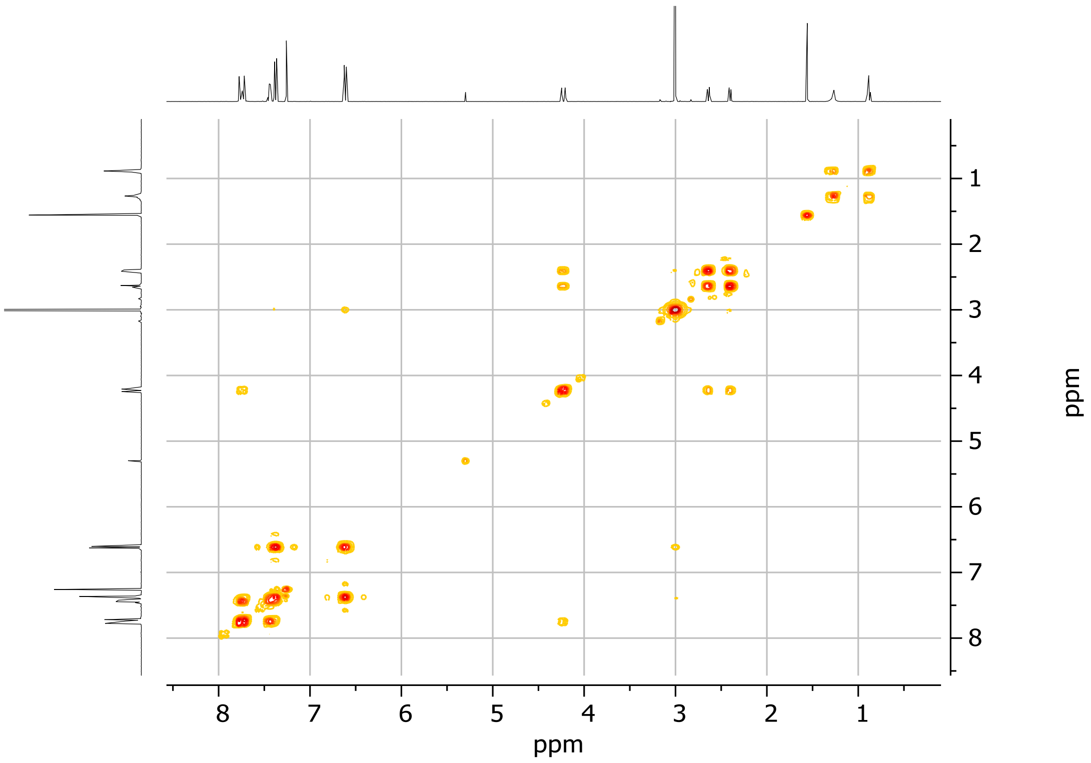

Supplementary Figure 31: COSY NMR (400 MHz) of **14** in $CDCl_3$.

Compound 15

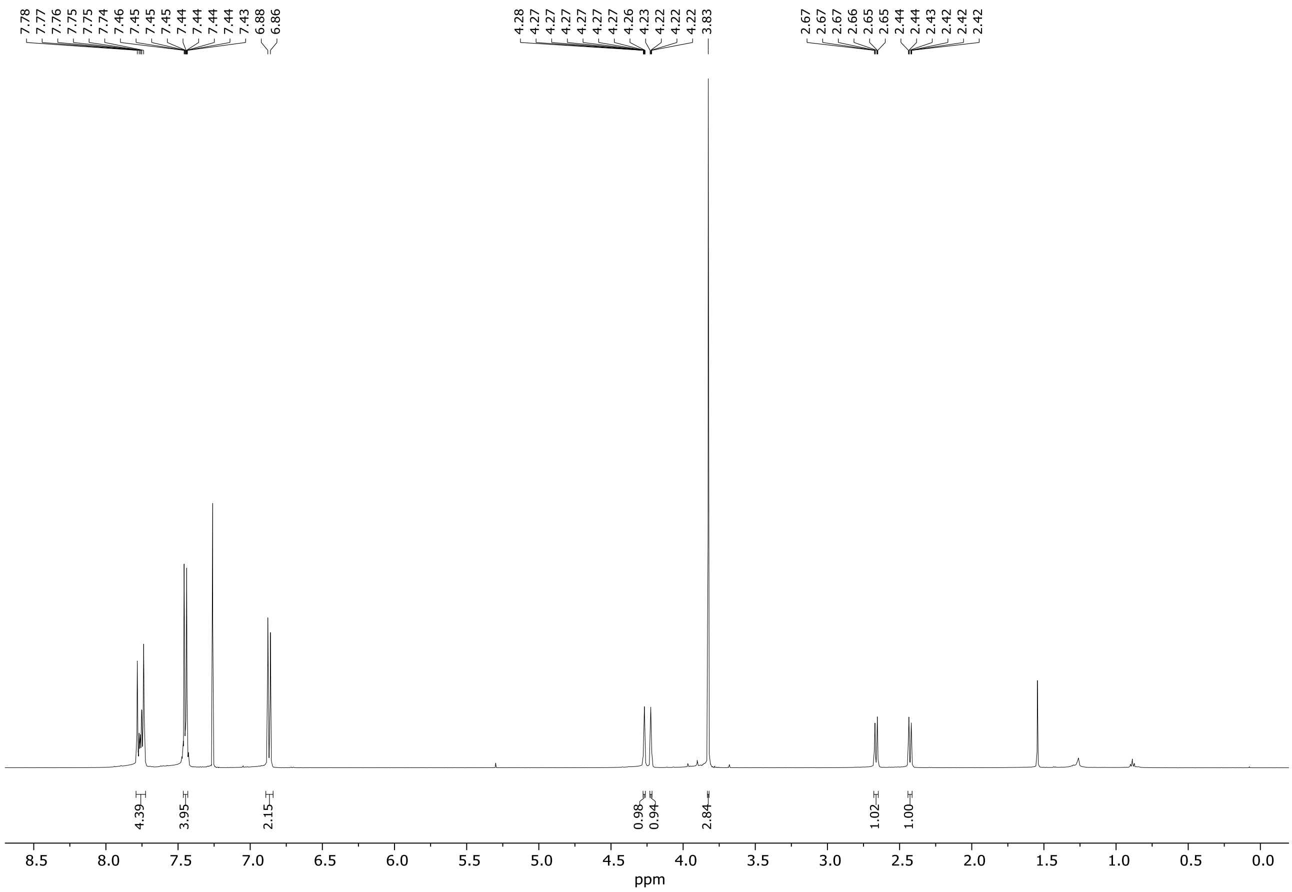

Supplementary Figure 32: ^{1}H NMR (500 MHz) of **15** in $CDCl_3$.

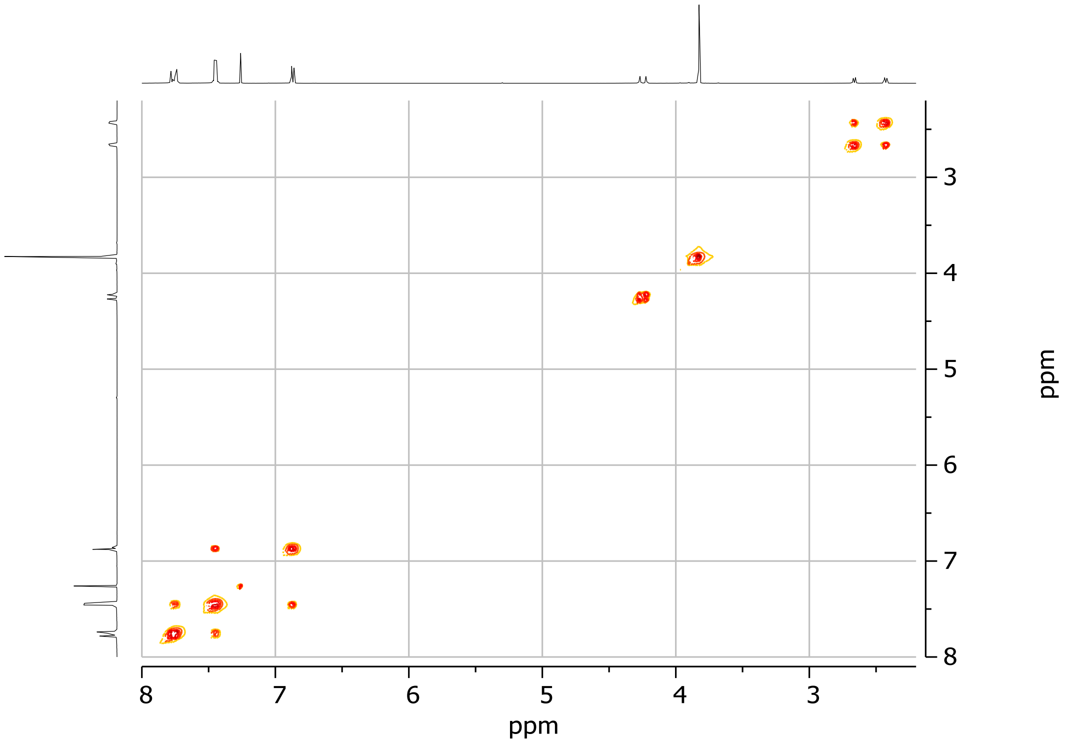

Supplementary Figure 33: COSY NMR (500 MHz) of **15** in $CDCl_3$.

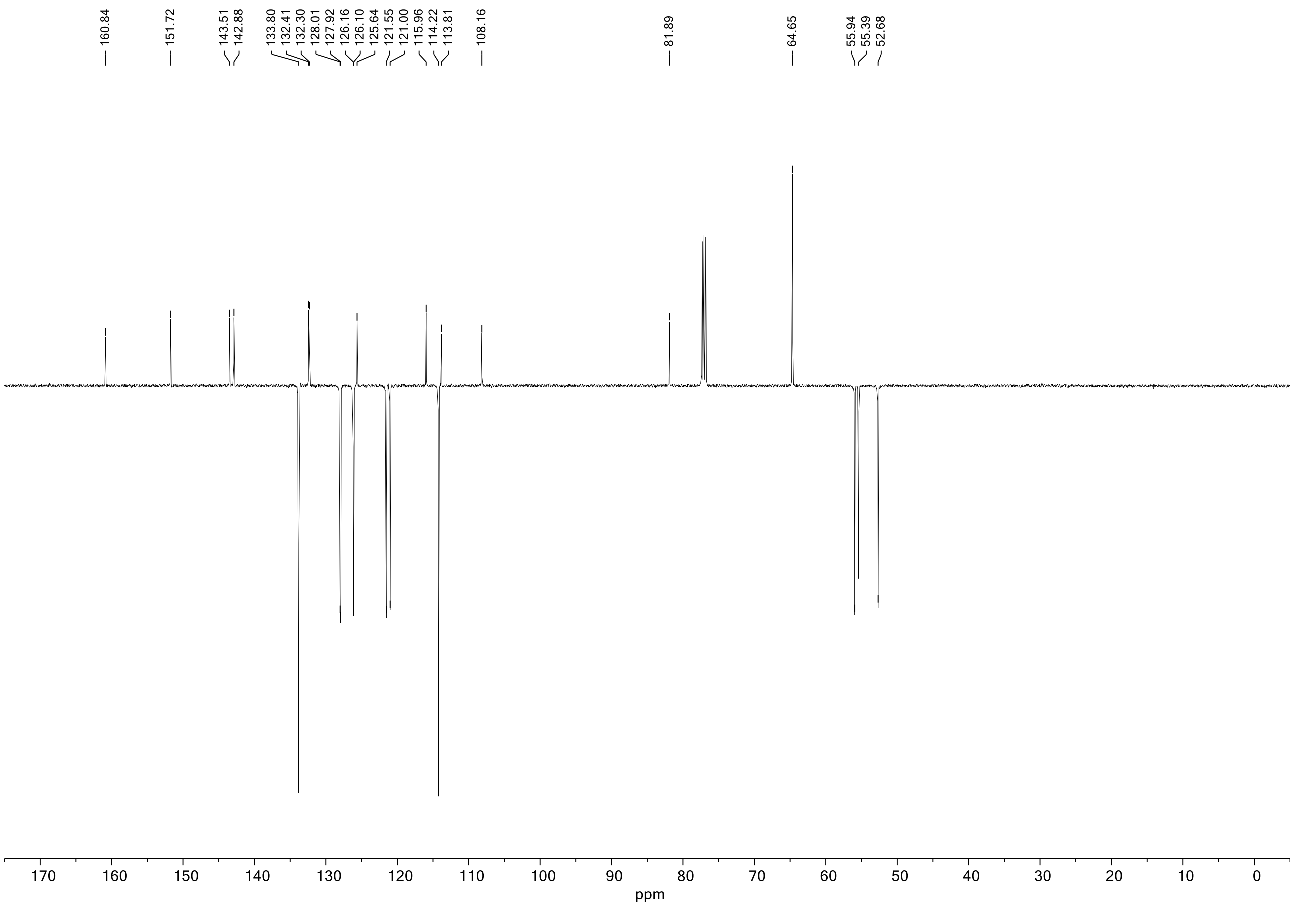

Supplementary Figure 34: ^{13}C NMR (126 MHz) of **15** in $CDCl_3$.

Compound 17

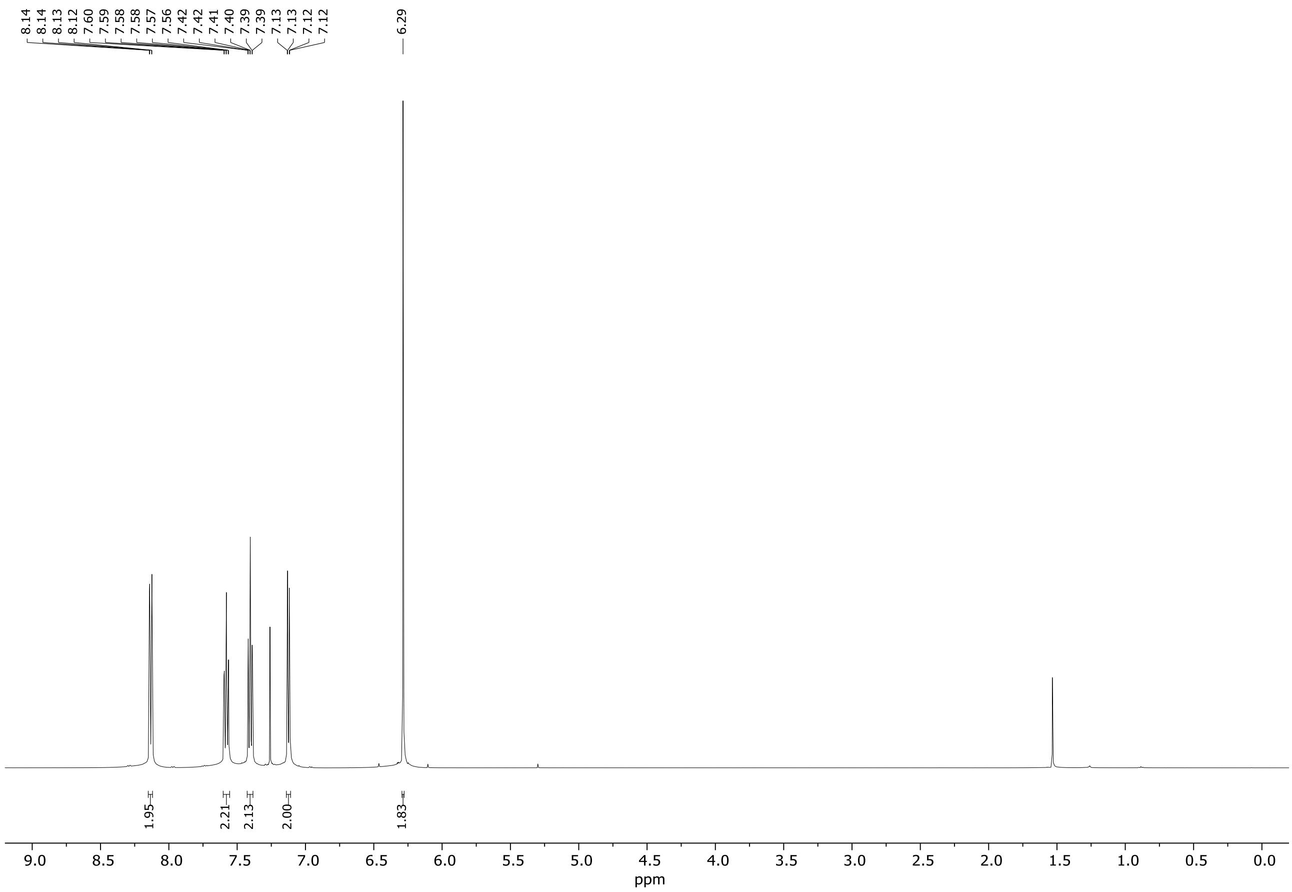

Supplementary Figure 35: ^{1}H NMR (500 MHz) of **17** in $CDCl_3$.

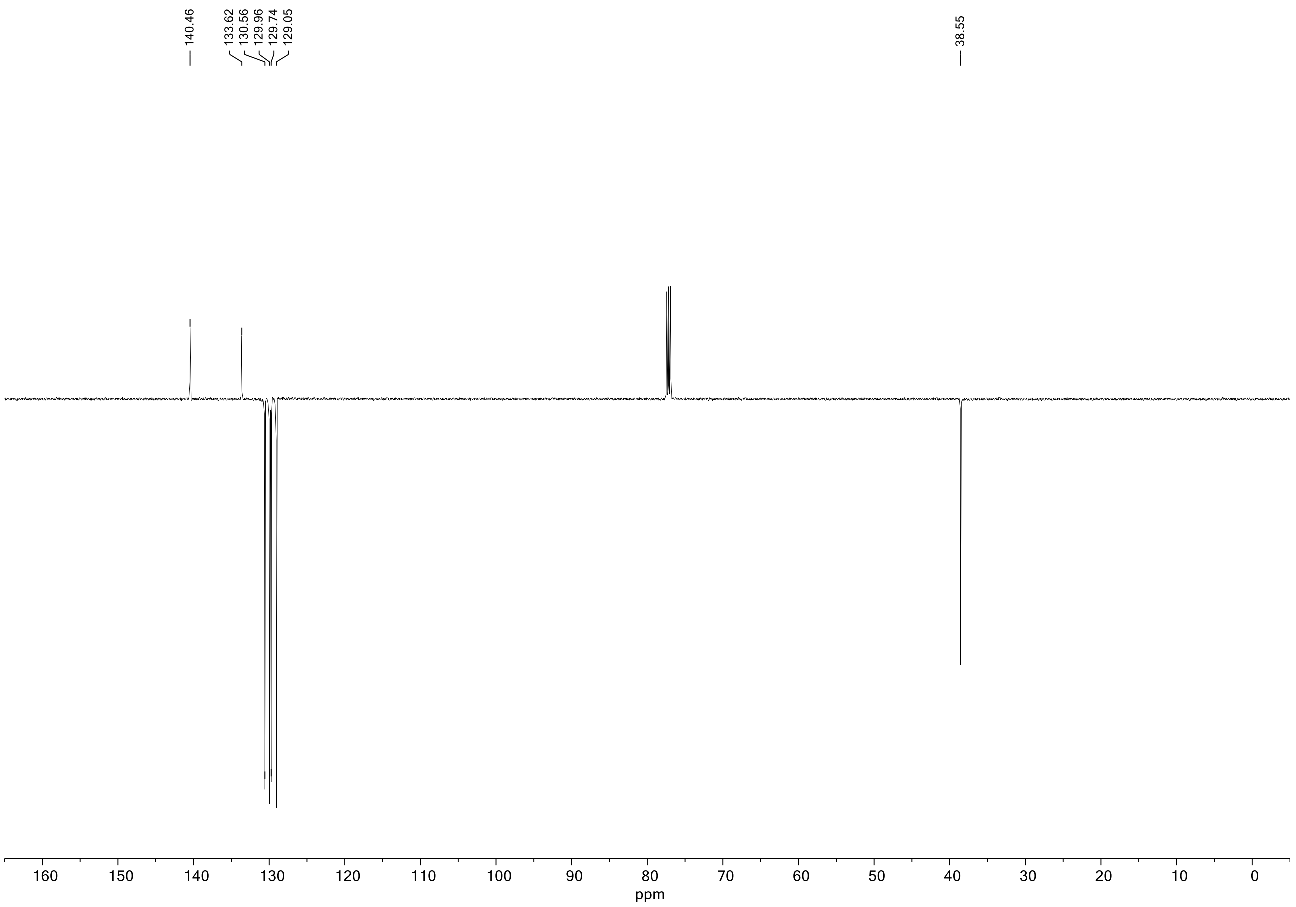

Supplementary Figure 36: ^{13}C NMR (126 MHz) of **17** in $CDCl_3$.

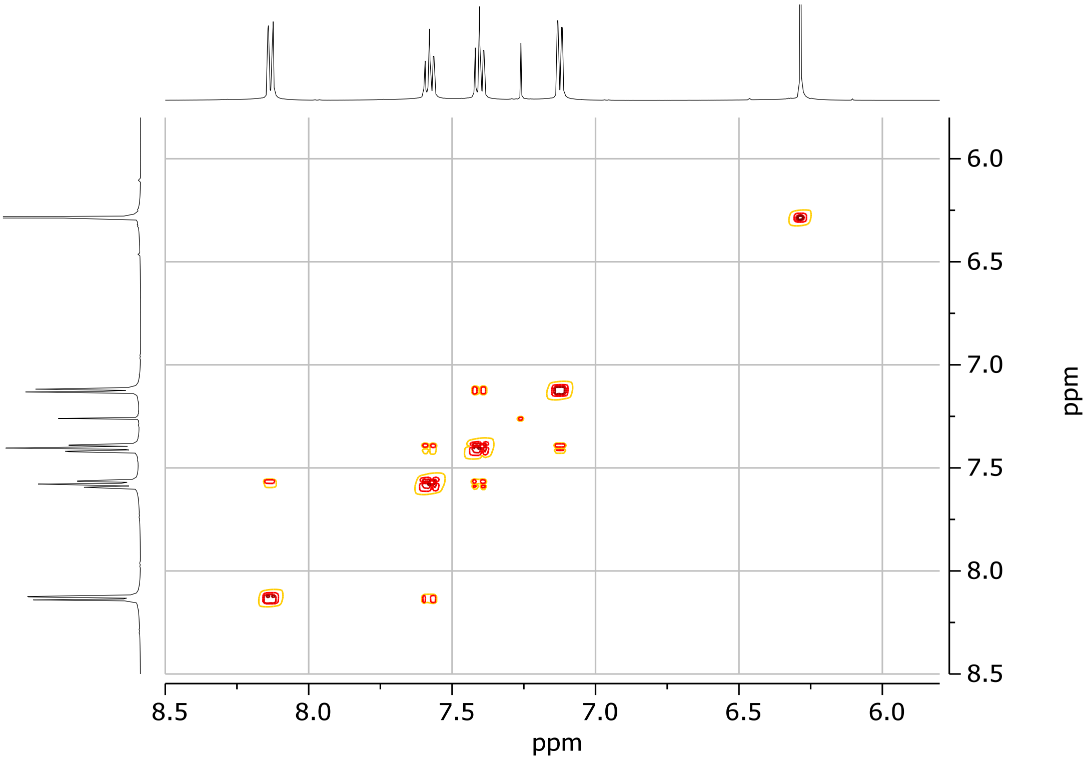

Supplementary Figure 37: COSY NMR (500 MHz) of **17** in $CDCl_3$.

Compound 18

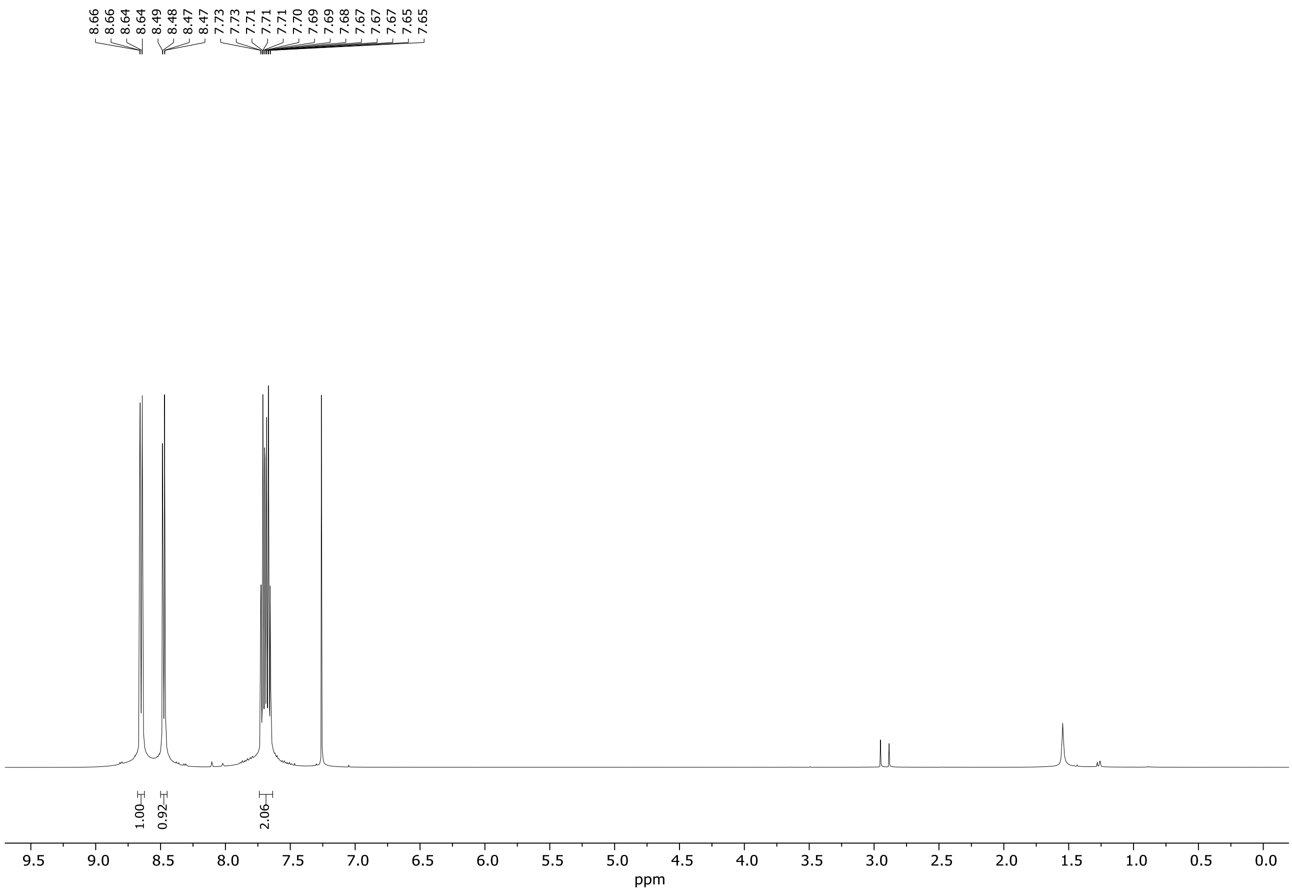

Supplementary Figure 38: ^{1}H NMR (500 MHz) of **18** in $CDCl_3$.

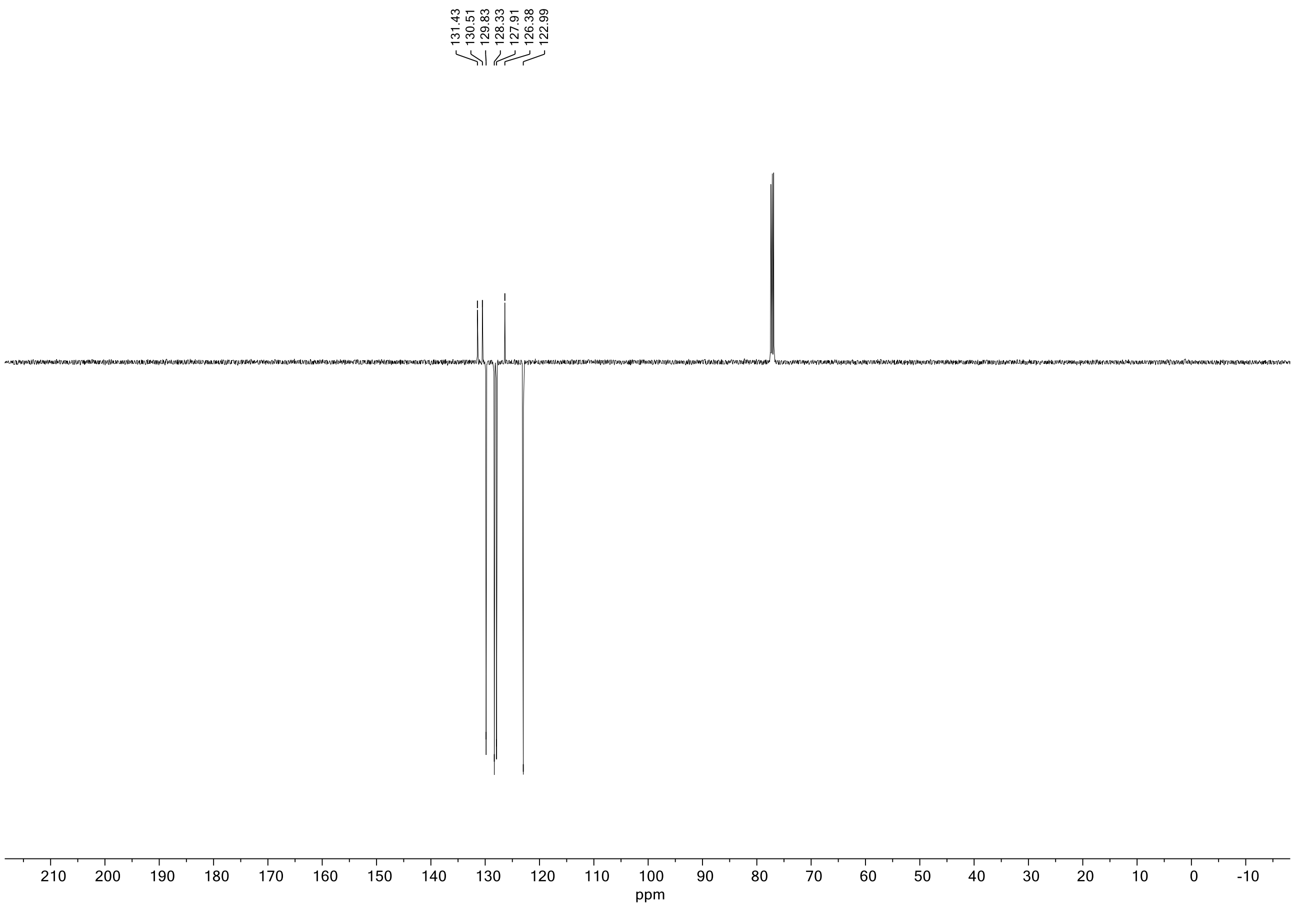

Supplementary Figure 39: ^{13}C NMR (126 MHz) of **18** in $CDCl_3$.

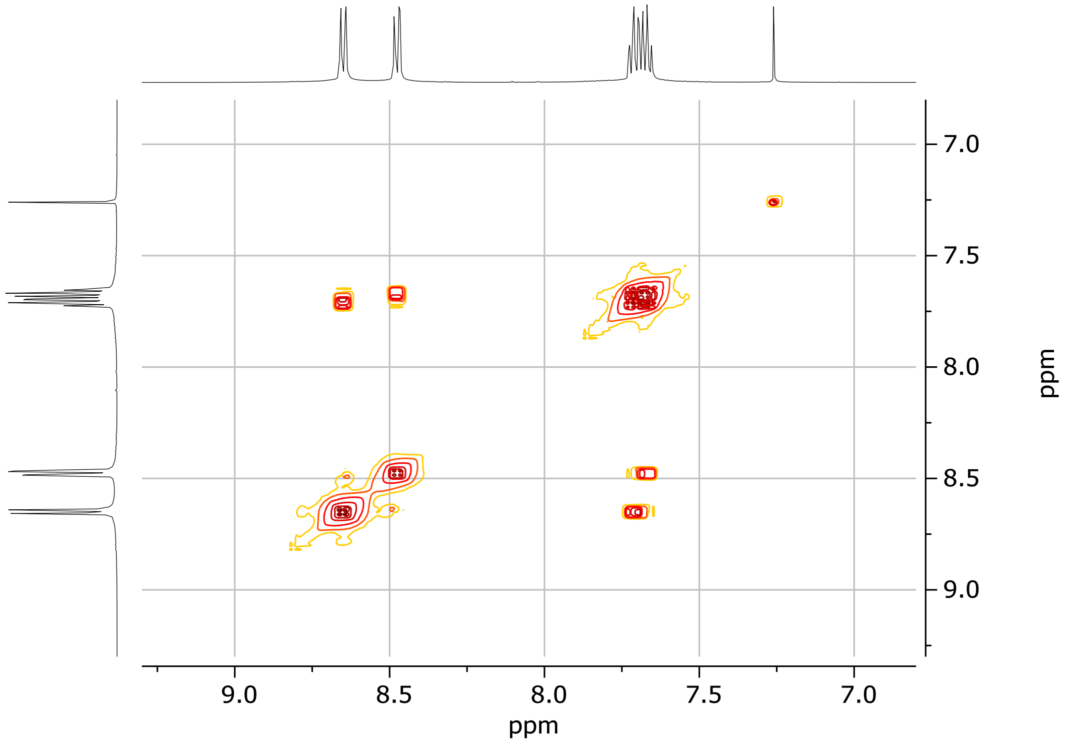

Supplementary Figure 40: COSY NMR (500 MHz) of **18** in $CDCl_3$.

Compound 19

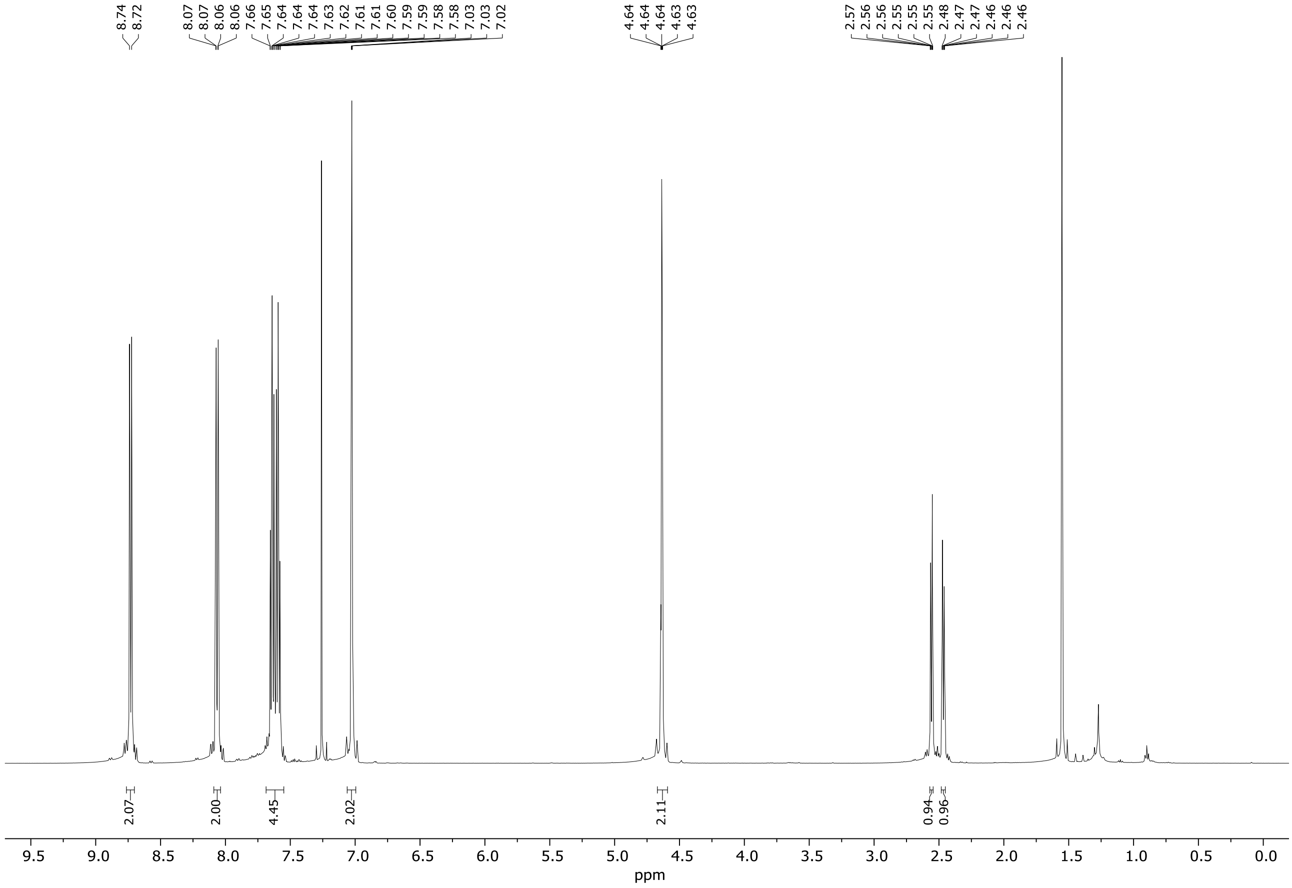

Supplementary Figure 41: ^{1}H NMR (500 MHz) of **19** in $CDCl_3$.

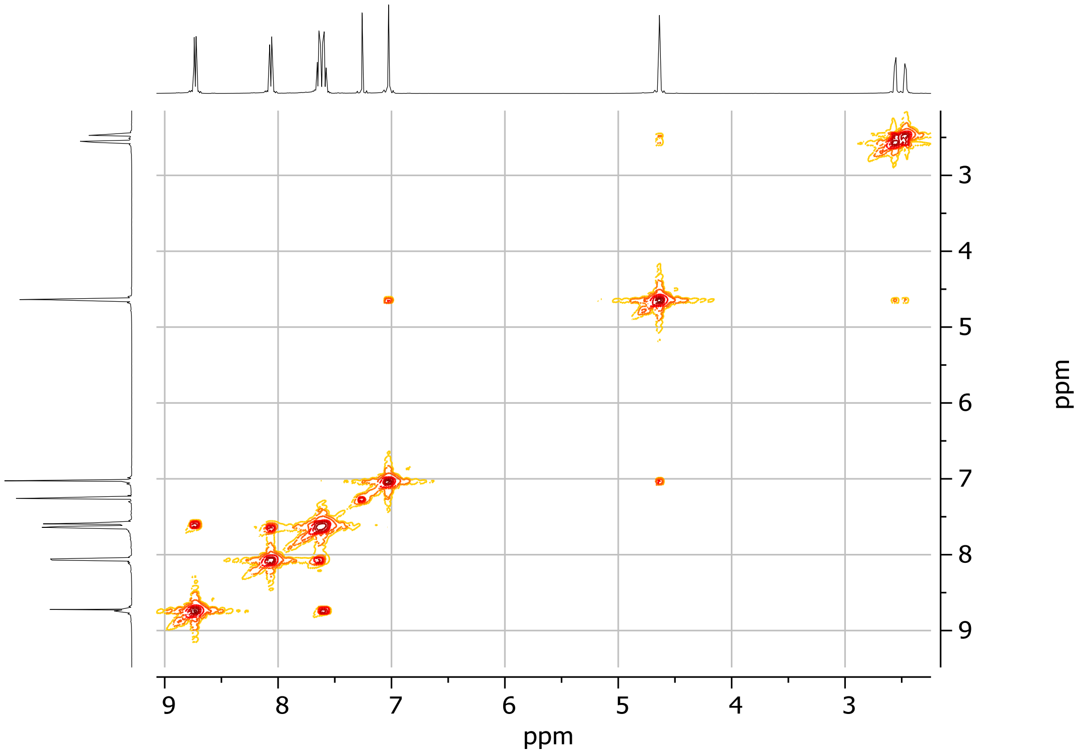

Supplementary Figure 42: COSY NMR (500 MHz) of **19** in $CDCl_3$.

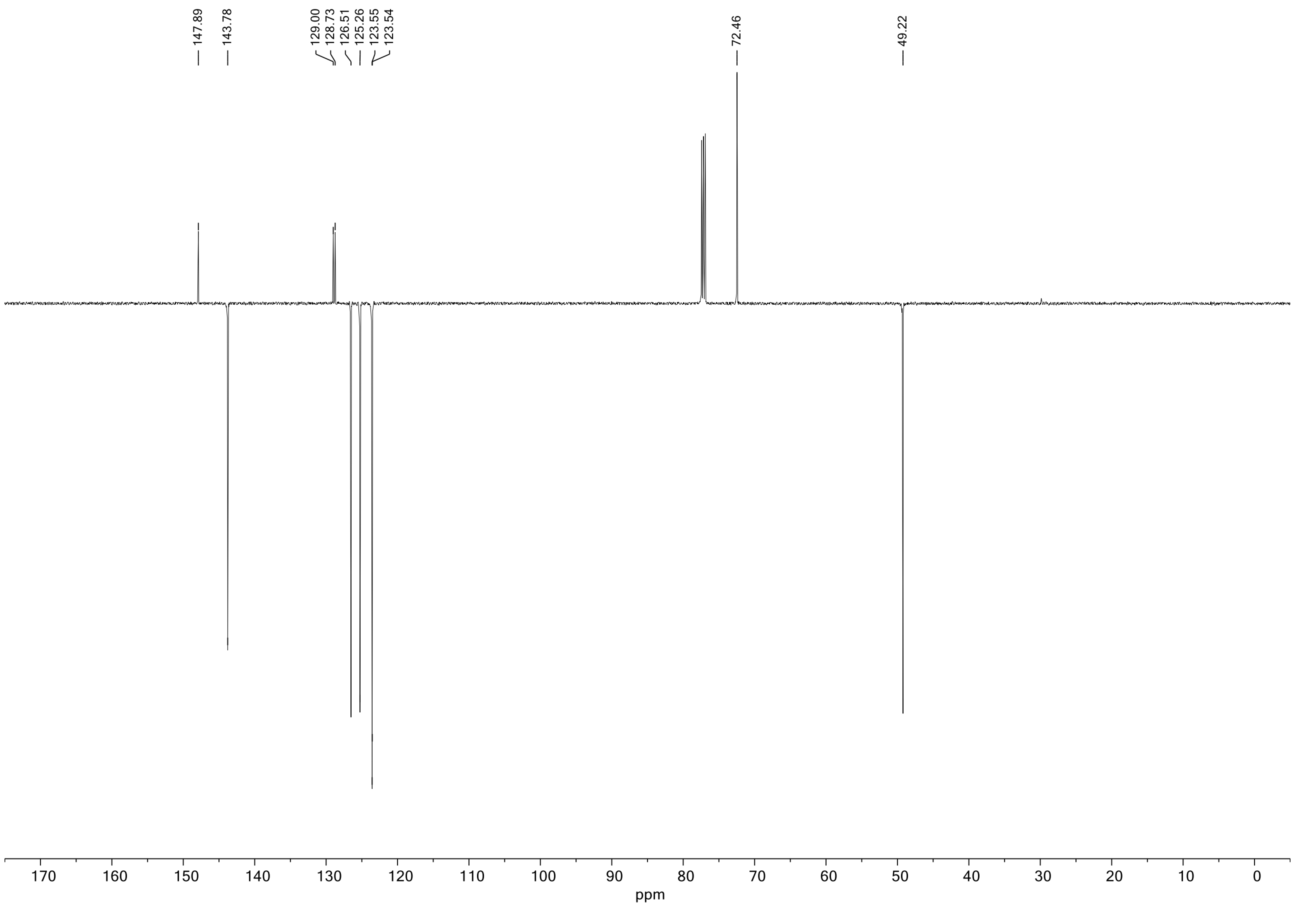

Supplementary Figure 43: ^{13}C NMR (126 MHz) of **19** in $CDCl_3$.

Compound 21

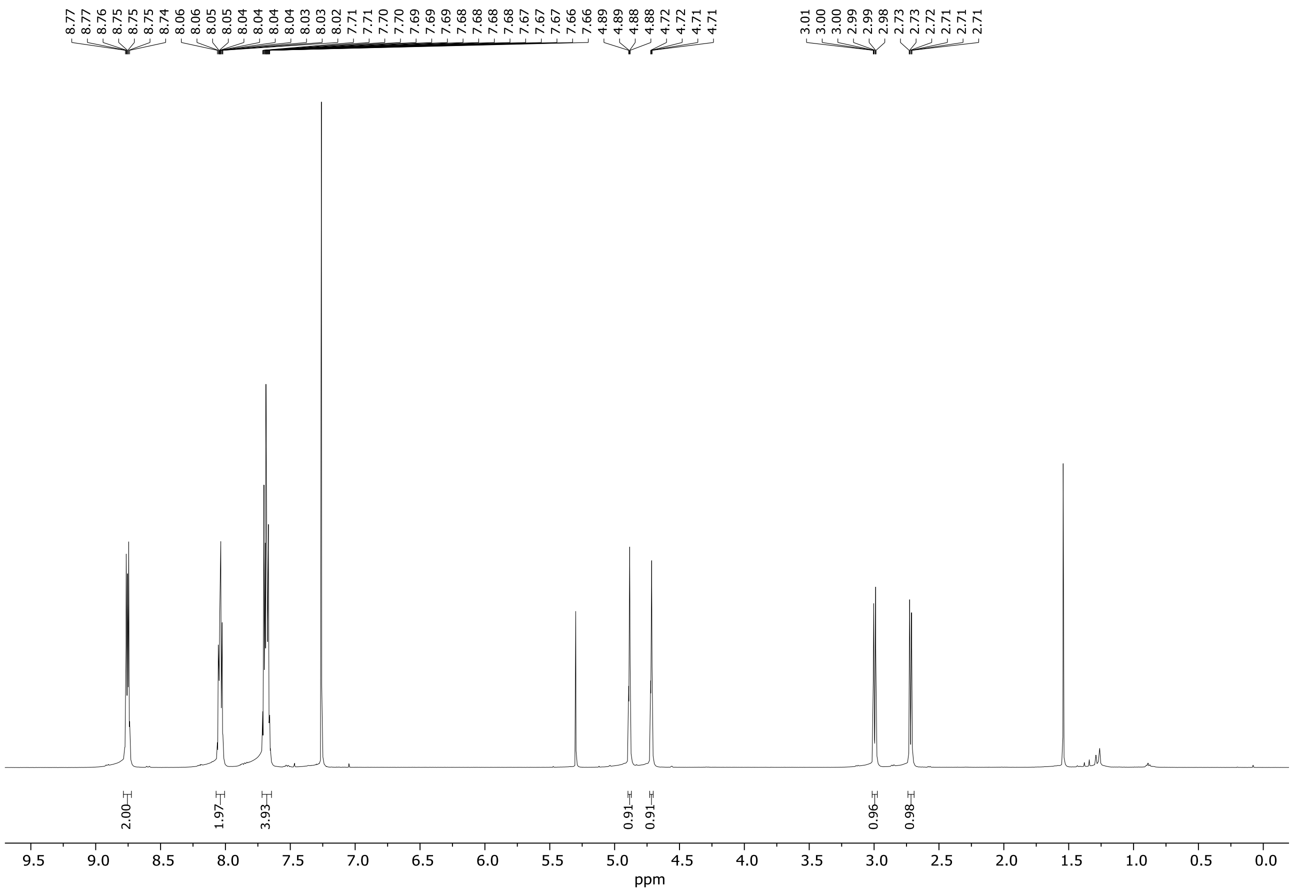

Supplementary Figure 44: ^{1}H NMR (500 MHz) of **21** in $CDCl_3$.

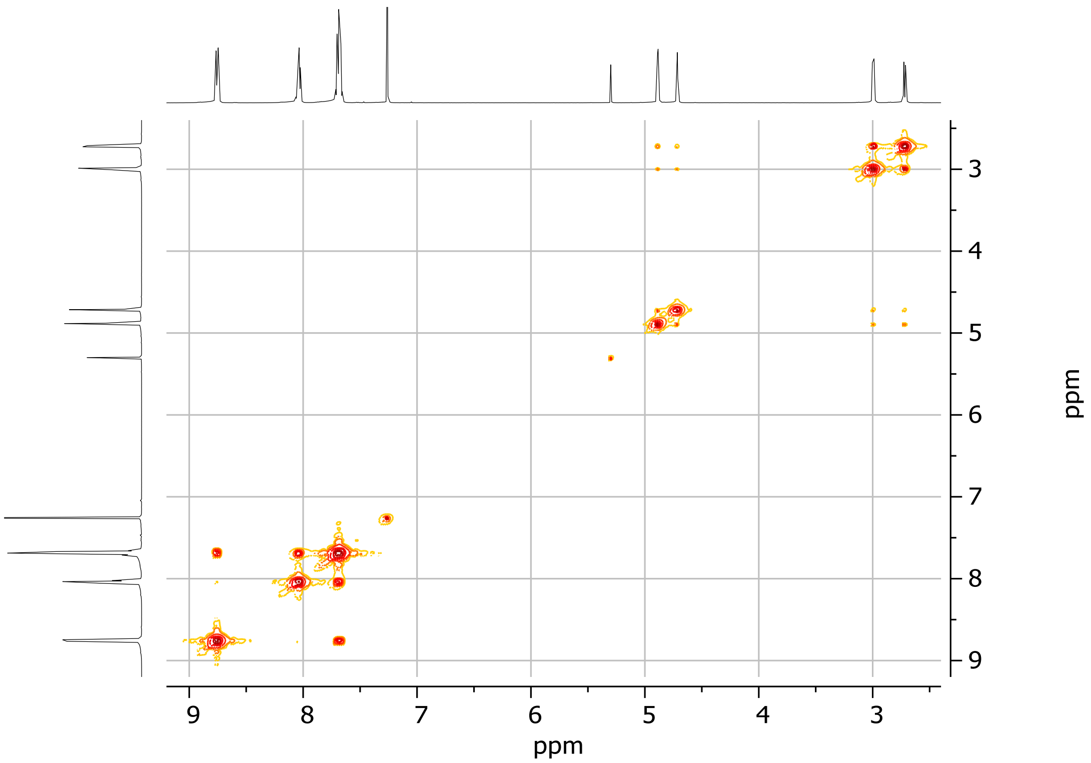

Supplementary Figure 45: COSY NMR (500 MHz) of **21** in $CDCl_3$.

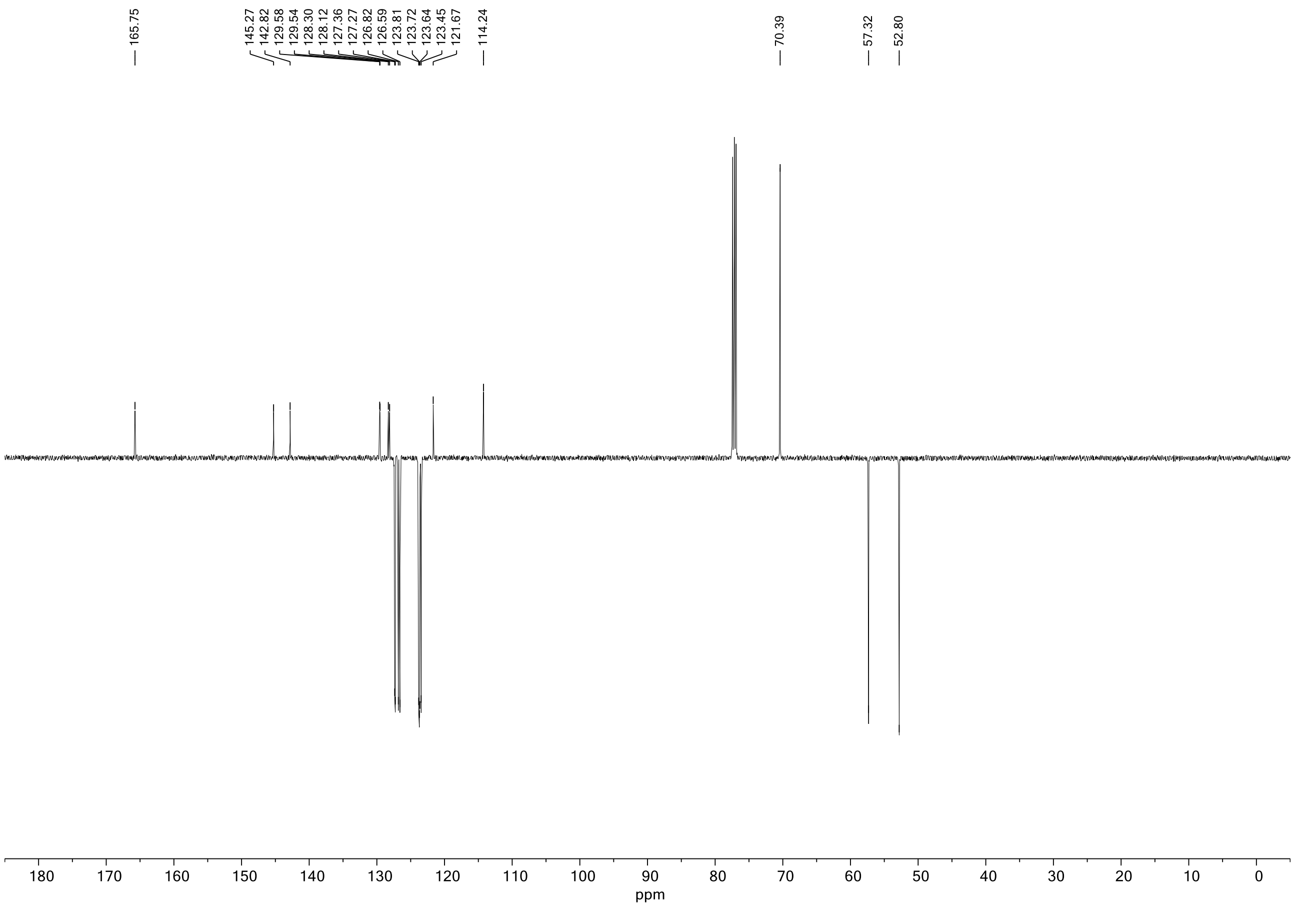

Supplementary Figure 46: ^{13}C APT NMR (126 MHz) of **21** in $CDCl_3$.

Compound 22

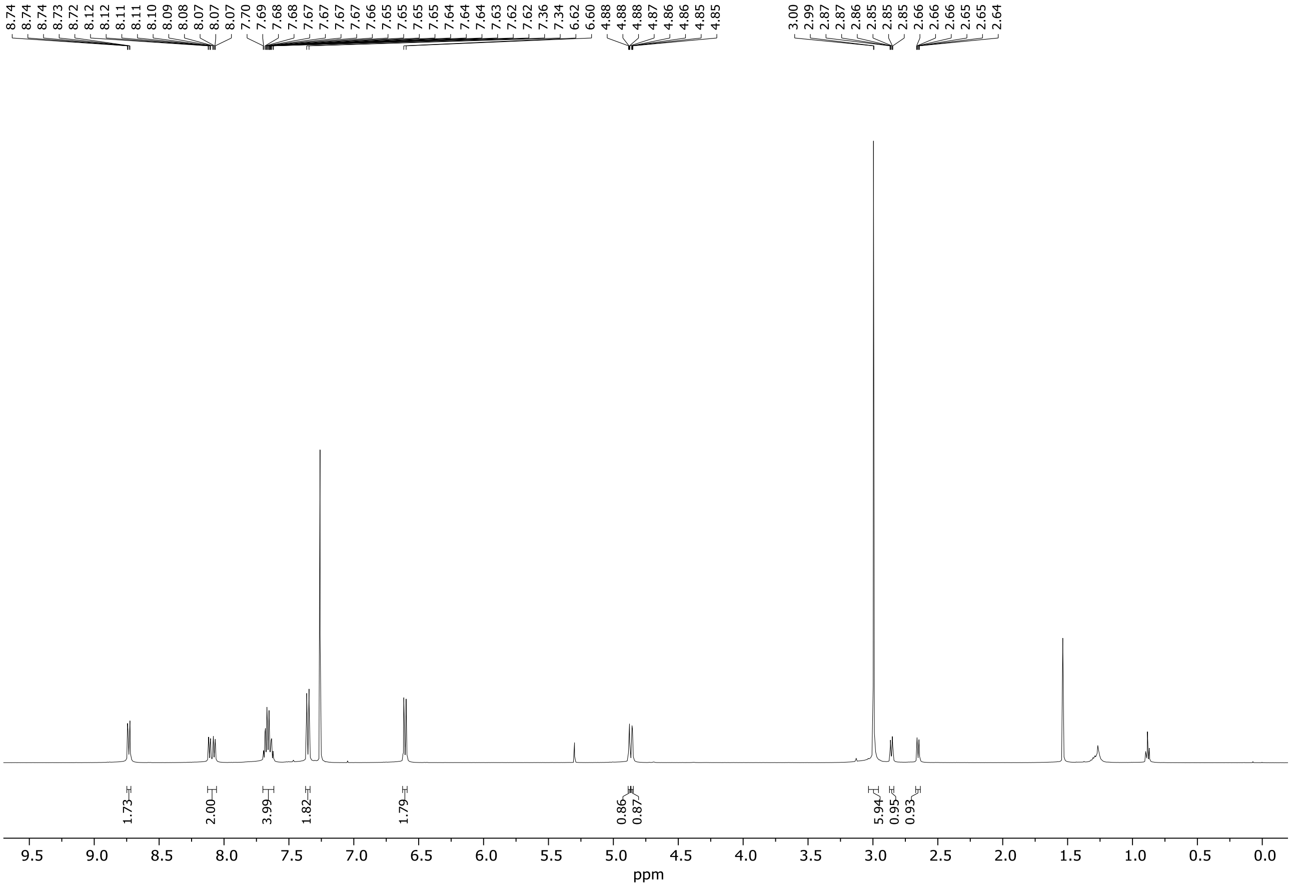

Supplementary Figure 47: ^{1}H NMR (500 MHz) of **22** in $CDCl_3$.

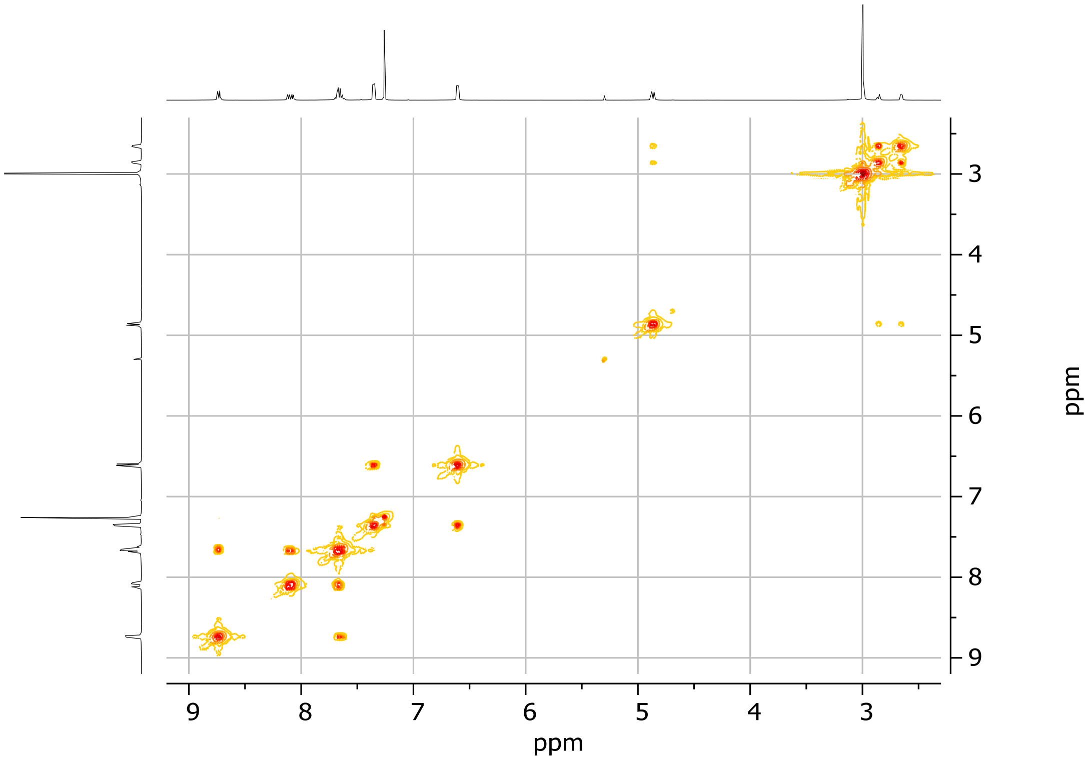

Supplementary Figure 48: COSY NMR (500 MHz) of **22** in $CDCl_3$.

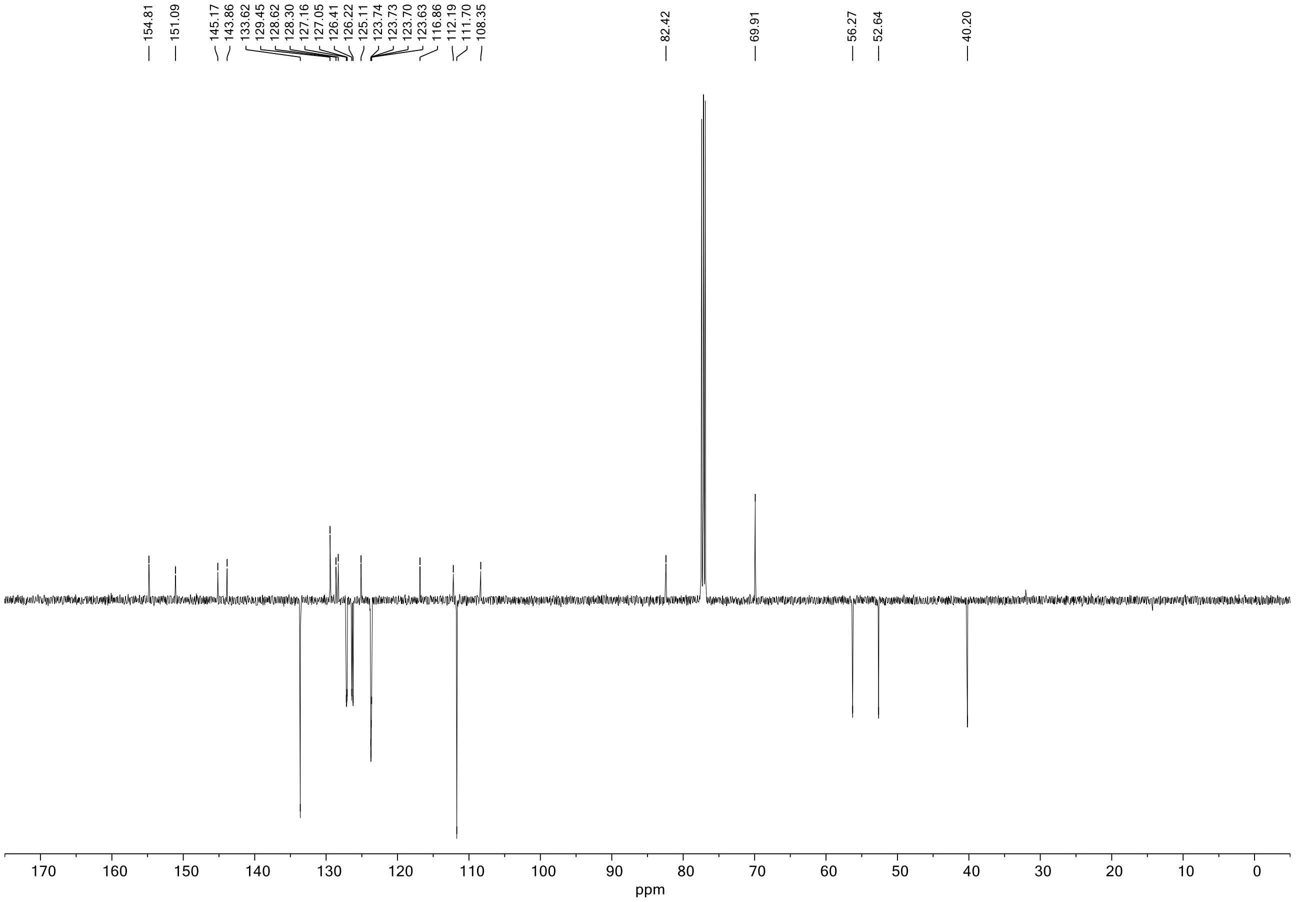

Supplementary Figure 49: ^{13}C APT NMR (126 MHz) of **22** in $CDCl_3$.

Compound 23

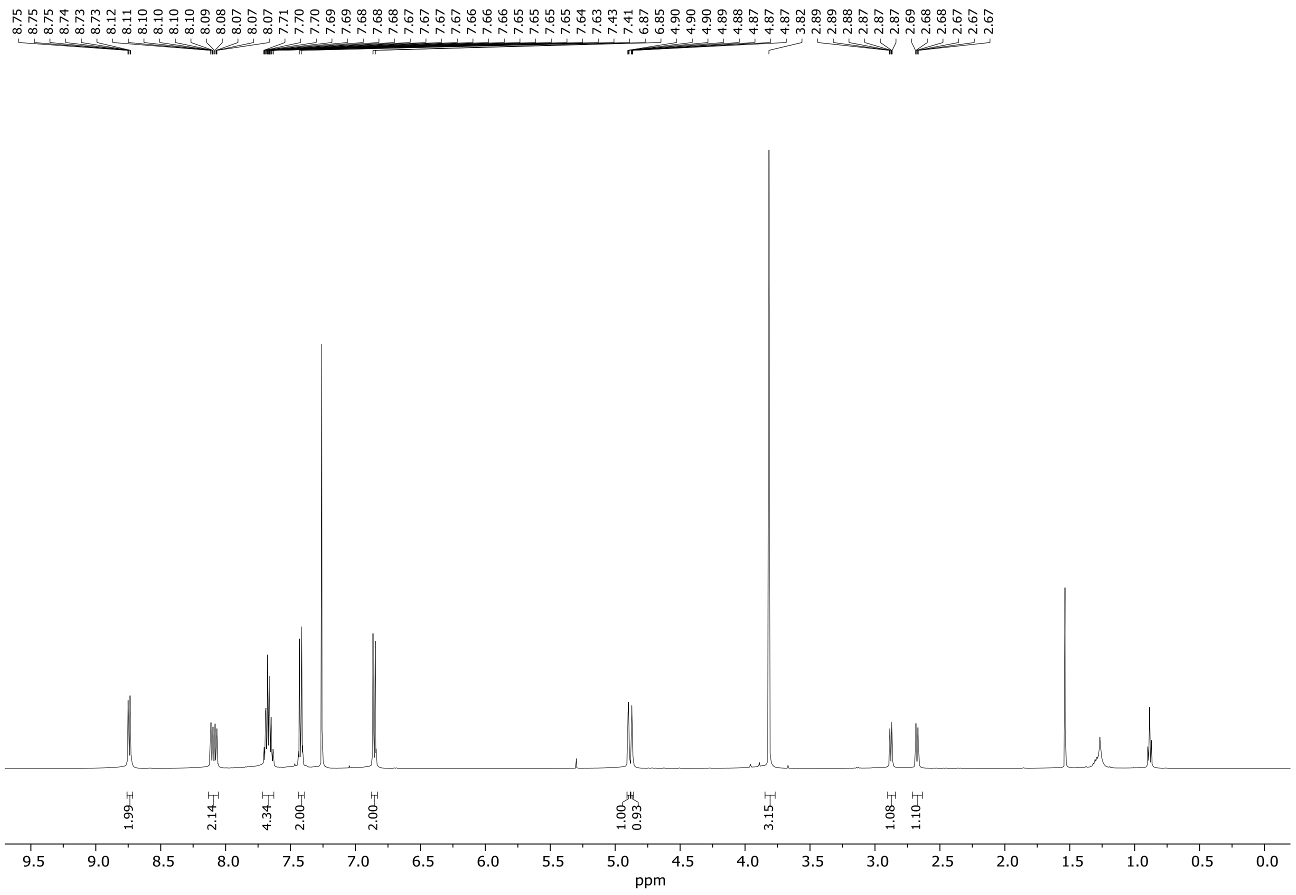

Supplementary Figure 50: ^{1}H NMR (500 MHz) of **23** in $CDCl_3$.

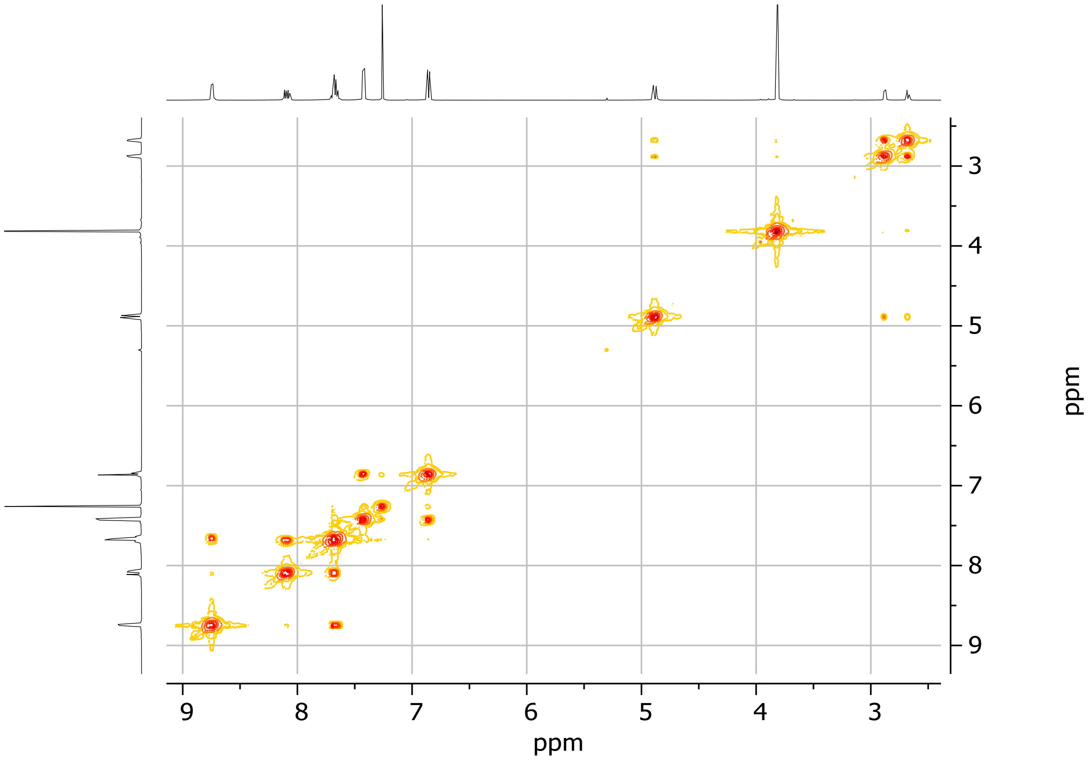

Supplementary Figure 51: COSY NMR (500 MHz) of **23** in $CDCl_3$.

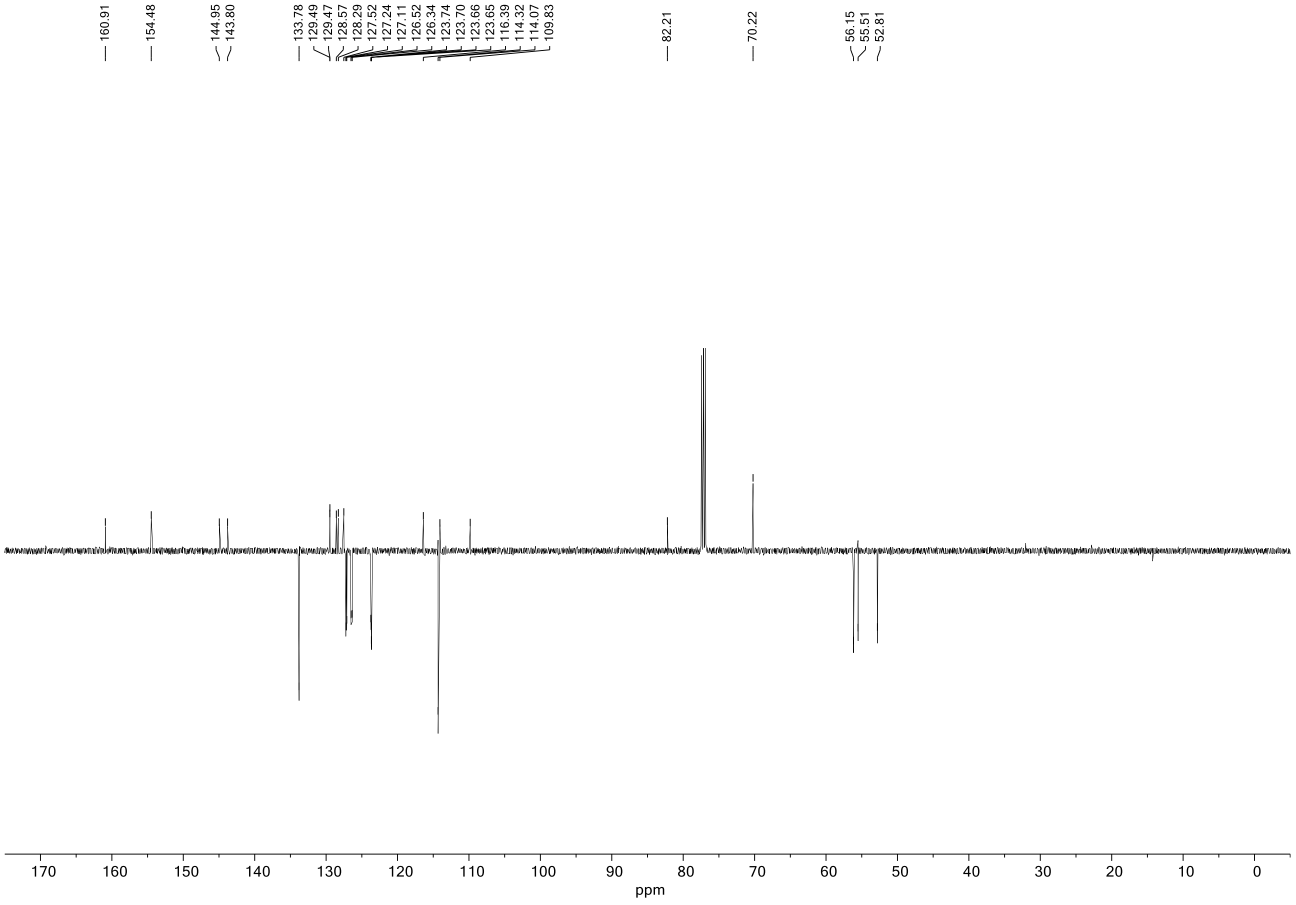

Supplementary Figure 52: ^{13}C APT NMR (126 MHz) of **23** in $CDCl_3$.

Photosensitizer Experiments

Compound **22** (2.3 mg) was dissolved in toluene-d_8 (600 µL) in an NMR tube; about the same weight of the Michler's ketone sensitizer was then added. The sample was then irradiated with a 300 nm LED light source from Thorlabs overnight (M300L4, minimum power output 32 mW). The NMR spectrum of the pure sample with Michler's ketone sensitizer before irradiation and after irradiation overnight was taken (Supplementary Figure 50).

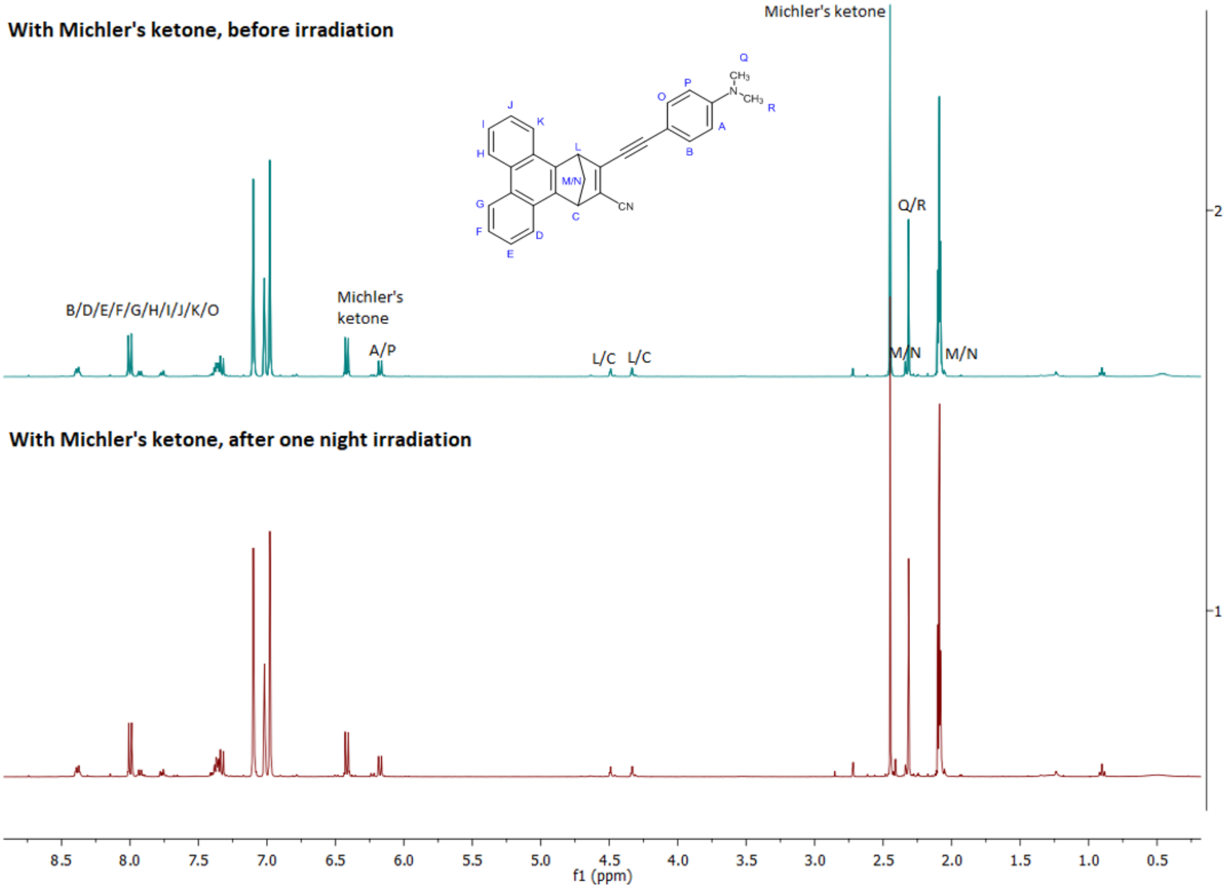

Supplementary Figure 50. NMR spectra of **22** together with Michler's ketone in toluene-d_8 before (top) and after (bottom) irradiation.

Compound **23** (2.6 mg) was dissolved in toluene-d8 (600 µL) in an NMR tube; about the same weight of the Michler's ketone sensitizer was then added. The sample was then irradiated with a 300 nm LED light source from Thorlabs overnight (M300L4, minimum power output 32 mW). The NMR spectrum of the pure sample with Michler's ketone sensitizer before irradiation and after irradiation overnight was taken (Supplementary Figure 51).

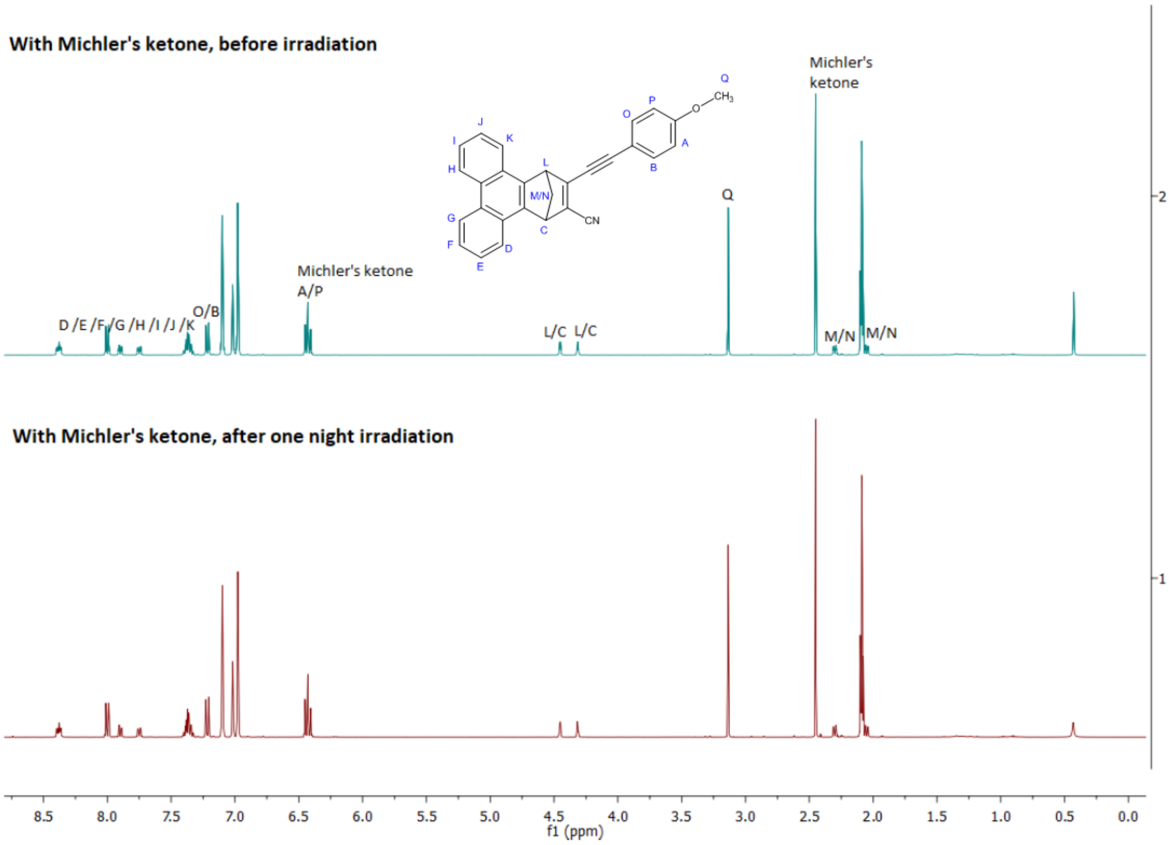

Supplementary Figure 51. NMR spectra of **23** together with Michler's ketone in toluene-d8 before (top) and after (bottom) irradiation.
